# m^6^A deficiency induces dopaminergic neurodegeneration and progressive parkinsonism through a pathogenic loop with mitochondria

**DOI:** 10.1172/JCI197183

**Published:** 2026-03-17

**Authors:** Sun Liu, Qihuan Ren, Guiling Mo, Zengguang Li, Huili Huang, Yuhao Zhou, Ziteng Miao, Xin Cao, Bilian Wu, Zhuoyu Xiao, Shihui Yu, Guangjin Wu, Linjian Xia, Jinru Cui, Junyuan Mo, Yuan Li, Laixin Xia, Juan Shen, Shan Xiao

**Affiliations:** 1 Department of Developmental Biology, School of Basic Medical Sciences, Southern Medical University, Guangzhou, China.; 2 Guangzhou KingMed Diagnostics Group Co. Ltd., International Biotech Island, Guangzhou, China.; 3 State Key Laboratory of Multi-organ Injury Prevention and Treatment, Southern Medical University, Guangzhou, China.; 4 Key Laboratory of Mental Health of the Ministry of Education, School of Basic Medical Sciences, Southern Medical University, Guangzhou, China.; 5 School of Bioscience and Biopharmaceutics, Guangdong Province Key Laboratory of Pharmaceutical Bioactive Substances, Guangdong Pharmaceutical University, Guangzhou, China.

**Keywords:** Genetics, Neuroscience, Mitochondria, Parkinson disease, RNA processing

## Abstract

Despite substantial progress in understanding the molecular pathology of Parkinson’s disease (PD), the underlying drivers of PD in many cases remain unknown. Here, we investigate the role of RNA modification in PD, following observations of selective m^6^A hypomethylation in the substantia nigra (SN) of mouse PD models and dysregulated *METTL3* and *ALKBH5* expression in dopaminergic (DA) neurons from patients with PD. We found preferential m^6^A deposition on transcripts of PD risk genes and what we believe to be a previously unreported heterozygous METTL3 p.K480R mutation in patients with PD. *Mettl3*^K480R/+^ mice exhibited progressive METTL3 reduction and m^6^A hypomethylation in the SN, leading to progressive DA neuron loss, phospho-α-synuclein increase, and levodopa-responsive motor and nonmotor deficits, mimicking PD progression. Dopamine transporter–specific METTL3 knockout mice recapitulate m^6^A hypomethylation, neurodegeneration, and levodopa-responsive parkinsonism. Mechanistically, m^6^A deficiency disrupted mitochondrial biogenesis and function through regulating *Tfam* expression, while mitochondrial dysfunction reciprocally impaired m^6^A deposition, creating a pathogenic loop. Importantly, supplementation with S-adenosylmethionine (SAMe) enhanced m^6^A modification, disrupted the pathogenic loop, and alleviated parkinsonism in mouse models. Our findings revealed m^6^A dysregulation as an important contributor to PD pathogenesis, provide a valuable preclinical mouse model for PD progression, and highlight RNA methylation-targeted therapies as a promising strategy for PD intervention.

## Abstract 2

RNA m6A deficiency drives Parkinson’s neurodegeneration through a pathogenic loop with mitochondria. Correcting m6A dysfunction breaks this loop to alleviate disease progression.

## Introduction

Parkinson’s disease (PD) is the second most common neurodegenerative disorder, characterized by the progressive degeneration of dopaminergic (DA) neurons in the nigrostriatal pathway (1). The loss of these neurons results in a variety of motor symptoms, including tremors, rigidity, and bradykinesia, as well as nonmotor symptoms such as constipation, depression, and cognitive decline (2, 3). The onset and progression of PD are influenced by a complex interplay of genetic, epigenetic, and environmental factors (4–9), and its etiology and pathogenesis remain incompletely understood. Specifically, the role of epitranscriptomic alterations in PD is still poorly defined.

N(6)-methyladenosine (m^6^A) is the most abundant modification in mRNA (10). The METTL3-METTL14-WTAP methyltransferase complex facilitates m^6^A modifications (11, 12), while the demethylases FTO and ALKBH5 are responsible for their removal (13, 14). m^6^A plays critical roles in various biological processes, including transcription (15), RNA structure (16), mRNA stability (17), mRNA processing (18–20), mRNA translation (21), DNA damage response (22), and histone modifications (15, 23). In mammals, m^6^A is essential for neurogenesis and neurological diseases such as Alzheimer’s disease and C9ORF72-ALS/FTD (24–27). Recent studies have suggested a link between m^6^A and PD through the analysis of peripheral blood mononuclear cells from patients with PD and the use of PD rat models (28, 29). However, it remains to be elucidated whether m^6^A deficiency could be a causal factor for PD in vivo and what specific mechanisms might be involved in dopamine neurons.

In this study, we describe a mechanistic link between m^6^A modification deficiency and PD and identify a rare heterozygous METTL3 p.K480R mutation in patients with PD. We show that *Mettl3*^K480R/+^ and *Mettl3*^loxp/loxp^; DAT-Cre mice develop neurodegeneration and parkinsonism through a pathogenic loop of aberrant m^6^A and mitochondrial dysfunction. Administration of S-adenosylmethionine (SAMe) in mouse models partially rescued parkinsonism. This study provides insights into the pathogenesis of PD and identifies potential therapeutic strategies.

## Results

### m^6^A modification deficiency is associated with PD.

**Figure 1:**
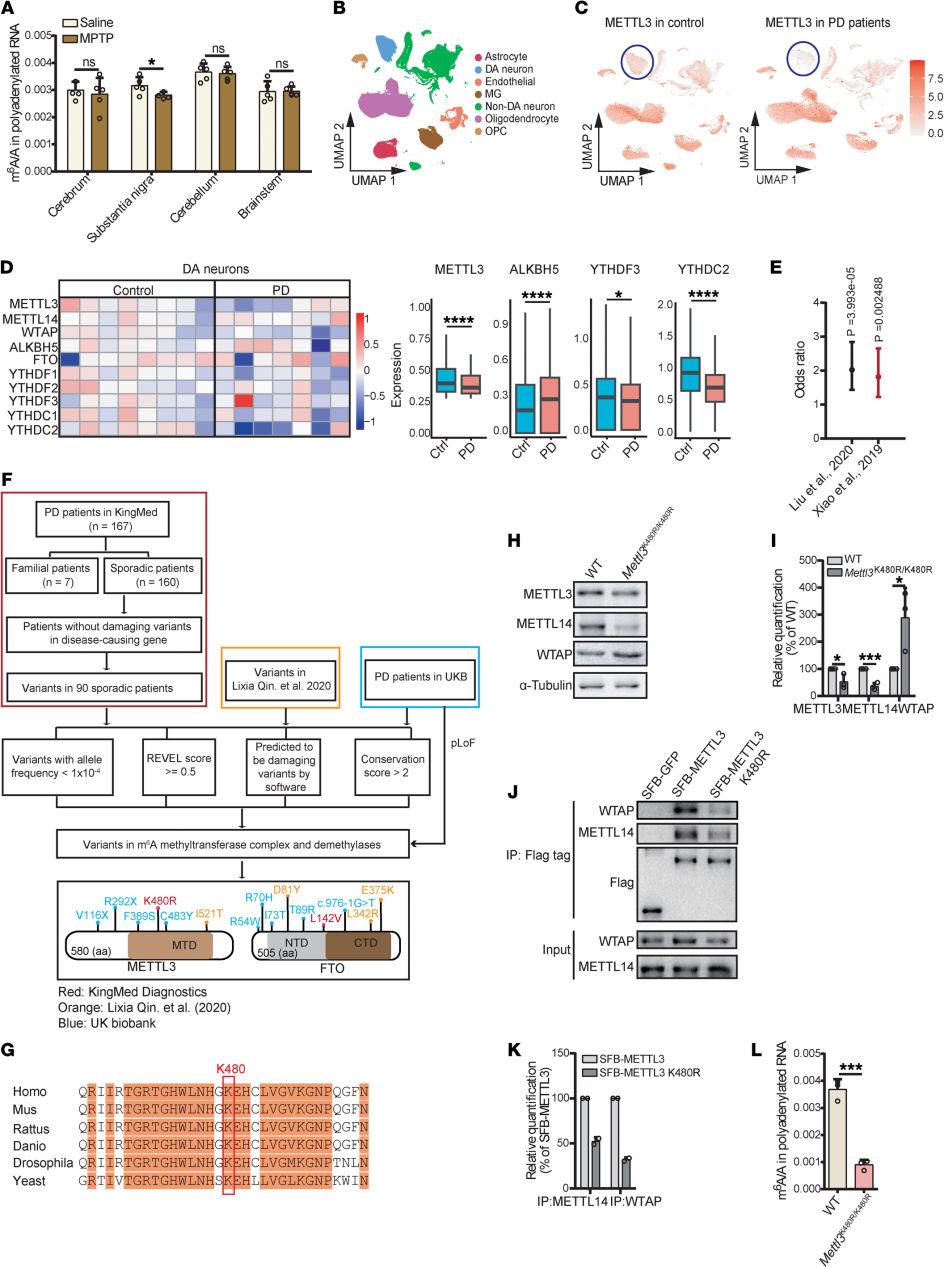
m^6^A modification deficiency is associated with PD. (**A**) LC-MS/MS quantification of mRNA m^6^A abundance in different brain regions from saline- or MPTP-treated mice (*n* = 5). (**B** and **C**) UMAP reanalysis of published snRNA-seq data (accession number GSE178265) (**B**) and UMAP visualization of METTL3 expression (**C**) from the SNc of patients with PD and matched individuals acting as controls. Color intensity indicates relative expression levels. (**D**) Heatmap showing the average expression levels of m^6^A regulators in DA neurons from individuals acting as controls (*n* = 15,458 from 8 individuals) and patients with PD (*n* = 2,430 from 7 individuals) (left). Box plots showing differential expression of m^6^A regulators in DA neurons of patients with PD versus individuals acting as controls (right). (**E**) Enrichment of m^6^A deposition on transcripts of PD risk genes by Fisher’s exact test. PD risk genes were obtained from DisGeNET using the identifier Parkinson disease (C0030567). The human brain meRIP-seq data were taken from 2 published studies (32, 33). (**F**) Whole-exome sequencing (WES) data analysis pipeline. Schematic diagram showing mutation sites in m^6^A methyltransferase and demethylase genes identified in patients with PD. pLoF, predicted loss of function. (**G**) Sequence alignment of METTL3 showing conservation of K480. (**H**) Immunoblot showing the protein level of METTL3, METTL14, and WTAP from WT and *Mettl3*^K480R/K480R^ mESCs. (**I**) Protein quantification corresponding to **H** (*n* = 3). (**J**) Co-IP showing the interaction of WTAP and METTL14 with SFB-tagged METTL3 or METTL3 K480R. (**K**) Protein quantification corresponding to **J** (*n* = 2 independent biological samples per group). (**L**) LC-MS/MS quantification of m^6^A abundance in mRNA from WT and *Mettl3*^K480R/K480R^ mESCs (*n* = 3). Data are shown as the mean ± SD; Wilcoxon’s rank-sum test (**D**), Fisher’s exact test (**E**), and 2-tailed Student’s *t* test (**I** and **L**). *P < 0.05, ***P < 0.001, ****P < 0.0001.

To investigate whether m^6^A is dysregulated in PD, we generated an MPTP-induced mouse model (30) and examined mRNA m^6^A levels across multiple brain regions. We observed a significant decrease in m^6^A levels selectively in the SN, a critical region implicated in motor function and PD pathology (Figure 1A). Next, we analyzed publicly available single-nucleus RNA-seq (snRNA-seq) data from the substantia nigra pars compacta (SNc) of patients with PD and matched individuals acting as controls (31). Among the cell clusters (Figure 1B), we found that the expression level of m^6^A methyltransferase METTL3 was significantly downregulated in the DA neurons of patients with PD (Figure 1C), demethylase ALKBH5 was upregulated, and readers YTHDF3 and YTHDC2 were reduced (Figure 1D and Supplemental Figure 1A; supplemental material available online with this article; https://doi.org/10.1172/JCI197183DS1). These observations suggest a disruption of the m^6^A machinery in the DA neurons of patients with PD. Integrative analysis of the differentially expressed genes (DEGs) in DA neurons of patients with PD and m^6^A modifications in the brain (32, 33) revealed that the DEGs were markedly more likely to be modified by m^6^A (Supplemental Figure 1B). DEGs that were downregulated in DA neurons of patients with PD with m^6^A modification are predominantly enriched in mitochondrial-related pathways (Supplemental Figure 1C), which are known to play a pivotal role in PD pathogenesis (34). Inclusion of PD risk genes from DisGeNET (35) further revealed preferential m^6^A deposition on PD risk gene transcripts (Figure 1E), suggesting that m^6^A may be involved in the regulation of these genes.

To gain insight into whether m^6^A dysregulation is associated with PD, we screened the genetic variants in patients with PD for mutations that may disrupt m^6^A deposition. Through stringent filtering, we identified 15 variants from 3 groups of patients with PD (36) located in either *METTL3* or *FTO* (Figure 1F and Supplemental Figure 1D). The c.1439A>G, p.K480R mutation in *METTL3* was identified as a heterozygous mutation in 1 patient with PD (Figure 1F), which was not reported in GnomAD, ExAC, or 1000 Genomes Project databases. Cross-species analysis of METTL3 proteins revealed the evolutionary conservation of the K480 residue (Figure 1G). Structural analysis of the METTL3-METTL14 complex (Protein Data Bank ID code 5IL1) (37) showed that K480 is situated in close proximity to the interface between METTL3 and METTL14 (Supplemental Figure 1E), suggesting that it may be functionally important.

To explore the consequences of this mutation, we generated a *Mettl3*^K480R/K480R^ mutant in mouse embryonic stem cells (mESCs) using CRISPR/Cas9 (Supplemental Figure 1, F–H). In the mutant cells, we observed reduced protein levels of METTL3 and METTL14, while the level of WTAP increased (Figure 1, H and I). Although the subcellular localization of the 3 methyltransferase complex components remained largely unaltered (Supplemental Figure 1, I and J), the interaction between METTL14 and METTL3 was reduced (Figure 1, J and K). A substantial decrease in the m^6^A/A ratio was observed in *Mettl3*^K480R/K480R^ cells compared with that in WT cells (Figure 1L). Collectively, these findings indicated that the METTL3 K480R variant impairs m^6^A methylation, which provides an entry point to investigate how m^6^A deficiency contributes to PD pathogenesis.

### Mettl3^K480R/+^ mice exhibit progressive m^6^A hypomethylation and DA neurodegeneration.

**Figure 2:**
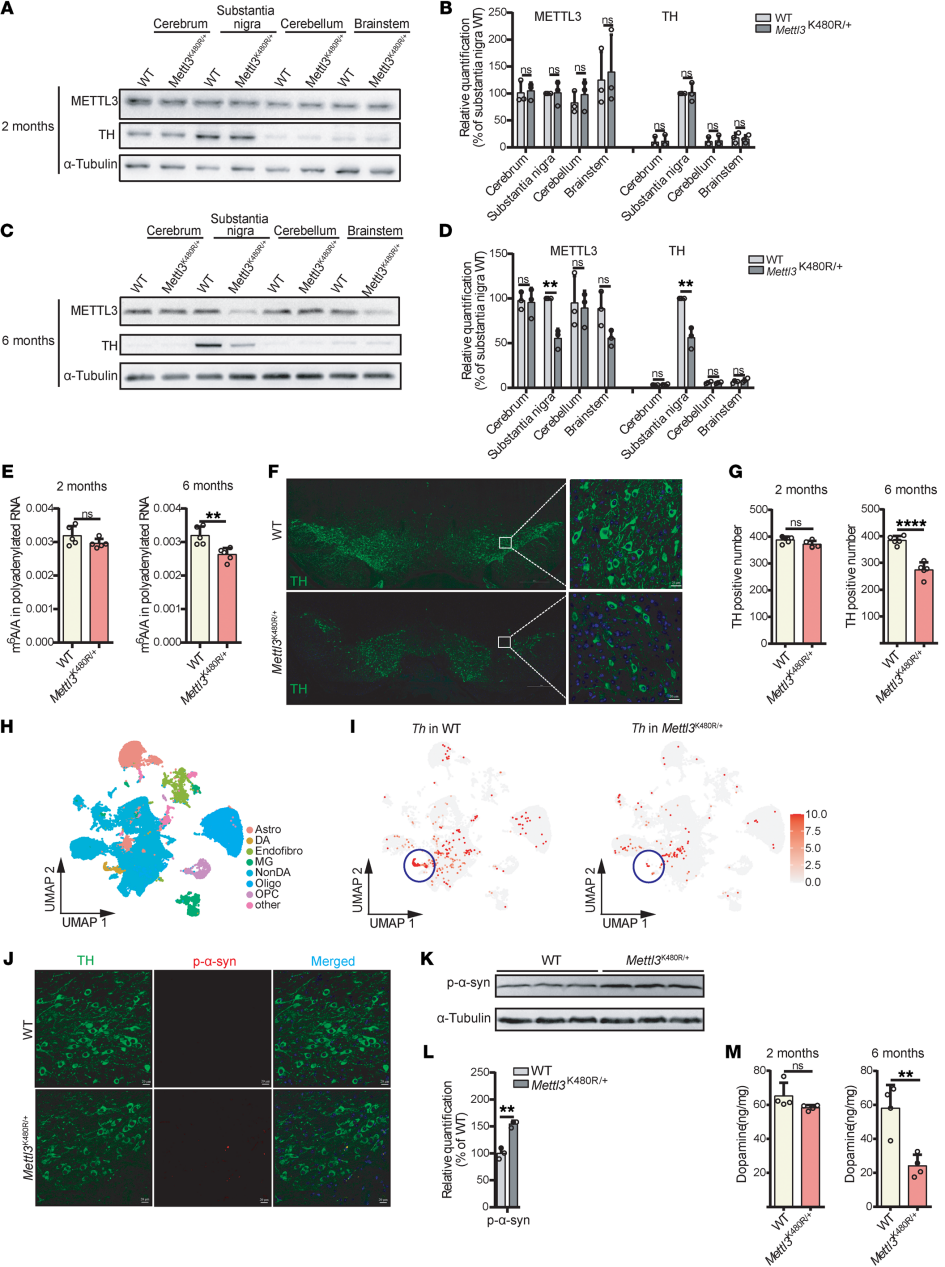
*Mettl3*^K480R/+^ mice exhibit progressive m^6^A hypomethylation and DA neurodegeneration. (**A**–**D**) Immunoblot showing the protein level of METTL3 and TH in different regions of brain from WT and *Mettl3*^K480R/+^ mice (**A**, 2 months; **C**, 6 months). Protein quantification of indicated proteins (**B**, 2 months; **D**, 6 months; *n* = 3). (**E**) LC-MS/MS quantification of mRNA m^6^A abundance in the SN (*n* = 5. Left, 2 months; Right, 6 months). (**F**) Representative images showing TH staining in the brain of 6-month-old mice. Scale bars: 500 μm (left); 20 μm (right). (**G**) Quantitative data of TH-positive neurons (*n* = 5; left, 2 months; right, 6 months). (**H**) UMAP analysis of snRNA-seq data from SN of 6-month-old mice. (**I**) UMAP visualization of *Th* expression. Color intensity indicates relative expression levels. (**J** and **K**) Immunofluorescence (**J**) and immunoblot (**K**) showing phospho-α-synuclein levels in the SN. Scale bars: 20 μm (**J**). (**L**) Protein quantification corresponding to **K** (*n* = 3). (**M**) Dopamine concentrations in the SN measured by HPLC (*n* = 4; left, 2 months; right, 6 months). Data are shown as the mean ± SD; 2-tailed Student’s *t* test. **P < 0.01, ****P < 0.0001.

To investigate the effects of the METTL3 K480R mutation on PD progression, we generated METTL3 K480R knockin mice using CRISPR/Cas9 editing (Supplemental Figure 2, A and B). A cross with heterozygous mice revealed that homozygous K480R mutants were embryonic lethal, while the ratio of heterozygous to WT offspring followed Mendelian expectations, suggesting no increased embryonic or neonatal lethality in *Mettl3*^K480R/+^ mice (Supplemental Figure 2C). Given the progressive nature of PD, we examined *Mettl3*^K480R/+^ mice at 2 time points: 2 and 6 months of age. Although METTL3 protein levels in 2-month-old mice were comparable to those of WT controls, a significant reduction was observed in the SN of 6-month-old *Mettl3*^K480R/+^ mice (Figure 2, A–D). In addition, a marked reduction in m^6^A levels of polyadenylated RNA in the SN of 6-month-old mice was observed, with no significant change at 2 months (Figure 2E). These findings indicate that the K480R heterozygous mutation leads to progressive METTL3 reduction and m^6^A deficiency in the SN of mice.

Next, we assessed the effect of the METTL3 K480R mutation on DA function. Tyrosine hydroxylase (TH), an essential enzyme in dopamine synthesis, showed no substantial change in 2-month-old *Mettl3*^K480R/+^ mice. However, a clear reduction in TH expression was evident in the SN of the 6-month-old mutants (Figure 2, A–D). This finding corresponds with a progressive decline in DA neurons in the SN of Mettl3^K480R/+^ mice, with TH-positive neurons decreasing from 93.8% to 69.5% compared with WT mice (Figure 2, F and G). To independently validate these findings at single-cell resolution, we performed snRNA-seq on the SN of 6-month-old WT and *Mettl3*^K480R/+^ mice. Unbiased clustering and marker-based annotation identified a DA neuron population characterized by expression of canonical markers *Th* and *Slc6a3*. Comparative analysis revealed a marked reduction in the *Th*-positive DA neurons from *Mettl3*^K480R/+^ mice relative to WT controls (Figure 2, H and I), while other cell types were not much changed. We next examined α-synuclein deposition in the SN of 6-month-old Mettl3^K480R/+^ mice. Immunofluorescence and immunoblotting analyses showed increased phospho-α-synuclein Ser129 levels in the SN of Mettl3^K480R/+^ mice, a hallmark of synucleinopathies (38) (Figure 2, J–L). Additionally, dopamine levels in the SN mirrored the decline in TH expression, showing a slight decrease in 2-month-old mutants and a more pronounced reduction (approximately 50% of WT) in 6-month-old mice (Figure 2M). Collectively, these findings indicate that the progressive m^6^A deficiency caused by the METTL3 K480R mutation leads to a progressive loss of DA neurons and dopamine in the SN, a hallmark feature of PD.

### Mettl3^K480R/+^ mice display levodopa-responsive parkinsonism.

**Figure 3:**
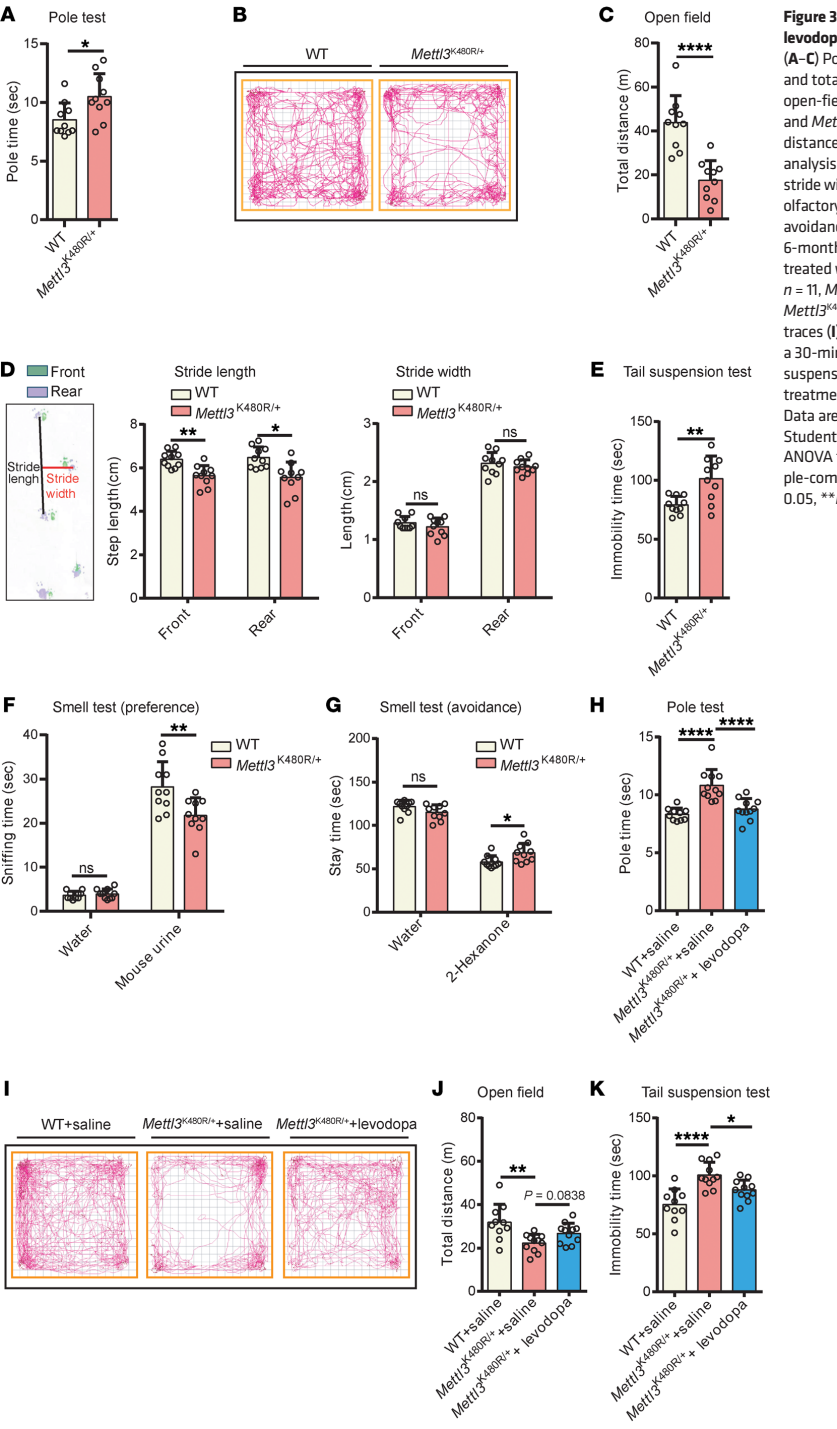
*Mettl3*^K480R/+^ mice exhibit levodopa-responsive parkinsonism. (**A**–**C**) Pole test (**A**), open-field traces (**B**), and total distance traveled in a 30-minute open-field test (**C**) in 6-month-old WT and *Mettl3*^K480R/+^ mice (*n* = 10). (**D**) Step distance (*n* = 10). Left, schematic of gait analysis; middle, stride length; right, stride width. (**E**–**G**) Tail suspension (**E**), olfactory preference test (**F**), and olfactory avoidance test (**G**) (*n* = 10). (**H**) Pole test in 6-month-old WT and *Mettl3*^K480R/+^ mice treated with levodopa (*n* = 10, WT + saline; *n* = 11, *Mettl3*^K480R/+^ + saline; and *n* = 11, *Mettl3*^K480R/+^ + levodopa). (**I**–**K**) Open-field traces (**I**), total distance traveled during a 30-minute open-field test (**J**), and tail suspension test (**K**) following levodopa treatment (same group sizes as in **H**). Data are shown as mean ± SD; 2-tailed Student’s *t* test (**A**, **C**, and **D**–**G**) and 1-way ANOVA followed by Holm-Šidák multiple-comparisons test (**H**, **J**, and **K**). *P < 0.05, **P < 0.01, ****P < 0.0001.

To assess the consequences of DA loss in Mettl3^K480R/+^ mice, we conducted a series of behavioral tests. In the pole test, 6-month-old *Mettl3*^K480R/+^ mice exhibited significantly prolonged climbing time compared with WT controls (Figure 3A), suggesting motor coordination and strength deficits. In the open-field test and step distance test, *Mettl3*^K480R/+^mice demonstrated significantly reduced locomotor activity and stride length (Figure 3, B–D). In the tail suspension test, Mettl3^K480R/+^ mice displayed significantly prolonged periods of immobility (Figure 3E), suggesting depressive-like behavior. Additionally, *Mettl3*^K480R/+^ mice demonstrated a significantly reduced tendency to sniff female mouse urine (Figure 3F), and they spent more time in the hexanone-containing area (Figure 3G), indicating hyposmia. The administration of levodopa led to a marked improvement in the motor and behavioral deficits observed in the pole, open-field, and tail suspension tests (Figure 3, H–K). These results demonstrate that *Mettl3*^K480R/+^ mice exhibit levodopa-responsive parkinsonism, which, in conjunction with the DA neurodegeneration phenotype, may make them a useful PD model.

### Mettl3 depletion in DA neurons recapitulates neurodegenerative and levodopa-responsive parkinsonism phenotypes.

**Figure 4:**
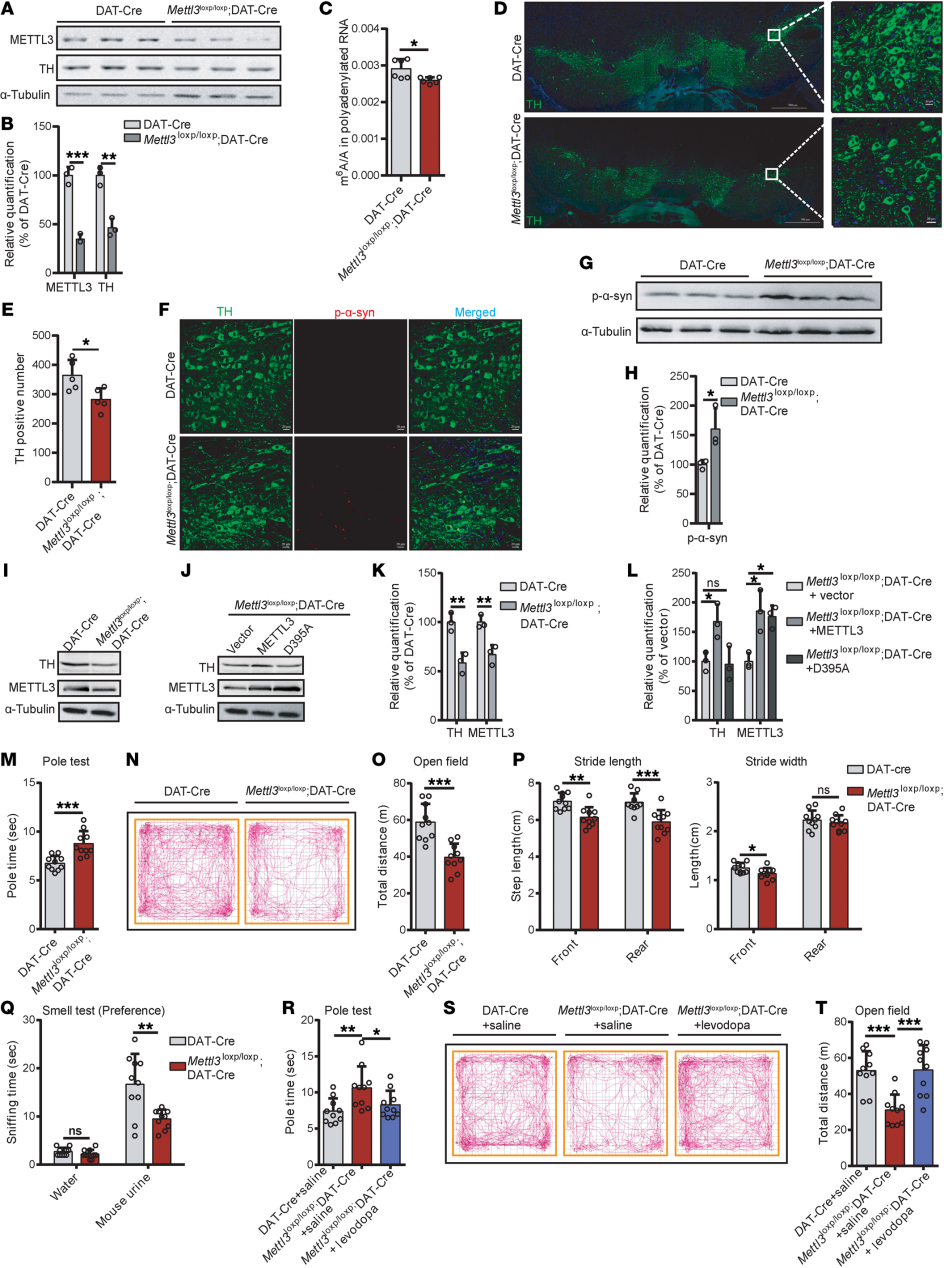
*Mettl3* depletion in DA neurons recapitulates neurodegenerative and levodopa-responsive parkinsonism phenotypes. (**A**) Immunoblot showing protein level of METTL3 and TH in the SN of 6-month-old DAT-Cre and *Mettl3*^loxp/loxp^; DAT-Cre mice. (**B**) Protein quantification corresponding to **A** (*n* = 3). (**C**) LC-MS/MS quantification of mRNA m^6^A abundance (*n* = 6). (**D** and **E**) Representative images of TH staining (**D**) and quantitation of TH-positive neurons (**E**; *n* = 5). Scale bars: 500 μm (left); 20 μm (right). (**F** and **G**) Immunofluorescence (**F**) and immunoblot (**G**) showing phospho-α-synuclein levels in the SN. Scale bars: 20 μm. (**H**) Protein quantification corresponding to **G** (*n* = 3). (**I** and **J**) Immunoblot showing METTL3 and TH level in primary DA neurons derived from fetal SN of DAT-Cre and *Mettl3*^loxp/loxp^; DAT-Cre mice (**I**) and following overexpression of vector, METTL3, or METTL3 D395A (**J**). (**K** and **L**) Quantification corresponding to **I** (**K**) and **J** (**L**) (*n* = 3). (**M**–**O**) Pole test (**M**), open-field traces (**N**), and total distance traveled in a 30-minute open-field test (**O**) (*n* = 10). (**P**) Step distance assessment (*n* = 10). Left, stride length; right, stride width. (**Q**) Olfactory preference test (*n* = 10). (**R**–**T**) Pole test (**R**), open-field traces (**S**), and total distance traveled in a 30-minutes open-field test (**T**) following levodopa treatment (*n* = 10). Data are shown as the mean ± SD; 2-tailed Student’s *t* test (**B**, **C**, **E**, **H**, **K**, **M**, and **O**–**Q**) and 1-way ANOVA followed by Holm-Šidák multiple-comparisons test (**L**, **R**, and **T**). *P < 0.05, **P < 0.01, ***P < 0.001, ****P < 0.0001.

To further investigate the role of m^6^A in PD, we generated mice with a conditional knockout of *Mettl3* specifically in DA neurons by crossing floxed *Mettl3* (*Mettl3*^loxp/loxp^) mice with those expressing Cre recombinase (Cre) under the dopamine transporter (DAT, encoded by *Slc6a3*) promoter (*Dat-cre^+/–^*) (7) (Supplemental Figure 3A). Efficient *Mettl3* deletion in DA neurons was confirmed by a marked reduction in METTL3 protein levels in the SN at 6 months of age (Figure 4, A and B). The m^6^A levels in *Mettl3*^loxp/loxp^; DAT-Cre mice were also significantly decreased in the SN (Figure 4C). This deletion was accompanied by a significant reduction in TH expression in the SN (Figure 4, A and B) and a 20% reduction in TH-positive DA neurons in the SN (Figure 4, D and E), consistent with the DA neuron loss observed in Mettl3^K480R/+^ mice. Increased phospho-α-synuclein Ser129 levels were also observed in the SN of *Mettl3*^loxp/loxp^; DAT-Cre mice (Figure 4, F–H).

To determine whether the reduction in TH is mediated by the loss of m^6^A modification or by METTL3 protein deficiency, we performed 2 sets of experiments. First, SH-SY5Y–derived DA neuron–like cells were treated with METTL3 enzymatic inhibitor STM2457, which decreased global m^6^A level without altering METTL3 protein abundance (Supplemental Figure 3, B–D). Inhibition of m^6^A deposition resulted in reduced TH level (Supplemental Figure 3, C and D), demonstrating that suppression of m^6^A is sufficient to recapitulate TH reduction. Second, we performed a rescue assay in primary DA neurons derived from fetal mice. Given that the reduction in METTL3 protein and m^6^A level in *Mettl3*^K480R/+^ mice are age-dependent and not detectable even at 2 months, DA neurons from *Mettl3*^loxp/loxp^; DAT-Cre mice were used to achieve a m^6^A-deficiency background. Overexpression of WT METTL3 significantly rescued the reductions in TH, whereas the catalytically dead mutant D395A (23) mutant had no effect (Figure 4, I–L). Similarly, in SH-SY5Y–derived DA neuron cells, shRNA-mediated knockdown of METTL3 recapitulated these effects, and overexpression of WT METTL3, but not D395A, reversed the reductions in TH (Supplemental Figure 3, E and F). These results indicate that restoration of m^6^A modification, not METTL3 abundance, is required the phenotypic rescue.

We then assessed the behavioral phenotype of *Mettl3*^loxp/loxp^; DAT-Cre mice. We observed impaired motor activity and reduced performance in the pole and open-field test (Figure 4, M–O). Gait abnormalities and olfactory deficits were also observed (Figure 4, P and Q). The administration of levodopa alleviated the motor impairments in *Mettl3*^loxp/loxp^; DAT-Cre mice (Figure 4, R–T).

Collectively, these findings demonstrate that the specific deletion of *Mettl3* in DA neurons leads to DA neurodegeneration and levodopa-responsive parkinsonism, highlighting the critical role of m^6^A modifications in DA neurons during disease progression.

### m^6^A deficiency impairs mitochondrial function.

We next aimed to elucidate the molecular mechanism underlying m^6^A deficiency–mediated PD pathogenesis. First, we analyzed the aberrantly expressed genes in the SN from *Mettl3*^loxp/loxp^; DAT-Cre mice and in *Mettl3*^K480R/K480R^ mESCs, finding that approximately half of the protein-coding genes encoded by the mitochondrial genome were downregulated (Supplemental Figure 4, A and B). Moreover, approximately 30% of nuclear genome-encoded mitochondrial genes displayed altered expression under m^6^A-deficient conditions compared with controls, suggested that m^6^A deficiency may disrupt mitochondrial homeostasis. Methylated RNA immunoprecipitation sequencing (MeRIP-seq) of *Mettl3*^K480R/K480R^ mESCs revealed a global reduction in m^6^A modification (Supplemental Figure 4, C–E), and a m^6^A peak downregulated in K480R mutant was identified within the 3′UTR of mitochondrial transcription factor A (*Tfam*), a master regulator of mitochondrial DNA transcription and maintenance and a known contributor to PD pathogenesis (39–42) (Supplemental Figure 4F). Integrated analysis of *Mettl3*^K480R/K480R^ m^6^A-seq data and RNA-seq data from the *Mettl3*^loxp/loxp^; DAT-Cre mice showed that genes with decreased m^6^A were more likely to show increased mRNA abundance (Supplemental Figure 4G), consistent with the well-established role of m^6^A in promoting mRNA decay for a large subset of transcripts (17). Genes harboring K480R-regulated m^6^A peaks and showing significant differential expression in the SN from *Mettl3*^loxp/loxp^; DAT-Cre mice, were enriched in autophagy, axonogenesis, and synapse-related pathways (Supplemental Figure 4H), processes previously reported to be associated with PD pathogenesis (5, 43, 44). These results suggest that m^6^A may have an effect on multiple pathways related to PD.

**Figure 5:**
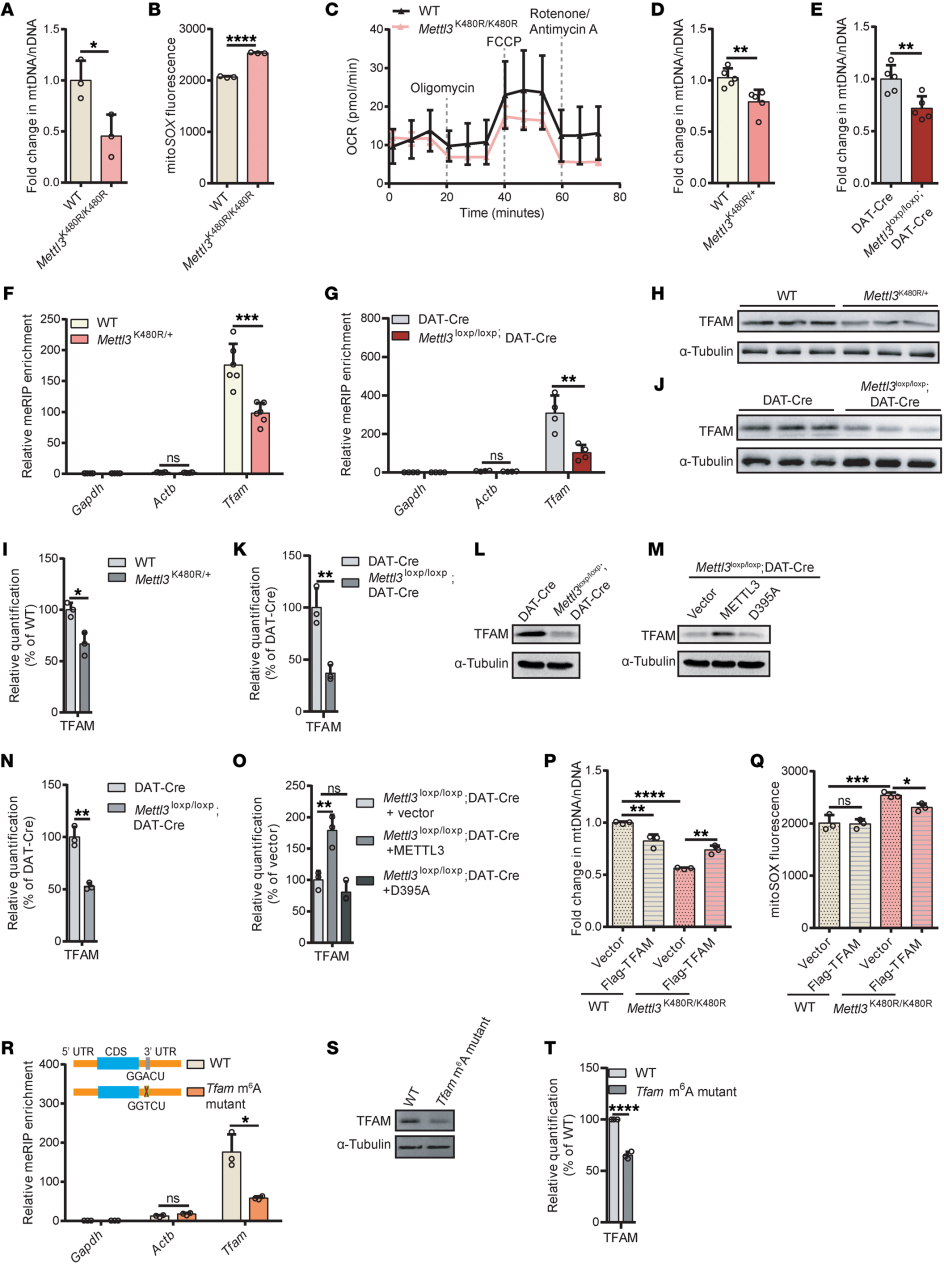
m^6^A deficiency impairs mitochondrial function. (**A**) mtDNA copy numbers in *Mettl3*^K480R/K480R^ mESCs (*n* = 3). (**B**) ROS levels in *Mettl3*^K480R/K480R^ mESCs (*n* = 3). (**C**) Oxygen consumption rate (OCR) traces from *Mettl3*^K480R/K480R^ mESCs (*n* = 4). (**D**) mtDNA copy numbers from WT and *Mettl3*^K480R/+^ mice (*n* = 5). (**E**) mtDNA copy numbers from DAT-Cre and *Mettl3*^loxp/loxp^; DAT-Cre mice (*n* = 5). (**F** and **G**) MeRIP-qPCR analysis of m^6^A peaks on *Tfam* in the SN from 6-month-old WT and *Mettl3*^K480R/+^ mice (**F**, *n* = 6) and DAT-Cre and *Mettl3*^loxp/loxp^; DAT-Cre mice (**G**, *n* = 4). MeRIP enrichment (IP/Input) was normalized to *Gapdh*. (**H**–**K**) TFAM protein levels in the SN from WT and *Mettl3*^K480R/+^ mice (**H**), with quantification (**I**), and from DAT-Cre and *Mettl3*^loxp/loxp^; DAT-Cre mice (**J**), with quantification (**K**). (**L** and **M**) Immunoblot showing TFAM protein levels in primary DA neurons derived from fetal SN from DAT-Cre and *Mettl3*^loxp/loxp^; DAT-Cre mice (**L**) and following overexpression of vector, METTL3, or METTL3 D395A (**M**). (**N** and **O**) Quantification corresponding to **L** and **M** (*n* = 3). (**P** and **Q**) mtDNA copy numbers (**P**) and ROS levels (**Q**) in WT and *Mettl3*^K480R/K480R^ mESCs overexpressing TFAM (*n* = 3). (**R**) MeRIP-qPCR analysis of m^6^A peaks on *Tfam* in WT and *Tfam* m^6^A mutant cells (*n* = 3). MeRIP enrichment (IP/input) was normalized to *Gapdh*. (**S** and **T**) TFAM levels (**S**) with quantification (**T**; *n* = 3) in WT and *Tfam* m^6^A mutant cells. Data are shown as the mean ± SD; 2-tailed Student’s *t* test (**A**, **B**, **D**–**G**, **I**, **K**, **N**, **R**, and **T**), 1-way ANOVA followed by Holm-Šidák multiple-comparisons test (**O**), and 2-way ANOVA followed by Holm-Šidák multiple-comparisons test (**P** and **Q**). *P < 0.05, **P < 0.01, ***P < 0.001, ****P < 0.0001.

Given the established role of mitochondrial dysfunction in PD pathogenesis (5–7), we investigated whether m^6^A deficiency contributes to PD by impairing mitochondria. Indeed, we observed a notable reduction in mitochondrial DNA (mtDNA) copy number in *Mettl3*^K480R/K480R^ mutant cells (Figure 5A) along with elevated mtROS levels (Figure 5B). Oxygen consumption rate measurements revealed impaired basal respiration and maximal OCR in mutant cells (Figure 5C). These findings were corroborated by the reduction of mtDNA copy number in the SN of *Mettl3*^K480R/+^ mice and *Mettl3*^loxp/loxp^; DAT-Cre mice (Figure 5, D and E). These mitochondrial dysfunction phenotypes are consistent with TFAM deficiency. Furthermore, the reduction of *Tfam* m^6^A peaks was confirmed by MeRIP-qPCR in the *Mettl3*^K480R/K480R^ cells (Supplemental Figure 4I) and in the SN of *Mettl3*^K480R/+^ (Figure 5F) and *Mettl3*^loxp/loxp^; DAT-Cre mice (Figure 5G), which correlated with decreased *Tfam* expression (Figure 5, H–K, and Supplemental Figure 4, J–N).

Then, we overexpressed METTL3 in *Mettl3*^K480R/K480R^ cells and found that it rescued TFAM expression (Supplemental Figure 5, A and B) and mtDNA copy number (Supplemental Figure 5C). Additionally, STM2457 treatment in SH-SY5Y–derived DA neuron–like cells reduced TFAM protein levels, mtDNA copy number, and elevated ROS production. These effects could be rescued by WT METTL3, not D395A mutant (Supplemental Figure 5, D–K). Meanwhile, in DA neurons from *Mettl3*^loxp/loxp^; DAT-Cre mice, overexpression of WT METTL3 rescued the reductions of TFAM protein, whereas the D395A mutant failed to do so (Figure 5, L–O). These results indicate that the mitochondrial dysfunction is mediated by m^6^A deficiency. Finally, overexpressed TFAM in *Mettl3*^K480R/K480R^ cells rescued the mtDNA copy number and mtROS levels (Figure 5, P and Q, and Supplemental Figure 5, L and M), demonstrating that TFAM is a key downstream effector.

Further investigation into the role of m^6^A in *Tfam* mRNA using human and mouse GLORI-seq data (33, 45) identified a conserved m^6^A modification site in the 3′UTR of *Tfam* mRNA (Supplemental Figure 6A). We subsequently generated an mESC cell line in which the m^6^A-modified residue A was mutated to T (Supplemental Figure 6, B–D). MeRIP-qPCR analysis revealed a reduction in m^6^A levels on *Tfam* mRNA (Figure 5R), which was accompanied by decreased *Tfam* expression (Figure 5, S and T, and Supplemental Figure 6E). Moreover, in response to the TFAM reduction, the mtDNA copy number decreased and ROS increased (Supplemental Figure 6, F and G). Overexpression of TFAM in this mutant rescued the mtDNA (Supplemental Figure 6, H–J), while overexpression of METTL3 failed to do so (Supplemental Figure 6, K–M). Correlation analysis between m^6^A and TFAM using gene expression data of human SN tissues from the GTEx project (46) showed a positive correlation between m^6^A/METTL3 and TFAM (Supplemental Figure 6, N and O).

Subsequently, we investigated the mechanism by which m^6^A regulated TFAM expression. In *Mettl3*^loxp/loxp^; DAT-Cre mice and *Mettl3*^K480R/K480R^ mESC cells, the protein level of TFAM was consistently decreased, while the mRNA was not decreased in Mettl3^K480R/+^ mice (Figure 5, H–K, and Supplemental Figure 4, J–N). These observations led us to hypothesize that m^6^A may regulate TFAM at the level of protein expression. To test this, we conducted a translating ribosome affinity purification (TRAP) assay and found that the translation efficiency of TFAM was decreased in *Mettl3*^K480R/K480R^ mESCs (Supplemental Figure 6P). To determine whether the cis-acting m^6^A motif is sufficient to enhance TFAM protein expression, we generated GFP reporters fused to the *Tfam* 3′UTR fragment containing either the native m^6^A motif or a mutated m^6^A motif. While the mRNA level of these 2 reporters was comparable, the reporter containing the native m^6^A motif exhibited higher GFP protein than the mutant reporter (Supplemental Figure 6, Q–S). Finally, to identify the m^6^A reader involved, we focused on YTHDF1, a well-characterized m^6^A reader that has been reported to promote translation of m^6^A-modified transcripts (21). Knockdown of YTHDF1 in mESCs led to a significant reduction in TFAM protein abundance without affecting *Tfam* mRNA levels (Supplemental Figure 6, T–W). Together, these results demonstrate that m^6^A regulates TFAM through a translation-biased mechanism.

### Mitochondrial dysfunction reciprocally contributes to METTL3 reduction and m^6^A deficiency.

**Figure 6:**
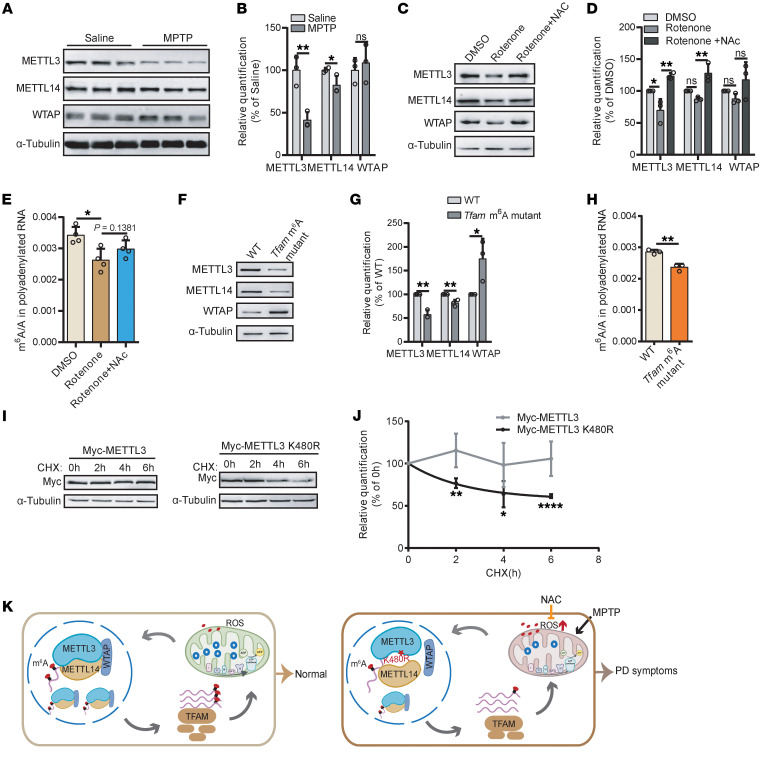
Mitochondrial dysfunction reciprocally contributes to METTL3 reduction and m^6^A deficiency. (**A**) Immunoblot showing the protein levels of METTL3, METTL14, and WTAP in the SN of MPTP-induced PD mice. (**B**) Protein quantification corresponding to **A** (*n* = 3). (**C**) Immunoblot showing the protein levels of METTL3, METTL14, and WTAP in WT mESCs treated with rotenone or rotenone plus NAC for 24 hours. (**D**) Quantification corresponding to **C** (*n* = 3 independent biological samples per group). (**E**) LC-MS/MS quantification of mRNA m^6^A abundance in WT mESCs treated with rotenone or rotenone plus NAC for 24 hours (*n* = 4). (**F**) Immunoblot showing the protein levels of METTL3, METTL14, and WTAP in WT and *Tfam* m^6^A mutant cells. (**G**) Quantification corresponding to **F** (*n* = 3 independent biological samples per group). (**H**) LC-MS/MS quantification of mRNA m^6^A abundance in WT and *Tfam* m^6^A mutant cells (*n* = 3). (**I**) Immunoblot showing the protein levels in HEK293T cells transfected Myc-METTL3 or Myc-METTL3 K480R following rotenone and a time-course cycloheximide treatment. (**J**) Quantification corresponding to **I** (*n* = 3 independent biological samples per group). (**K**) Proposed model illustrating pathogenic crosstalk between m^6^A deficiency and mitochondrial dysfunction that may drive PD pathogenesis. Data are shown as the mean ± SD.; 2-tailed Student’s *t* test (**B**, **G**, **H**, and **J**) and 1-way ANOVA followed by Holm-Šidák multiple-comparisons test (**D** and **E**). *P < 0.05, **P < 0.01, ****P < 0.0001.

Given that treatment with MPTP, a mitochondrial complex I inhibitor, significantly decreased m^6^A levels and METTL3 protein levels in the SN (Figure 1A and Figure 6, A and B), we hypothesized that mitochondrial dysfunction might reciprocally contribute to METTL3 reduction and m^6^A deficiency. To test this hypothesis, we exposed mESC and SH-SY5Y cells to rotenone, another mitochondrial complex I inhibitor, for 24 hours. This treatment similarly reduced the METTL3 protein level (Figure 6, C and D, and Supplemental Figure 7, A and B) and mRNA m^6^A levels (Figure 6E). Given the recent finding that ROS promotes METTL3 degradation (47), we treated cells with a ROS scavenger, N-acetylcysteine (NAC), and found that the effects of rotenone were partially rescued (Figure 6, C–E, and Supplemental Figure 7, A and B), suggesting that mitochondrial ROS may partially mediate the decrease in METTL3 and m^6^A. In the *Tfam* m^6^A motif mutant cells, which exhibit mitochondrial dysfunction (Supplemental Figure 6, F and G), we likewise observed obvious decreases in METTL3 protein level and m^6^A levels (Figure 6, F–H), further supporting the role of mitochondrial dysfunction in m^6^A regulation.

Next, we investigate the reduction of METTL3 protein levels in *Mettl3*^K480R/+^ mutant mice (Figure 2, A–D). Mitochondrial ROS has been shown to reduce METTL3 protein stability (Figure 6, C and D). In addition, aging is associated with elevated ROS levels (48), and DA neurons in SN have a high oxidative burden (49). We therefore reasoned that the K480R mutation might be more vulnerable to mitochondrial ROS. Consistent with this, the K480R mutation weakens the interaction between METTL3 and METTL14, which may lead to an increased pool of unbound METTL3 (Figure 1, J and K). To directly compare the relative stability of METTL3 WT and K480R under oxidative stress conditions, we transfected them into HEK293T cells, blocked protein synthesis with cycloheximide, and induced mitochondrial ROS using rotenone. WT remained stable over a 6-hour period, whereas K480R displayed a marked reduction, suggesting increased instability under ROS exposure (Figure 6, I and J). Together, these results indicate that the METTL3 K480R is more susceptible to ROS-mediated degradation, supporting a model in which oxidative stress contributes to the progressive reduction of METTL3 K480R.

Collectively, these data indicate that m^6^A deficiency disrupts mitochondrial function by downregulating TFAM, while mitochondrial dysfunction reciprocally diminishes m^6^A modification. This interaction suggests a pathogenic feed forward loop that may drive PD pathogenesis (Figure 6K).

### SAMe replenishment mitigates parkinsonism in mouse models.

**Figure 7:**
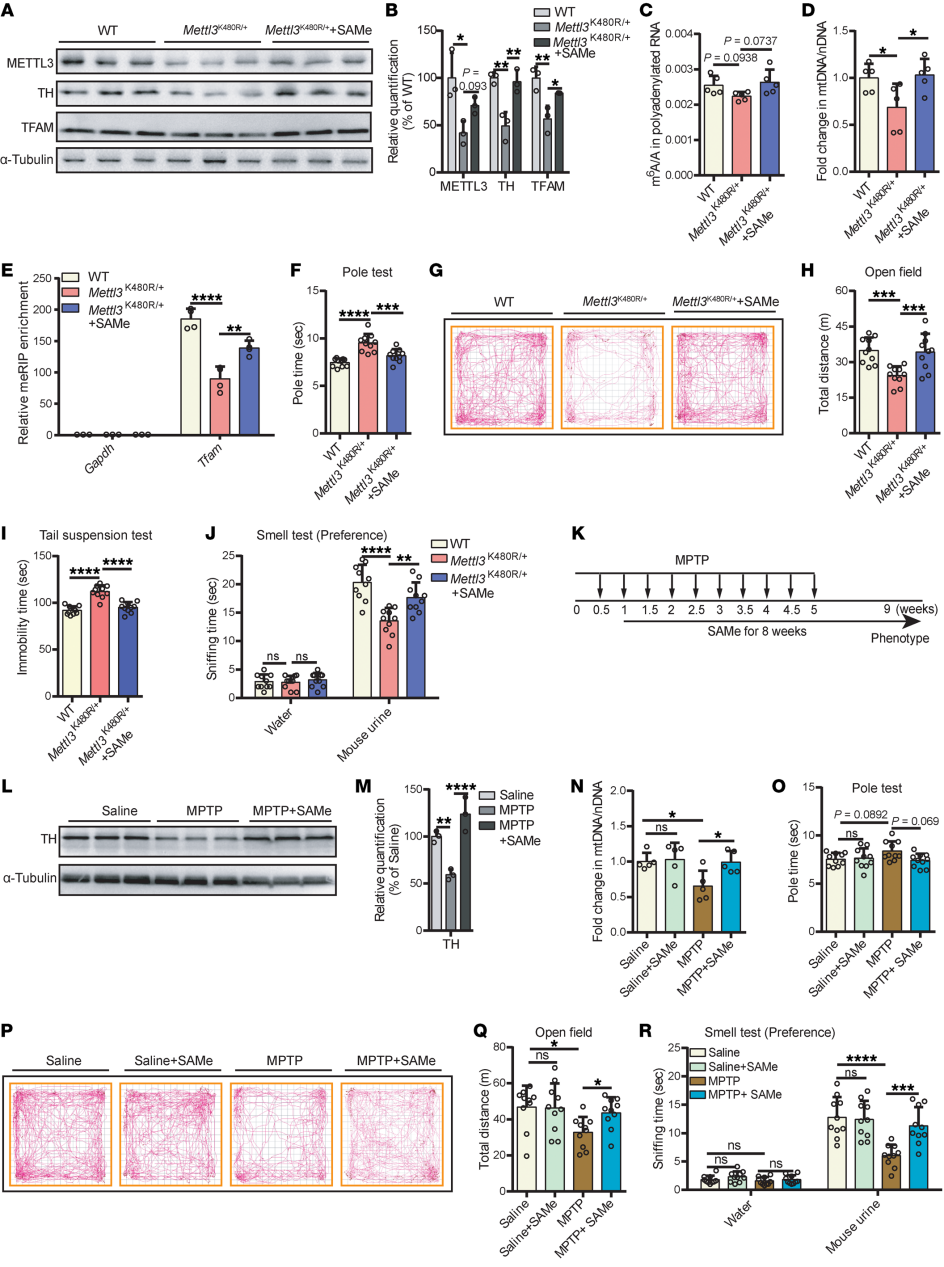
SAMe replenishment mitigates parkinsonism in mouse models. (**A**) Immunoblot showing the protein levels of METTL3, TH, and TFAM in the SN from 6-month-old WT or *Mettl3*^K480R/+^ mice treated with SAMe for 2 months. (**B**) Protein quantification corresponding to **A** (*n* = 3). (**C**) LC-MS/MS quantification of mRNA m^6^A abundance in the SN (*n* = 5). (**D**) mtDNA copy numbers in the SN (*n* = 5). (**E**) MeRIP-qPCR analysis of m^6^A peaks for *Tfam* in the SN (*n* = 4). MeRIP enrichment (IP/input) was normalized to *Gapdh*. (**F**–**H**) Pole test (**F**), open-field traces (**G**), and total distance traveled in 30-minute open-field test (**H**) (*n* = 10). (**I** and **J**) Tail suspension test (**I**) and olfactory preference test (**J**) (*n* = 10). (**K**) Schematic of SAMe supplementation in the MPTP-induced PD model. (**L**) Immunoblot showing the protein levels of TH in the SN from MPTP-induced PD model mice treated with SAMe. (**M**) Quantification corresponding to **L** (*n* = 3). (**N**) mtDNA copy numbers in the SN from MPTP-induced PD model mice treated with SAMe (*n* = 5). (**O**–**R**) Pole test (**O**), open-field traces (**P**), total distance traveled in 30-minute assessment (**Q**), and olfactory preference test (**R**) (*n* = 10). Data are shown as the mean ± SD; 1-way ANOVA followed by Holm-Šidák multiple-comparisons test (**B**–**F**, **H**–**J**, and **M**) and 2-way ANOVA followed by Holm-Šidák multiple-comparisons test (**N**, **O**, **Q**, and **R**). *P < 0.05, **P < 0.01, ***P < 0.001, ****P < 0.0001.

SAMe, commonly used as a clinical drug and dietary supplement for the treatment of liver disease and major depressive disorder (50, 51), is a methyl group donor for m^6^A modification (52, 53) and can facilitate m^6^A methylation (54, 55). We sought to determine whether SAMe supplementation could mitigate the parkinsonism in m^6^A-deficient PD models. We supplemented the diets of *Mettl3*^K480R/+^ mice with SAMe for 2 months starting at 4 months of age. This treatment partially restored METTL3 protein levels, m^6^A levels, and mtDNA copy number in the SN (Figure 7, A–D). SAMe treatment also partially restored TH protein levels and TFAM expression and *Tfam* m^6^A levels in the SN of *Mettl3*^K480R/+^ mice (Figure 7, A, B, D, and E, and Supplemental Figure 8A). While in *Tfam* m^6^A mutant cells, SAMe treatment could not restore TFAM protein levels (Supplemental Figure 8, B and C). We observed significant improvements in the pole test, open-field test, tail suspension test, and olfactory test following treatment as well (Figure 7, F–J). We also investigated whether SAMe supplementation could alleviate the PD phenotype in the MPTP-induced PD model, which similarly displayed an m^6^A deficiency (Figure 1A). Mice were administered MPTP (150 mg/kg total) twice weekly for 5 weeks to establish the PD model (56). Starting 1 week after the first MPTP injection, mice were fed SAMe-supplemented diets for 8 weeks (Figure 7K). The TH levels and mtDNA copy number were partially restored in the SN of SAMe-treated mice (Figure 7, L–N). In addition, SAMe supplementation partially rescued the deficits in motor activity and olfactory function seen in MPTP-treated animals (Figure 7, O–R). These results demonstrate that SAMe supplementation may interrupt the m^6^A-mitochondria dysfunction cycle and holds therapeutic potential in mitigating PD symptoms.

## Discussion

Recent studies have increasingly highlighted the pivotal role of m^6^A dysregulation in neurodegenerative diseases such as Alzheimer’s disease (25, 57), C9ORF72-ALS/FTD (26), and Huntington’s disease (58). Our findings indicate that m^6^A deficiency can induce progressive parkinsonian symptoms, suggesting a general involvement of m^6^A modifications across various neurodegenerative disorders. Moreover, our study revealed an interplay between m^6^A and mitochondrial function, with this regulation potentially exacerbating aberrant effects once abnormalities exceed a certain threshold. This mechanism may contribute to the characteristic progressive decline observed in neurodegenerative diseases. Additionally, m^6^A modifications were found to regulate key genes involved in organelles and functional complexes related to neurodegenerative diseases such as autophagosomes (59) and stress granules (60). Further research is warranted to explore whether similar interplays exist between m^6^A and other organelles or complexes and to elucidate their potential functions in neurodegenerative disease progression.

Animal models are valuable for studying disease mechanisms, and the creation of models based on specific pathogenic mechanisms may expedite the development of targeted therapies (61). Although several PD models have been created using toxins, drugs, or transgenic methods (61–63), there has been no RNA modification–related PD model reported to date. The present study establishes a progressive PD model based on m^6^A deficiency, which may provide a useful preclinical platform for investigating stage-dependent pathogenic mechanisms and for evaluating therapeutic interventions across disease progression. Furthermore, our data indicate that supplementation with SAMe alleviated PD symptoms in mouse models. Additionally, entacapone, currently employed in the treatment of PD (64), has recently been identified as an inhibitor of the m^6^A demethylase FTO (65). These findings support the idea that enhancing m^6^A levels could be a promising therapeutic intervention in PD. Our model may also facilitate future efforts to identify METTL3 activators and explore m^6^A-targeted interventions.

Ageing is the greatest risk factor for the development of PD. The accumulating effects of mitochondrial damage, oxidative stress, and epigenetic alterations may accelerate DA neuron dysfunction and death (66). In our study, METTL3 expression decreased with age in the SN of *Mettl3*^K480R/+^ mice, accompanied by a progressive reduction in m^6^A modification. Age-associated increases in mitochondrial dysfunction and ROS may contribute to METTL3 reduction and m^6^A deficiency, which in turn contribute to PD pathogenesis. SAMe supplementation may help to mitigate this pathogenic loop linking METTL3 reduction, m^6^A deficiency, and mitochondrial ROS by partially restoring m^6^A methylation. Consistent with this model, METTL3 protein levels were increased in the SN of *Mettl3*^K480R/+^ mice following SAMe treatment. Additional mechanisms may also contribute to METTL3 reduction and remain to be explored.

Our study found abnormal methyltransferase and demethylase expression in the DA neurons of patients with PD and demonstrated that a METTL3 K480R mutation observed in a patient with PD led to m^6^A deficiency and neurodegeneration in mice. The scarcity of human PD brain samples limited our ability to directly analyze m^6^A alterations in PD neurons. Future studies utilizing single-cell m^6^A sequencing technologies (67, 68) may enable the identification of neuron-specific m^6^A alterations in patients with PD. Moreover, studies with larger, diverse cohorts are needed to assess the contribution of METTL3 and K480R at the population level and to further clarify their relevance to PD pathogenesis.

Variable expression of several mtDNA-encoded genes was observed in *Mettl3*^K480R/K480R^ mESCs and *Mettl3*^loxp/loxp^; DAT-Cre mouse SN cells. This variability may reflect technical challenges in accurately quantifying certain mitochondrial transcripts (e.g., *Atp8*, *Atp6*, *Cox3*, *Nd3*, and *Nd4l*), which are prone to secondary alignment artifacts, as well as biological factors, including tissue-specific mitochondrial demand and posttranscriptional regulation (69). Model-specific differences may further influence the magnitude of expression changes, as the knockin and conditional knockout models represent different modes and extents of METTL3 perturbation. Furthermore, only a subset of nuclear-encoded mitochondrial genes showed reduced m^6^A, suggesting that additional indirect regulatory mechanisms may be involved. We speculate that m^6^A loss decreases TFAM expression, thereby impairing mitochondrial biogenesis and function. These effects may disrupt mitonuclear coordination and engage stress signaling pathways, resulting in secondary changes in nuclear mitochondrial gene expression that reinforce mitochondrial insufficiency.

## Methods

### Sex as a biological variable.

Our study incorporated whole-exome sequencing data from humans with PD, including individuals of both sexes in the genetic analyses. Sex was not evaluated as an independent variable in the human genetic analyses. For animal experiments, only male mice were used to minimize variability associated with hormonal fluctuations or pregnancy-related factors in females and maintain consistency with the majority of prior studies of PD models. It is unknown whether the findings are relevant for female mice.

### Mouse strains.

METTL3 p.K480R (AAG>CGC) knockin mice were generated by Cyagen Biosciences using the congenic C57BL/6 strain (Supplemental Figure 2A). The sgRNA (5′-TCACTGGTTAAACCACGGGA-3′; PAM: AGG) and a donor oligonucleotide (5′-ACTAATCAGCTGCAGCGCATCATTAGGACGGGCCGGACGGGTCACTGGTTAAACCACGGGCGCGAACACTGCTTGGTGAGGAACAGGAGGAAGAGGGGGAGCTGCTTAGGAGACTGAAGGCTG-3′) were coinjected with Cas9 mRNA into zygotes. Founder animals were screened by PCR genotyping, and precise editing was verified by Sanger sequencing. Primer sequences are listed in Supplemental Table 1. The *Mettl3* flox mouse strain and DAT-Cre transgenic mice were obtained from Cyagen Biosciences. All mice were housed in the animal care facility at Southern Medical University and maintained at a controlled temperature of 22°C–24°C, with a 12-hour light/12-hour dark cycle.

### Cell line generation.

*Mettl3*^K480R^ and *Tfam* m^6^A mutant knockin cell lines were generated using CRISPR-Cas9. sgRNAs were designed via the CRISPR-ERA (70) and cloned into the pXPR_001. For *Mettl3*^K480R^, the sgRNA was 5′-GGTTTAACCAGTGACCCGTC-3′ (PAM: CGG). A single-stranded donor oligonucleotide introduced the K480R substitution (c.1439A>G) together with 3 synonymous mutations (GGC at aa 470 to GGA, ACG at aa 472 to ACA, and GGT at aa 473 to GGA) to facilitate restriction enzyme–based genotyping. Correctly edited clones were identified by PCR and confirmed by Sanger sequencing. For the *Tfam* m^6^A mutant, a m^6^A motif within the *Tfam* 3′UTR was mutated (*c.131A>T) using the sgRNA (5′-CAAACTAGAACGGATAAAGG-3′; PAM: TGG) and the donor oligonucleotide (5′-CAAACTAGAACGGATAAAGGTGGTTAACCTTTGACATTCAGATCATTTTTCTGTAGCCATGGtCTTTCTGTTAATACTTTGAGCCTTGACAGAAGATGA-3′). Edited clones were validated by PCR and Sanger sequencing.

METTL3/YTHDF1 knockdown cells were generated by lentiviral shRNA infection and puromycin selection, and the knockdown efficiency was verified by qPCR or Western blots.

For overexpression experiments, mouse *Tfam*, *Mettl3*, or *Mettl3* D395A CDS were cloned into pLVX-FLAG-tagged vectors. GFP reporters containing WT or mutant *Tfam* 3′UTR fragments were generated in the same backbone. For Co-IP assay, human METTL3 or METTL3 K480R CDS was cloned into pCDNA4.0-Tre-SBP-FLAG-S-protein-tagged (SFB) vector. Constructs were transfected into mESCs or HEK293T cells followed by antibiotic selection.

### Western blots.

Western blots were performed as described previously (23). For the co-IP assay, SFB tagged vector was transfected in HEK293T cells, and the immunoprecipitation was carried out as described previously (23). The antibodies used were as follows: METTL3 (A19079, ABclonal, 1:1,000), METTL14 (80790-1-RR, Proteintech, 1:2,000), WTAP (60188-1-Ig, Proteintech, 1:2,000), H3 (17168-1-AP, Proteintech, 1:5,000), GAPDH (60004-1-Ig, Proteintech, 1:3,000), TH (25859-1-AP, Proteintech, 1:5,000), p-α-synuclein (S129) (23706S, Cell Signaling, 1:1,000), TFAM (A3173, ABclonal, 1:1,200), and α-tubulin (66031-1-Ig, Proteintech, 1:3,000).

### LC-MS/MS analysis.

Liquid chromatography–tandem mass spectrometry (LC-MS/MS) was performed as described previously (32). Purified mRNA (20 ng) was digested by 0.5 U nuclease P1 (Sigma) and 0.5 U of CIAP (Takara), then diluted to 1 ng/μL, filtered, and injected into an Agilent Poroshell 120 column. This was coupled online to an AB SCIEX Triple Quad 5500 LC mass spectrometer (Applied Biosystems) operating in positive electrospray ionization mode. Quantitation was based on a standard curve of nucleosides, and m^6^A/A ratios were calculated by concentration.

### Immunohistochemistry.

Mice were anesthetized and perfused with prechilled PBS followed by 4% paraformaldehyde. Brains were dissected, postfixed in 4% paraformaldehyde at 4°C for 2 hours, transferred to 30% sucrose solution at 4°C until sinking, and stored in 0.02% sodium azide at 4°C. After washing with PBS, tissues were processed using an automated tissue processor (Leica ASP300) and embedded in paraffin (Leica EG1150H). For immunofluorescence, 10 μm paraffin sections on glass slides were heated at 60°C for 30 minutes, deparaffinized in xylene, and rehydrated through decreasing alcohol concentrations. Slides were treated with methanol/hydrogen peroxide (0.3%) solution for 10 minutes at room temperature to block endogenous peroxidase. For antigen retrieval, slides were immersed in boiling 0.1 M citrate buffer (pH 6.0) and cooked at maximum pressure for 10 minutes. Slides were then blocked in PBTA for 1 hour at room temperature and processed with primary and secondary antibodies. The primary antibodies used were TH (AB152, Millipore, 1:1,000) and p-α-synuclein (S129) (23706S, Cell Signaling, 1:200). Images were acquired using a Zeiss LSM 880 confocal microscope and analyzed with ImageJ (NIH).

### mtDNA copy number.

mtDNA copy number was performed as described previously (71). Total genomic DNA was extracted, and the mtDNA/nDNA ratio was measured by qPCR using primers specific for COX2 (mtDNA) and normalized to Hk2 (nDNA) to control for input DNA amount.

### snRNA-seq of mouse SN.

Single-nucleus suspensions were prepared from dissected mouse SN tissues as previously described (72). Briefly, SN tissue was rapidly processed on ice. Nuclei were purified by wash/filtration steps, assessed for integrity under microscopy, and counted prior to library preparation. To minimize ambient RNA contamination, nuclei were handled at low temperature, and only high-quality preparations were loaded for droplet encapsulation. Libraries were generated using a droplet-based single-nucleus platform (Seekone DD platform) and sequenced following the manufacturer’s guidelines.

### L-DOPA treatment.

L-DOPA (TargetMol) and benserazide hydrochloride (Aladdin) were freshly dissolved in saline. The final injection volume was 5 mL/kg. L-DOPA (12 mg/kg) and benserazide hydrochloride (6.25 mg/kg), or a vehicle solution (saline), were injected intraperitoneally to mice 1 hour before the start of the behavioral tests (7).

### Generation of the SAMe administration mouse model.

SAMe (Coolaber) was incorporated into the diet pellets at a final concentration of 0.1 g/kg to achieve a daily SAMe administration of 400 μg per mouse, based on an estimated food consumption of approximately 4 g/d (73). No differences were observed between the test group and the control group.

### MeRIP-seq and qPCR.

MeRIP-seq was carried out as previously reported (32, 74). Briefly, 30 μg of 100- to 300-nucleotide-long fragmented RNA was incubated with 1.5 μg anti-m^6^A antibody (ab151230, abcam). This mixture was then bound to Dynabeads protein G (Invitrogen). After stringent washing, the bound RNA was eluted by competition with N6-methyladenosine (Selleck) and extracted for downstream analysis. The immunoprecipitated RNAs were reverse transcribed and analyzed by qPCR. Ratios of immunoprecipitated to input for peaks were calculated and normalized. Libraries were generated with a SMARTer Stranded Total RNA-Seq Kit v.2-Pico Input Mammalian (Takara) following the manufacturer’s instructions. Sequencing was carried out on a DNBSEQ-T7 platform.

### Analysis of meRIP-seq data.

MeRIP-seq data were analysis as previously reported (32). Peaks reaching the cutoff (FC ≥ 1.5 or ≤ 0.67, FDR < 0.05 by DESeq2, v.1.38.3) (75) were defined as up- or downregulated m^6^A peaks.

### Analysis of RNA-seq data.

Read mapping and processing were performed using the MeRIP–seq input sample. FeatureCounts (v.2.0.6) (76) was used to calculate the number of reads mapped to each gene. DESeq2 was used for differential expression analysis.

### snRNA-seq data analysis.

Raw gene expression matrices were analyzed using Seurat v4.3.0.1 (77). Cells expressing fewer than 200 genes were excluded. Data were normalized using the LogNormalize method, with a scaling factor of 10,000 reads per cell. The top 2,000 highly variable genes were selected using Find Variable Features function with the “vst” method. PCA was performed, and cells were clustered based on the top 30 principal components with a resolution parameter of 0.3 and visualized using uniform manifold approximation and projection (UMAP). Cell types were annotated by comparing the marker gene expression in cluster with a published mouse midbrain scRNA-seq reference dataset, classifying cells into 7 major types. Differential gene expression was assessed using the Wilcoxon’s rank-sum test with Benjamini-Hochberg correction.

### Statistics.

Graph plots and *P* values were generated using GraphPad Prism 6 software. ImageJ (NIH) was used to quantify the images. Data were analyzed using 2-tailed Student’s *t* test, 1-way ANOVA, 2-way ANOVA, Fisher’s exact test, or Wilcoxon’s rank-sum test, as appropriate. *P* values of less than 0.05 were considered significant.

### Study approval.

Patients with PD between 2017 and 2020 from Guangzhou KingMed Diagnostics were retrospectively retrieved, and their test results and histological follow-up results were collected and analyzed. All patients provided written informed consent. Approval was obtained from the ethics committee of Guangzhou KingMed Diagnostics (reference no. 2019014). UK Biobank (UKB, Stockport, UK) has ethical approval from the North West Multi-Centre Research Ethics Committee as a Research Tissue Bank for the collection and storage of biological samples and data and their use for approved research. This research was conducted under UKB application number 81596.

All animal experiments adhered to the NIH’s *Guide for the Care and Use of Laboratory Animals* (National Academies Press, 2011) and were approved by the IACUC of Southern Medical University Experimental Animal Ethics Committee (approval no. L2016164 and L2022158).

### Data availability.

DA neuron snRNA-seq data were downloaded from the NCBI GEO database with accession GSE178265. Human brain meRIP-seq data were downloaded from the Genome Sequence Archive in Beijing Institute of Genomics (BIG) Data Center, Chinese Academy of Sciences, under accession CRA001315 and the NCBI GEO database with accession GSE114150. To protect patient privacy, the whole-exome sequencing data of individuals with PD is not publicly available. Data supporting the results of this study are available from the corresponding authors upon reasonable request. RNA-seq, MeRIP-seq, and snRNA-seq raw data have been deposited in the Genome Sequence Archive under accession CRA022216. Values for all data points in the graphs are reported in the Supporting Data Values file.

## Author contributions

Laixin Xia and SX designed and supervised the project. SL, QR, GM, ZM, ZX, SY, GW, XC, YL, YZ, and JS performed the experiments. HH, ZL, Linjian Xia, JC, BW, and JM conducted the bioinformatics analysis. SX, Laixin Xia, and SL wrote the manuscript with input from all authors. All authors reviewed and approved the final manuscript.

## Conflict of interest

GM and SY are employees of Guangzhou KingMed Diagnostics Group Co. Ltd.

## Funding support

National Key R&D Program of China (2021YFA0805400 to SX).

- National Natural Science Foundation of China (32222016 to SX and 82230053 to Laixin Xia).
- The Guangdong Basic and Applied Basic Research Foundation (2022B1515020107 to SX and 2024A1515013000 to Laixin Xia).

## Supplementary Material

Supplemental data

Unedited blot and gel images

Supporting data values

## Version 1 03/17/2026

In-Press Preview

## Version 2 05/01/2026

Electronic publication
