# Supplementary material for: m6A deficiency induces dopaminergic neurodegeneration and progressive parkinsonism through a pathogenic loop with mitochondria
Source: J Clin Invest. 2026 Mar 17;136(9):e197183. doi: 10.1172/JCI197183 (PMC13132373; doi:10.1172/JCI197183)
Supplement: Supplemental data [file jci-136-197183-s312.pdf]

Contents

Supplemental Methods

Supplemental Figure and figure legends 1-8

Supplemental Tables

Table S1: List of oligonucleotides used in this study

Supplemental Methods

Whole-exome sequencing and variant analysis

Genomic DNA was extracted from the peripheral venous blood using the QIAamp DNA extraction kit (QIAGEN). The extracted DNA was fragmented, purified, followed by PCR amplification and ligation. The whole exome target region was captured using the IDT xGen Exome Research panel (IDT) probe, and the library was sequenced on the NovaSeq 6000 sequencer (Illumina). The raw data were processed using Trimmomatic (v 0.36) (1), mapped to hg19 using BWA (2). And then the genetic variants were identified by GATK (3), and annotated by ANNOVAR (4).

To identify damaging variants in m^6A-related genes, familial patients and sporadic patients with damaging variants in PD disease-causing genes (5) were excluded. 280099 variants in the remaining 90 sporadic patients were processed using a high stringency criterion for the identification of damaging variants: 1. Variants should have an allele frequency $< 1x10^{-4}$ in GnomAD, ExAC, and the 1000 Genomes Project were considered. 2. A REVEL score $\geqslant$ 0.5, indicating a high likelihood of being damaging. 3. Predicted to be damaging by more than half of the 11 software tools used (SIFT, PolyPhen2, LRT, MutationTaster, MutationAssessor, FATHMM, MetaSVM, MetaLR, GERP++, PhyloP, and SiPhy). 4. A conservation score > 2, indicating a high degree of evolutionary conservation.

UKB data analysis

For UKB data analysis, data curation were performed essentially as described (6) (Supplemental Figure 1D). PD cases were identified using an algorithmic definition

incorporating self-reports, hospital records, and death certificates. Variants were filtered using the UKB-provided criteria, including a depth of coverage (DP) ≥ 10 and minor allele frequency (MAF) < 10^{-4}. Variants failing the 90% reading threshold were excluded. Annotation was performed using ANNOVAR.

Cell culture

E14Tg2a mESCs, from National Collection of Authenticated Cell Cultures, were cultured with N2B27 base medium supplemented with 1 mM glutamine (Gibco), 1% nonessential amino acids (Gibco), 2% KOSR (Thermo Fisher), 0.15 mM 1-thioglycerol (Sigma), 1 μM MEK inhibitor PD0325901 (Selleck Chemicals), 3 μM GSK3β inhibitor CHIR99021 (Selleck Chemicals), 100 U mL^{-1} of penicillin–streptomycin (Gibco), 25 μg mL^{-1} of BSA (Sangon), and 1000 U mL^{-1} of ESGRO leukemia inhibitory factor LIF (Millipore) on plates coated with 0.2% gelatin at 37°C with 5% CO_2.

HEK293T cells, from National Collection of Authenticated Cell Cultures, were grown in DMEM (Invitrogen) containing 10% FBS and 1% penicillin–streptomycin. The human neuroblastoma cell line SH-SY5Y, from National Collection of Authenticated Cell Cultures, maintained in DMEM/F-12 supplemented with 10% FBS (ExCell), 100 U mL^{-1} of penicillin–streptomycin, and 1mM glutamine at 37°C with 5% CO_2.

To isolate the primary DA neurons, embryos at embryonic day (E) 13.5-14.5 were rapidly decapitated in serum-free media and the SN tissue was dissected. The tissue was dissociated in 1 mg mL^{-1} papain solution for 10 min at 37 °C. After gentle trituration, cells were plated in 6-well plate and cultured with DMEM/F-12

supplemented with B-27 (Gibco), 1 mM glutamine (Gibco), 1% nonessential amino acids (Gibco), 100 U mL^{-1} of penicillin–streptomycin(Gibco) at 37°C with 5% CO2. DA neurons are mature after around 5-7 days in vitro.

Dopamine level measurement by high-performance liquid chromatography (HPLC)

HPLC was used to analyze the dopamine level in SN samples as previously described (7). Briefly, dissected brain regions were homogenized in a buffer comprising 0.2 M perchloric acid, 0.05% Na2EDTA, 0.1% Na2S2O5. Homogenates were centrifuged at high speed to remove insoluble debris, and the resulting supernatants were filtered through 0.22-μm membranes prior to analysis. Samples were analyzed using a Prelude SPLCTM system coupled to a TSQ Quantiva™ triple quadrupole mass spectrometer (Thermo Fisher Scientific) operating in LC–MS/MS mode. Data acquisition and quantification were performed using TraceFinder™ software according to the manufacturer's instructions. Quantified analytic levels were normalized to total protein content of the corresponding tissue samples.

Seahorse assay for real-time oxygen consumption rate

Seahorse assay for real-time oxygen consumption rate Real-time oxygen consumption rate (OCR) was measured using a Seahorse XFe96 Extracellular Flux Analyzer (Agilent) according to the manufacturer's instructions. mESCs were seeded at a density of 1×10^4 cells per well in Seahorse XF96 Cell Culture Microplates (Agilent) and allowed to adhere overnight. Prior to the assay, cells were washed twice and incubated in Agilent Seahorse XF Base Medium supplemented with 1 mM sodium

pyruvate, 2 mM L-glutamine, and 10 mM D-glucose, with a final volume of 80 μL per well. Cells were then equilibrated for 1 h at 37 °C in a non-CO_2 incubator to allow temperature and pH stabilization. OCR was measured under basal conditions followed by sequential injections of oligomycin (1.5 μM), carbonyl cyanide 4-(trifluoromethoxy) phenylhydrazone (FCCP, 1 μM), and rotenone/antimycin A (0.5 μM), respectively. Following completion of the assay, cells in each well were counted, and OCR values were normalized to cell number. Normalized OCR data were calculated using Wave software (Agilent) by applying well-specific normalization factors.

Behavioral tests

To evaluate behavioral deficits, mice were assessed using several tests at different life stages: the olfactory preference and avoidance test for sensory evaluation, the pole test for, open field test, and tail suspension test for motor deficits and non-motor deficits. The experimenter was blinded to the treatment groups for all behavioral studies to ensure unbiased results. All tests were conducted and recorded between 10:00 and 16:00 during the lights-on cycle. Only male animals were used for the behavioral experiments.

Olfactory preference and avoidance test

The olfactory test was conducted with some modifications as previously described (8). Briefly, mice were placed in a test cage (width 32.5 cm × depth 21.5 cm × height 13 cm). Two pieces of filter paper (2 cm × 2 cm) were positioned at the bottom of the cage (8.5 cm from the long side and 6.5 cm from each short side).

For the olfactory preference test, a test odorant was applied to the filter paper on one side of the cage, and water was applied to the filter paper at the opposite side. The positions of the odorant and water were alternated with each test. The duration of sniffing time of the odorant-treated filter paper was measured.

For the olfactory avoidance test, a bisector was installed in the cage, dividing it into two sections with a piece of filter paper placed in each section. A test odorant was applied to the filter paper on one side, and water was applied to the opposite side's filter paper. The time that mice spent in the area opposite the odorant-treated filter paper was measured.

Pole test

Mice were acclimatized in the behavioral procedure room for 24 h before testing. The pole was involved a 75 cm metal rod with a 9 mm diameter, wrapped in bandage gauze for better grip. Mice were placed at the top of the pole, facing upwards. The total time taken for reach mouse to reach the base of the pole was recorded. Prior to the actual test, mice were trained over two consecutive days, with each training session consisting of three trials. On the test day, each mouse completed three rounds of testing. The duration of each test was recorded， and the average duration was calculated. The maximum cutoff time for each test was 60 seconds, after which the test and recording were stopped.

Tail suspension test

The tail suspension test was performed following the method described by Sangjune Kim et al (9). Mice were individually suspended by the tail in black Plexiglas boxes

(50 cm × 50 cm × 50 cm). The tail was attached to a hook using adhesive tape placed approximately 1 cm from the tip of the tail, with the mouse hanging 5 cm above the floor. The duration of immobility was recorded during a 6-min test period.

Open field test

The open field test was conducted in a rectangular chamber (40 × 40 × 30 cm), constructed from gray polyvinyl chloride. Each mouse was gently placed in the center of the testing chamber and allowed to move freely for a 30-min period. Their movement was monitored and recorded by an automated video tracking system. The paths taken by the mice were digitally recorded and automatically analyzed using EthoVision 11.0 software.

Translating ribosome affinity purification (TRAP)

TRAP was performed as previously described(10), with minor modifications. Briefly, mESCs were treated with 100 μg mL^{-1} CHX for 10 min to arrest translating ribosomes. Cells were then washed three times with ice-cold PBS containing 100 μg mL^{-1} CHX.

Cells were collected and lysed for 10 min on ice in lysis buffer containing 20 mM HEPES–KOH (pH 7.3), 150 mM KCl, 5 mM $MgCl_2$, and 1% NP-40, supplemented with 100 μg mL^{-1} CHX, RNase inhibitor, and protease inhibitor cocktail. Then the lysates were centrifuged and the supernatants were collected. In total, 5 μg of anti-RPL10A antibody (Proteintech, 16681-1-AP) was added to the cleared lysates and incubated for 2 h at 4 °C with gentle rotation. Immune complexes were then captured to Dynabeads protein G (Invitrogen). After washing, beads were incubated in

200 μl buffer containing 0.2 mg/ml Proteinase K at 56 °C for 20 min. The supernatant was collected, and the RNA was extracted by the TRNZOL Reagent (Tiangen). Then RT-qPCR was performed.

Supplemental Figure and figure legends

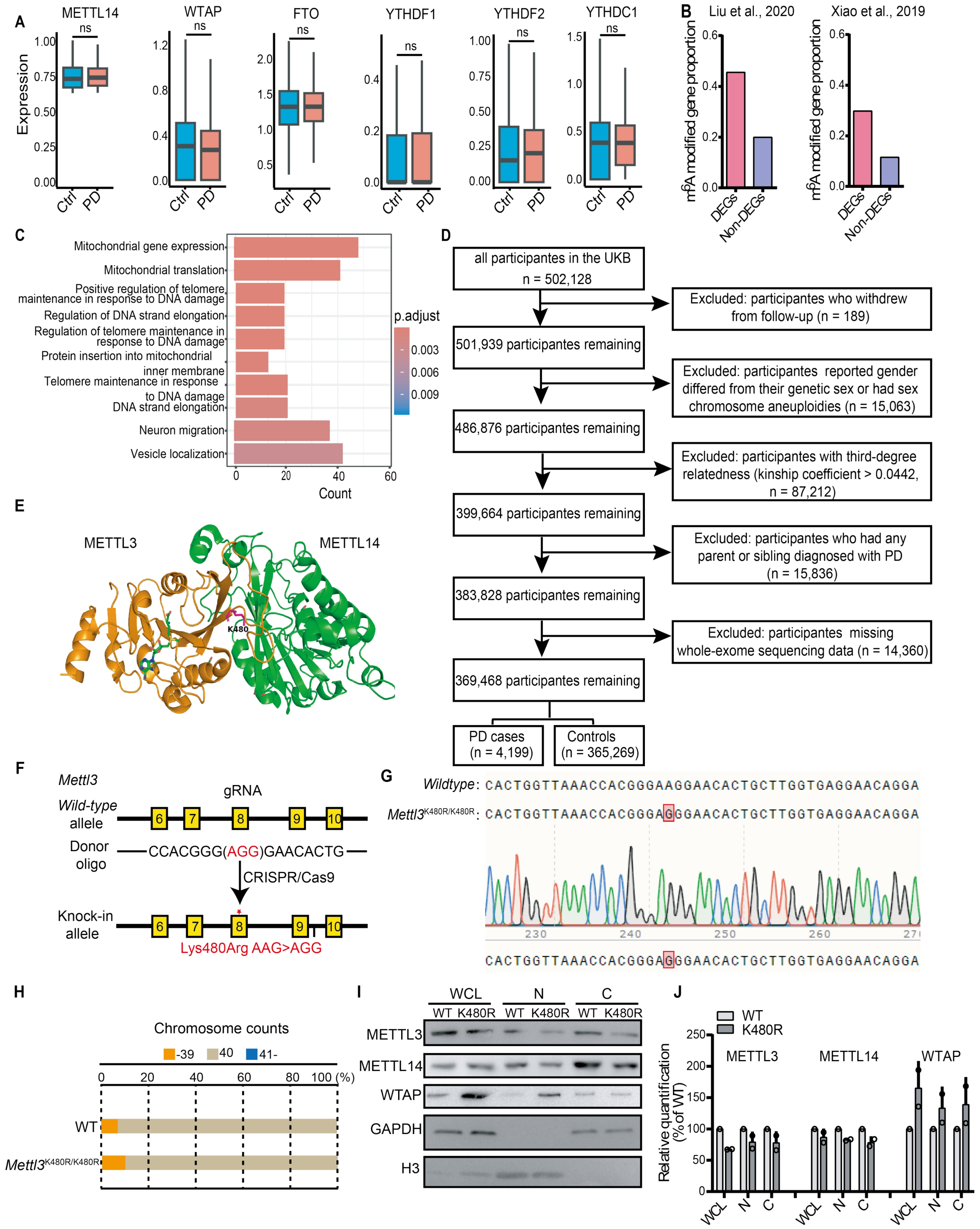

Supplemental Figure 1. m^{6}A modification deficiency is associated with PD. A. Box plots showing normalized expression levels of m^{6}A writers, erasers, and readers in DA neurons of controls ($n = 15458$ from 8 individuals) and PD patients ($n = 2430$ from 7 individuals); Wilcoxon rank-sum test. **B.** m^{6}A modified gene proportion of DEGs and non-DEGs in DA neurons of PD patients. Human brain meRIP-seq data were taken from Liu et al., 2020 (left) and Xiao et al., 2019 (right). **C.** GO enrichment analysis of the m^{6}A-modified genes downregulated in DA neurons of PD patients. **D.** Flow diagram of participant inclusion of UKB. **E.** Crystal structure ribbon diagram

indicating the position of METTL3 K480 in the METTL3 (orange) and METTL14 (green) heterodimer. **F.** Schematic representation of sgRNA and donor oligo used to generate METTL3 K480R knock-in mESC cell line using CRISPR-Cas9. The K480R (AAG to AGG) point mutation was introduced into exon 8. **G.** Verification of K480R mESC clones by Sanger sequencing. **H.** Karyotypes represented by the percentages of the indicated chromosome numbers in WT or *Mettl3*$^{K480R/K480R}$ mESCs. **I.** Western blot showing the subcellular location of METTL3, METTL14, and WTAP proteins in WT and *Mettl3*$^{K480R/K480R}$ mESCs. WCL: whole cell lysate, C: cytoplasmic fraction, N: nuclear fraction. **J.** Protein quantification corresponding to I (n = 2 independent biological samples per group).

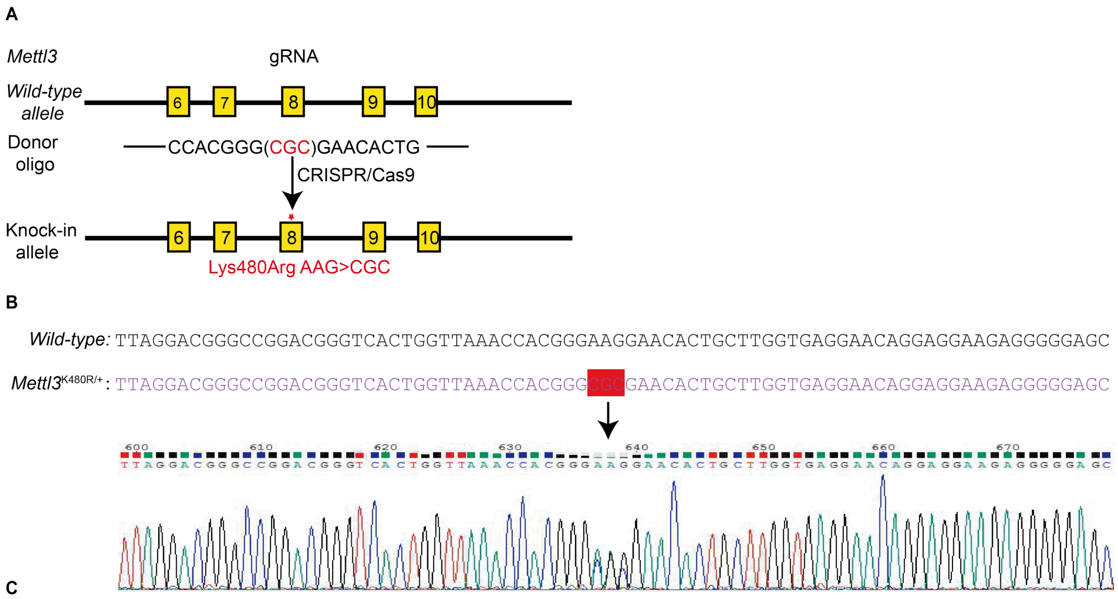

C

Mettl3$^{K480R/+}$ X *Mettl3*$^{K480R/+}$

	Mettl3$^{+/+}$	*Mettl3*$^{K480R/+}$	*Mettl3*$^{K480R/K480R}$
Male	32	67	0
Female	26	50	0
Total	58	117	0

Supplemental Figure 2. *Mettl3* K480R mouse generation strategy. A. Schematic representation of sgRNA and donor oligo used to generate METTL3 K480R knock in mouse using CRISPR-Cas9. The K480R (AAG to CGC) point mutation was introduced into exon 8. **B.** Genotyping using tail-tip DNA and identification of *Mettl3*$^{K480R/+}$ mouse by Sanger sequencing. **C.** Summary of numbers of three genotypes obtained through breeding of heterozygous mice.

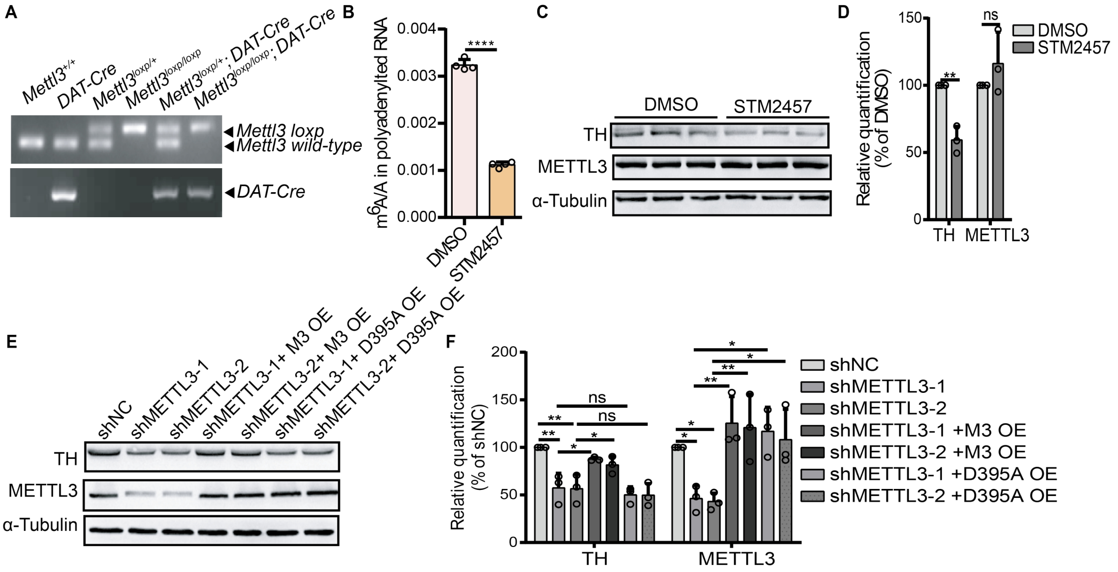

Supplemental Figure 3. ***Mettl3*** **depletion in DA neurons recapitulates neurodegenerative and levodopa-responsive parkinsonism phenotypes. A.** Genotyping using tail-tip DNA for the *Mettl3*$^{+/+}$, DAT-Cre, *Mettl3*$^{loxp/+}$, *Mettl3*$^{loxp/loxp}$, *Mettl3*$^{loxp/+}$*;* DAT-Cre, and *Mettl3*$^{loxp/loxp}$; DAT-Cre mice. **B.** LC–MS/MS quantification of mRNA m^{6}A abundance in DMSO and STM2457 treated SH-SY5Y cells (n = 4). **C.** Western blots showing the protein level of TH and METTL3 in DMSO and STM2457 treated SH-SY5Y cells. **D.** Protein quantification corresponding to C (n = 3). **E.** Western blots showing the protein level of TH and METTL3 in shRNA-mediated knockdown of METTL3 within METTL3 or D395A overexpressed SH-SY5Y cells. **F.** Quantification corresponding to E (n = 3). Data shown are mean ± s.d.. Statistical: , 2-tailed Student's t test (**B**, **D**), 1-way ANOVA followed by Holm-Šidák multiple-comparisons test (**F**).

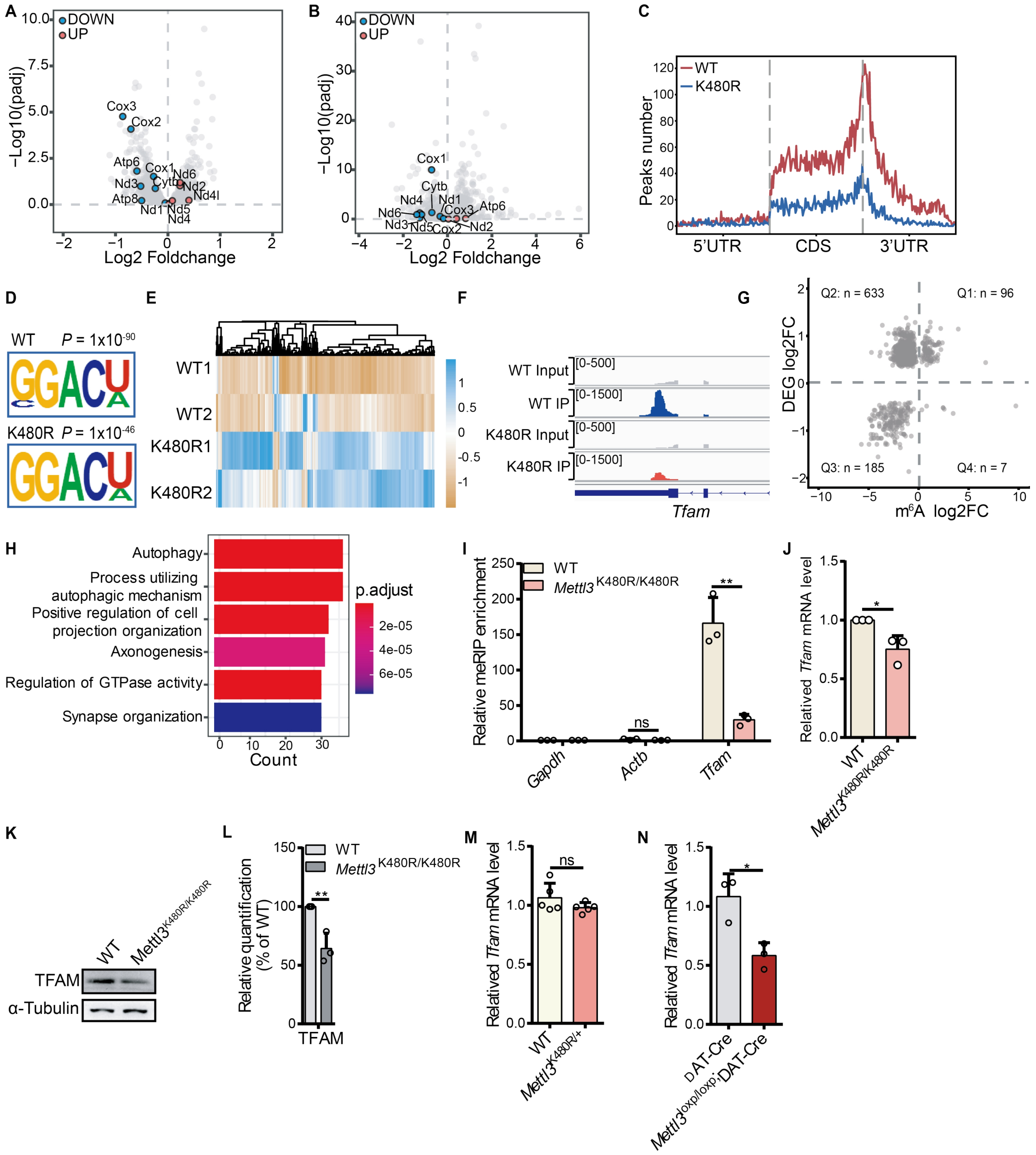

Supplemental Figure 4. Integrated analysis of m^{6}A-seq and RNA-seq data from m^{6}A-deficient cells. A. Expression foldchange of mitochondrial genes in the SN from six-month-old DAT-Cre and *Mettl3*$^{\text{loxp/loxp}}$; DAT-Cre mice, with blue dots indicating downregulated genes encoded by the mitochondrial genome and red dots indicating upregulated genes. **B.** Expression foldchange of mitochondrial genes in *Mettl3*$^{\text{K480R/K480R}}$ mESCs, with blue dots indicating downregulated genes encoded by the mitochondrial genome and red dots indicating upregulated genes. **C.** Distribution of m^{6}A peaks from WT and K480R in transcriptomic regions from MeRIP-seq. **D.** Predominant consensus motifs identified in m^{6}A peaks in WT and K480R mESCs from MeRIP-seq. **E.** Clustered heatmap showing the differential m^{6}A peaks in WT and K480R mESCs. **F.** IGV profiles displaying a m^{6}A differential peak in *Mettl3*$^{\text{K480R/K480R}}$ mESCs in the 3' UTR of *Tfam*. **G.** Integrated analysis of differentially modified m^{6}A peaks in *Mettl3*$^{\text{K480R/K480R}}$ m^{6}A-seq and significant differentially expressed genes in RNA-seq data from the *Mettl3*$^{\text{loxp/loxp}}$; DAT-Cre mice.

H. GO enrichment analysis from the harboring K480R-regulated m^{6}A peaks and significant differential expression in the SN from *Mettl3*$^{loxp/loxp}$; DAT-Cre mice. **I.** MeRIP-qPCR analysis of m^{6}A peaks for *Tfam* in *Mettl3*$^{K480R/K480R}$ mESCs (n = 3). Enrichment of MeRIP versus input RNA was normalized against *Gapdh*. **J-K.** *Tfam* mRNA (J) and protein (K) levels in *Mettl3*$^{K480R/K480R}$ mESCs. **L.** Protein quantification corresponding to K (n = 3). **M.** *Tfam* mRNA levels in WT and *Mettl3*$^{K480R/+}$ mice (n = 5). **N.** *Tfam* mRNA levels in the SN from DAT-Cre and *Mettl3*$^{loxp/loxp}$; DAT-Cre mice (n = 3). Data are shown as mean ± s.d., 2-tailed Student's t test.

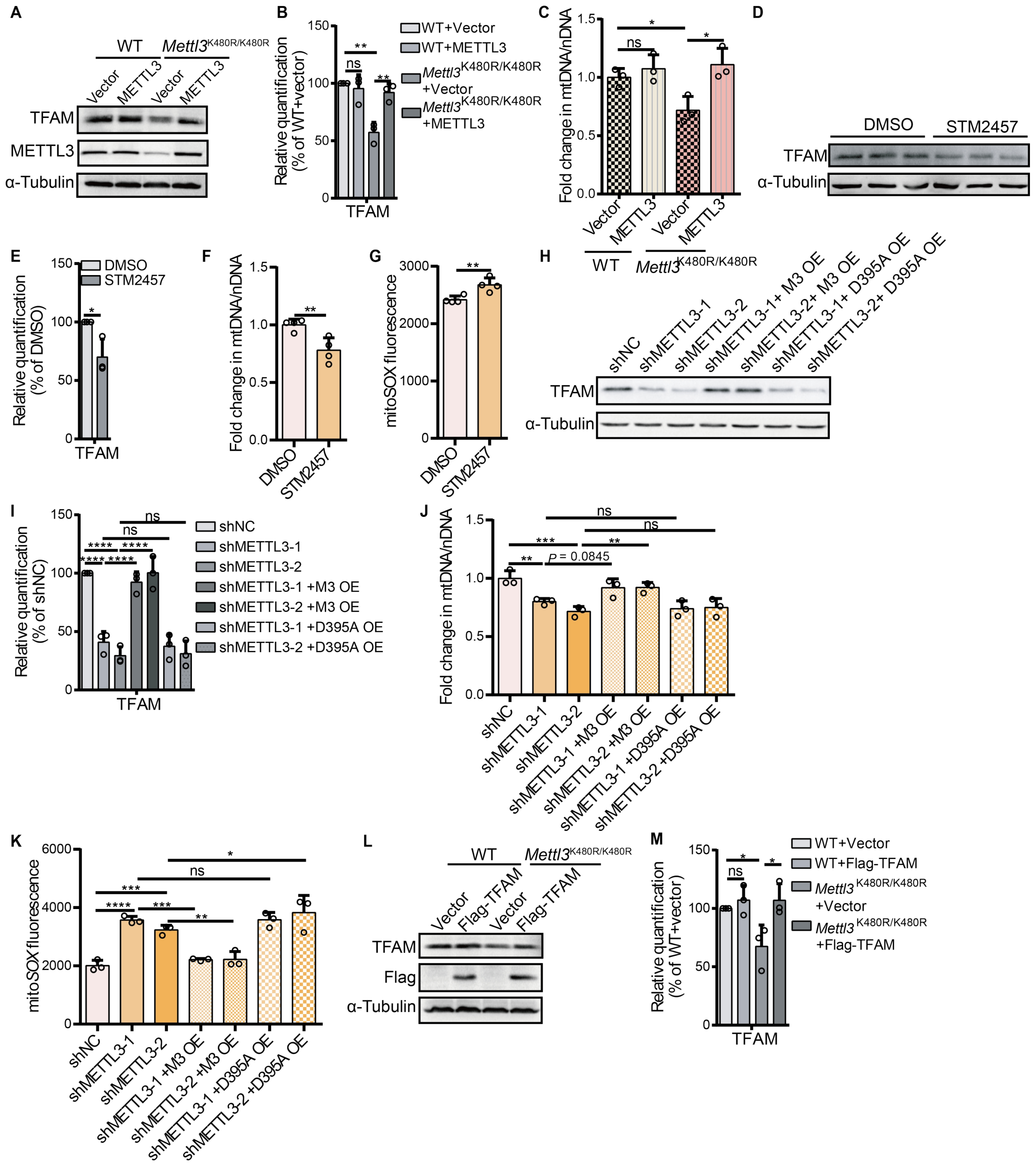

Supplemental Figure 5. m^{6}A deficiency impairs mitochondrial function. A. Western blots showing the protein level of TFAM and METTL3 in WT and *Mettl3*$^{K480R/K480R}$ mESCs overexpressing METTL3. **B.** Protein quantification corresponding to A (n = 3). **C.** mtDNA copy numbers in in WT and *Mettl3*$^{K480R/K480R}$ mESCs overexpressing METTL3 (n = 3). **D.** Western blots showing TFAM protein level in DMSO and STM2457 treated SH-SY5Y cells. **E.** Quantification corresponding to D (n = 3). **F.** mtDNA copy numbers in DMSO and STM2457 treated SH-SY5Y cells (n = 4). **G.** ROS levels in DMSO and STM2457 treated SH-SY5Y cells (n = 4). **H.** Western blots showing TFAM protein level in shRNA-mediated knockdown of METTL3 within METTL3 or D395A overexpressed SH-SY5Y cells. **I.** Quantification corresponding to H (n = 3). **J.** mtDNA copy numbers in shRNA-mediated knockdown of METTL3 within METTL3 or D395A overexpressed SH-SY5Y cells (n = 3) determined by RT-qPCR. **K.** ROS levels in

shRNA-mediated knockdown of METTL3 within METTL3 or D395A overexpressed SH-SY5Y cells (*n* = 3). **L.** Western blots showing the protein level of TFAM in WT and *Mettl3*$^{K480R/K480R}$ mESCs overexpressing TFAM. **M.** Quantification corresponding to L (*n* = 3). Data shown are mean ± s.d.. Statistical: 2-tailed Student's *t* test (**E-G**), 1-way ANOVA followed by Holm-Šidák multiple-comparisons test **(I-K)**, 2-way ANOVA followed by Holm-Šidák multiple-comparisons test **(B**, **C**, **M)**.

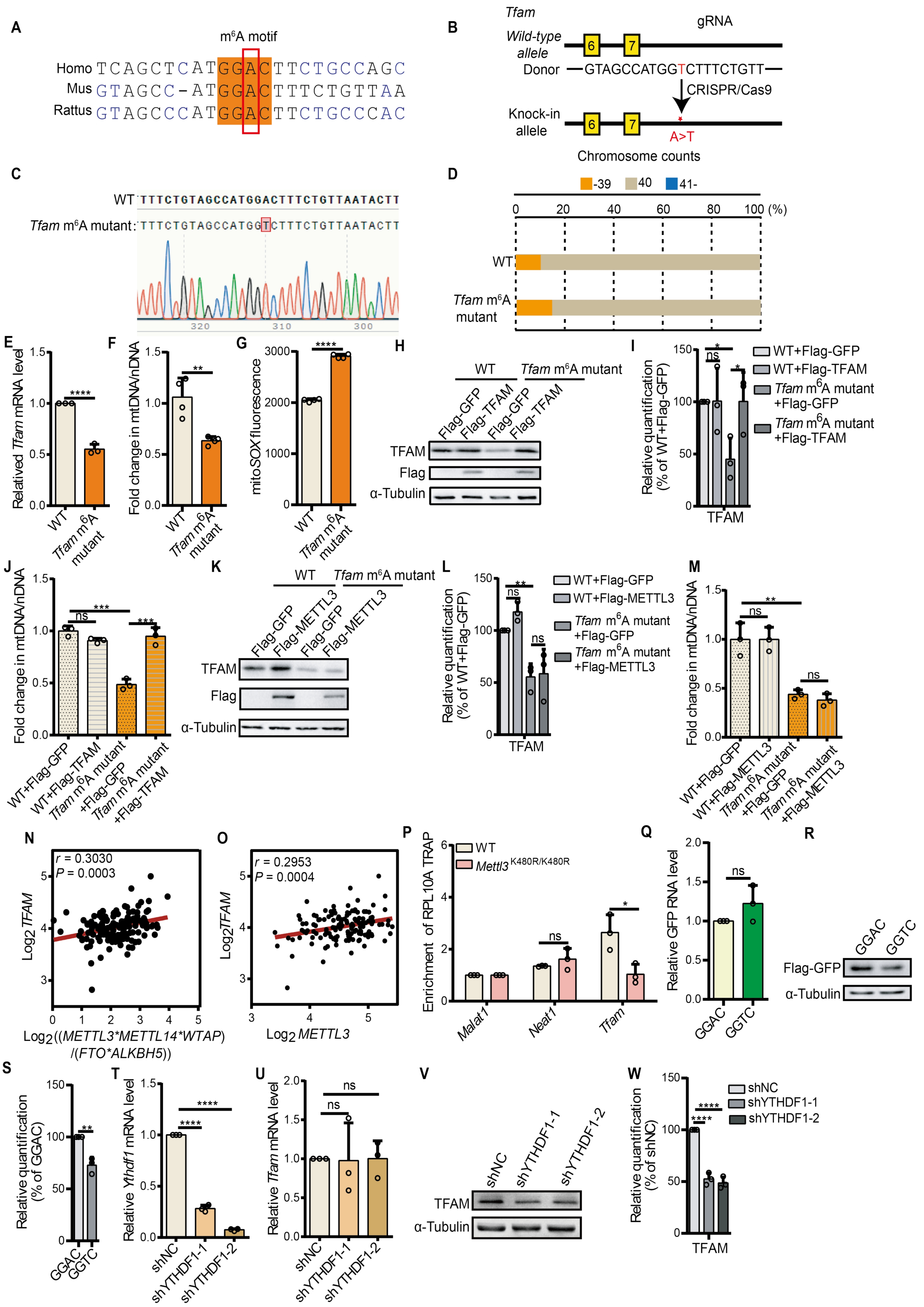

Supplemental Figure 6. m^{6}A deficiency impairs mitochondrial function through TFAM. A. Sequence alignment of TFAM showing conservation of the m^{6}A motif. **B.** Schematic to generate the *Tfam* m^{6}A-site mutation in mESCs. **C.** Sanger sequencing validation of *Tfam* m^{6}A-site mutant clones. **D.** Karyotypes represented by the percentages of the indicated chromosome numbers in WT or *Tfam* m^{6}A mutant cells. **E-G.** *Tfam* mRNA (E; n = 3), mtDNA copy number (F; n = 4), and ROS levels (G; n

= 4) in *Tfam* m^{6}A mutant cells. **H-J**. TFAM overexpression in *Tfam* m^{6}A mutant mESCs: immunoblot (H), quantification of indicated proteins (I; *n* = 3), and mtDNA copy number (J; *n* = 3). **K-M**. METTL3 overexpression in *Tfam* m^{6}A mutant mESCs: immunoblot (K), protein quantification (L; *n* = 3), and mtDNA copy number (M; *n* = 3). **N-O.** Correlations in human substantia nigra (GTEx) between TFAM and an m^{6}A indicator (writers: METTL3+METTL14+WTAP; erasers: FTO+ALKBH5) (N) or between METTL3 and TFAM (O) (Pearson correlation). **P.** TRAP-qPCR analysis of *Tfam* in $Mettl3^{K480R/K480R}$ mESCs (*n* = 3). Enrichment of TRAP versus input RNA was normalized against *Malat1*. **Q-R.** GFP mRNA (Q) and protein (R) levels in GFP reporters fused to the *Tfam* 3′UTR fragment containing either the native m^{6}A motif or a mutated m^{6}A motif cells. **S.** Quantification of protein in R (*n* = 3). **T.** *Ythdf1* mRNA levels in shRNA-mediated knockdown of YTHDF1 cells. **U-V.** *Tfam* mRNA (U) and protein (V) levels in YTHDF1 knockdown mESCs. **W.** Quantification of protein in V (*n* = 3). Data shown are mean ± s.d.. Statistical: 2-tailed Student's *t* test (**E-G, P**, **Q**, **S**), 1-way ANOVA followed by Holm-Šidák multiple-comparisons test (**T**, **U**, **W**), 2-way ANOVA followed by Holm-Šidák multiple-comparisons test **(I**, **J**, **L**, **M)**.

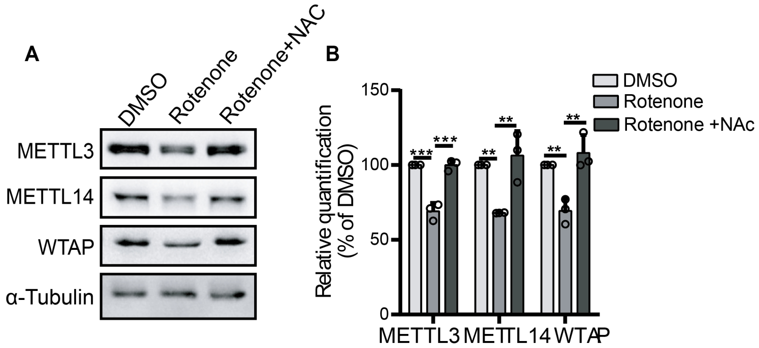

Supplemental Figure 7. Mitochondrial dysfunction reciprocally contributes to METTL3 reduction and m^{6}A deficiency. A. Western blots showing the protein levels of METTL3, METTL14, and WTAP in SH-SY5Y cells treated with rotenone or rotenone plus NAC for 24 h. **B.** Protein quantification corresponding to A (n = 3). Data shown are mean ± s.d., 1-way ANOVA followed by Holm-Šidák multiple-comparisons test.

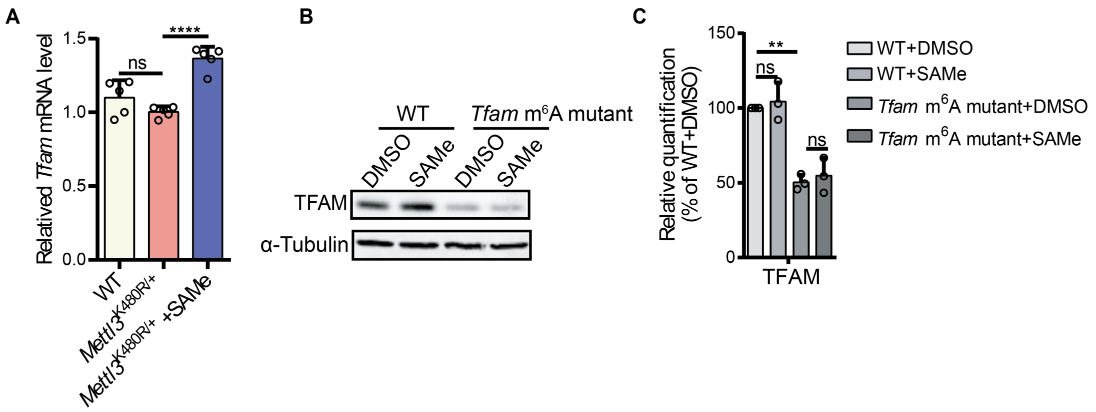

Supplemental Figure 8. SAMe replenishment mitigates parkinsonism in mouse models. A. *Tfam* mRNA levels in the SN from six-month-old WT or *Mettl3*$^{\mathrm{K480R/+}}$ mice treated with SAMe for 2 months. **B.** Western blots showing the protein level of TFAM in WT or *Tfam* m^{6}A mutant cells treated with SAMe. **C.** Protein quantification corresponding to B ($n = 3$). Data shown are mean ± s.d., 1-way ANOVA followed by Holm-Šidák multiple-comparisons test (A), 2-way ANOVA followed by Holm-Šidák multiple-comparisons test (C).

References

1. Bolger AM, Lohse M, and Usadel B. Trimmomatic: a flexible trimmer for Illumina sequence data. *Bioinformatics.* 2014;30(15):2114-20.
2. Li H, and Durbin R. Fast and accurate short read alignment with Burrows-Wheeler transform. *Bioinformatics.* 2009;25(14):1754-60.
3. Van der Auwera GA, Carneiro MO, Hartl C, Poplin R, Del Angel G, Levy-Moonshine A, et al. From FastQ data to high confidence variant calls: the Genome Analysis Toolkit best practices pipeline. *Curr Protoc Bioinformatics.* 2013;43(1110):11.0.1-.0.33.
4. Wang K, Li M, and Hakonarson H. ANNOVAR: functional annotation of genetic variants from high-throughput sequencing data. *Nucleic Acids Res.* 2010;38(16):e164.
5. Deng H, Wang P, and Jankovic J. The genetics of Parkinson disease. *Ageing Res Rev.* 2018;42:72-85.
6. Zhou W, Bi W, Zhao Z, Dey KK, Jagadeesh KA, Karczewski KJ, et al. SAIGE-GENE+ improves the efficiency and accuracy of set-based rare variant association tests. *Nat Genet.* 2022;54(10):1466-9.
7. Gordon LJ, Allen M, Artursson P, Hann MM, Leavens BJ, Mateus A, et al. Direct Measurement of Intracellular Compound Concentration by RapidFire Mass Spectrometry Offers Insights into Cell Permeability. *J Biomol Screen.* 2016;21(2):156-64.
8. Taguchi T, Ikuno M, Hondo M, Parajuli LK, Taguchi K, Ueda J, et al. α-Synuclein BAC transgenic mice exhibit RBD-like behaviour and hyposmia: a prodromal Parkinson's disease model. *Brain.* 2020;143(1):249-65.
9. Kim S, Kwon SH, Kam TI, Panicker N, Karuppagounder SS, Lee S, et al. Transneuronal Propagation of Pathologic α-Synuclein from the Gut to the Brain Models Parkinson's Disease. *Neuron.* 2019;103(4):627-41.e7.
10. Heiman M, Kulicke R, Fenster RJ, Greengard P, and Heintz N. Cell type-specific mRNA purification by translating ribosome affinity purification (TRAP). *Nat Protoc.* 2014;9(6):1282-91.

Table S1. List of oligonucleotides used in this study

Primer	Sequence (5’-3’)
hMETTL3-F1	gagctcaagcttcgaattcatgtcggacacgtggagctctat
hMETTL3-R1	gtctttgtagtcggatcctaaattcttaggtttagagatg
hMETTL3-mutF1	cgtgtctatactcggtaaatttgcagttgtgatggctgacccac
hMETTL3-mutR1	aatttaccgagtatagacacgtccaggtagcggatatcacaacag
hMETTL3-mutF2	agccaaggaacaatcaatcttgaaaaatttcgctctcgaggtcg
hMETTL3-mutR2	aagattgattgttccttggctgttgtagtatt
mTfam-F1	gagctcaagcttcgaattcatggcgctgttccggggaatgtg
mTfam-R1	ggtctttgtagtcggatccatgctcagagatgtctccggatcg
mMettl3-F1	gagctcaagcttcgaattcatgtcggacacgtggagctctatc
mMettl3-R1	gtctttgtagtcggatcctaaattcttaggtttagagatg
GFP-F1	agctcaagcttcgaattcatggtgagcaagggcgaggagct
GFP-R1	gtctttgtagtcggatcccttgtacagctcgtccatgccgag
METTL3 K480R-F1	gggaacactgcttggttggtgtcaaaggaaatcccc
METTL3 K480R-R1	caaccaagcagtgttccctcccatggttcaaccagt
SFB-METTL3 -F1	gggcggcgcgccatgtcggacacgtggagct
SFB-METTL3 -R1	ccgtctagactataaattcttaggtttagagatgatacc
myc-METTL3-F	cagaggaggacctggaattgatgtcggacacgtggagctctatc
myc-METTL3-R	cgactcactatagttctagactataaattcttaggtttagagatg
METTL3 D395A-F1	gccccaccctgggatattcacat
METTL3 D395A-R1	aatatcccagggtggggcagccatcacaactgcaaacttg
mHK2-gDNA-F	ttctcaatgctagggaggacagc
mHK2-gDNA-R	tcacataagctaacctctgcccc
hCOX2-F	gctgtccccacattaggctt
hCOX2-R	cgatgggcatgaaactgtgg
hHK2-gDNAF	gggaaacttcagggggatcg
hHK2-gDNAR	agagtgccttttgcagtggt
mCOX2-F	aaacctggtgaactacgactgct
mCOX2-R	gttgcttgatttagtcggcctgg
mGAPDH-qF	tggagccaaaagggtcatcatct
mGAPDH-qR	cccttccacaatgccaaagttgt
mACTB-qF	gaaccctaaggccaaccgtga
mACTB-qR	atggcgtgagggagagcata
mTFAM-merip-qF	ccgttacatatgggtgtggc
mTFAM-merip-qR	gaaagcctggcagcttctttg
mTFAM-qF	gggaatgtggagcgtgctaaaag
mTFAM-qR	acccatgctggaaaaacacttcg

Primer	Sequence (5’-3’)
DAT-cre-F	gatctccggtattgaaactccagc
DAT-cre-R	gctaaacatgcttcatcgtcgg
Mettl3 $^{loxp-/-}$-F	ccttccccagatgaaactgtcta
Mettl3 $^{loxp-/-}$-R	gaaaggcacagcactagtcttc
Mettl3 $^{K480R/+}$ -F	tgttcctgccaatctgcccttgtgtc
Mettl3 $^{K480R/+}$ -R	gattgcaattctagtctggtctgtg
mYTHDF1-Q1F	gcatcagaaggatgcagttcatg
mYTHDF1-Q1R	gatggtggatagtaactggacag
Malat1-Q1F	gctgtttcctgctccgagat
Malat1-Q1R	gcagtgtgccaatgtttcgt
Neat1-Q1F	accacagaagaggaagcacg
Neat1-Q1R	tggagattgaaggcgcaagt
EGFP-qF	caagatccgccacaacatcg
EGFP-qR	gactgggtgctcaggtagtg
mTFAM-oliF2	gaagtcttgggaagagcagatgg
mTFAM-1R	tcatctaggaaagcctggcagc
shmYTHDF1-1F	ccggcgacaacaaacctgtcacaaactcgagtttgtgacaggtttgttgtcgtttttg
shmYTHDF1-1R	aattcaaaaacgacaacaaacctgtcacaaactcgagtttgtgacaggtttgttgtcg
shmYTHDF1-2F	ccgggctgaagattatcgcttcctactcgagtaggaagcgataatcttcagctttttg
shmYTHDF1-2R	aattcaaaaagctgaagattatcgcttcctactcgagtaggaagcgataatcttcagc
shhMETTL3-1F	ccggcgtcagtatcttgggcaagttctcgagaacttgcccaagatactgacgtttttg
shhMETTL3-1R	aattcaaaaacgtcagtatcttgggcaagttctcgagaacttgcccaagatactgacg
shhMETTL3-2F	ccggagccaaggaacaatccattgtctcgagacaatggattgttccttggctttttg
shhMETTL3-2R	aattcaaaaaagccaaggaacaatccattgtctcgagacaatggattgttccttggct
