# Supplementary material for: m6A deficiency induces dopaminergic neurodegeneration and progressive parkinsonism through a pathogenic loop with mitochondria
Source: J Clin Invest. 2026 Mar 17;136(9):e197183. doi: 10.1172/JCI197183 (PMC13132373; doi:10.1172/JCI197183)

Full unedited blot for Figure 1H

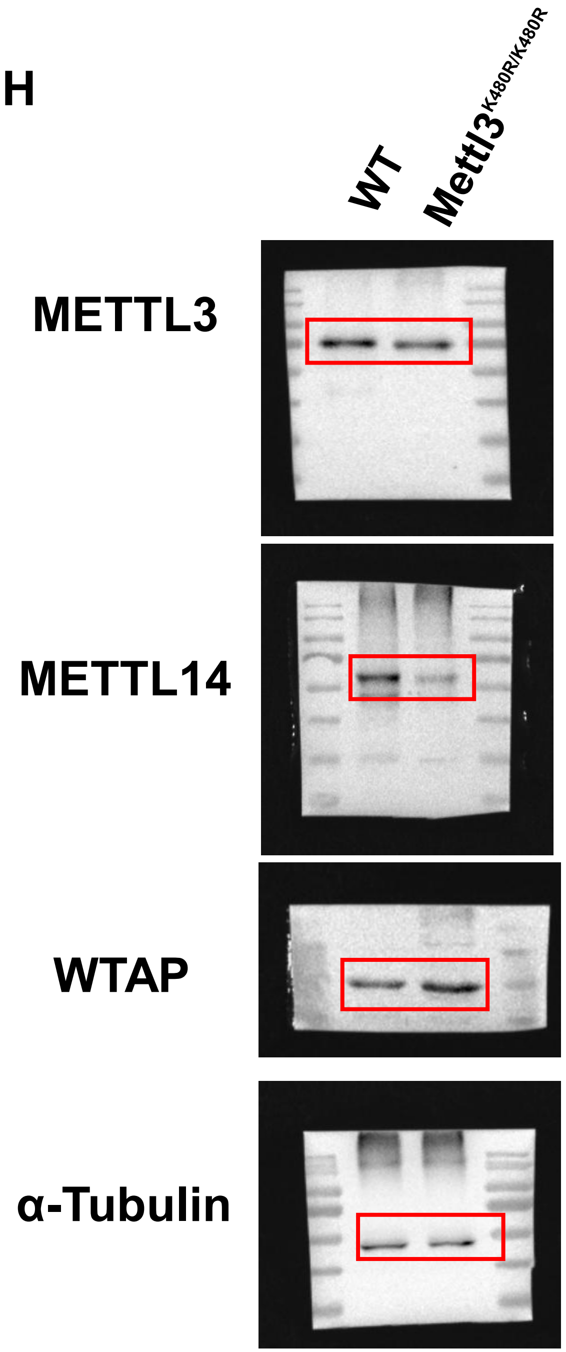

Full unedited blot for Figure 1J

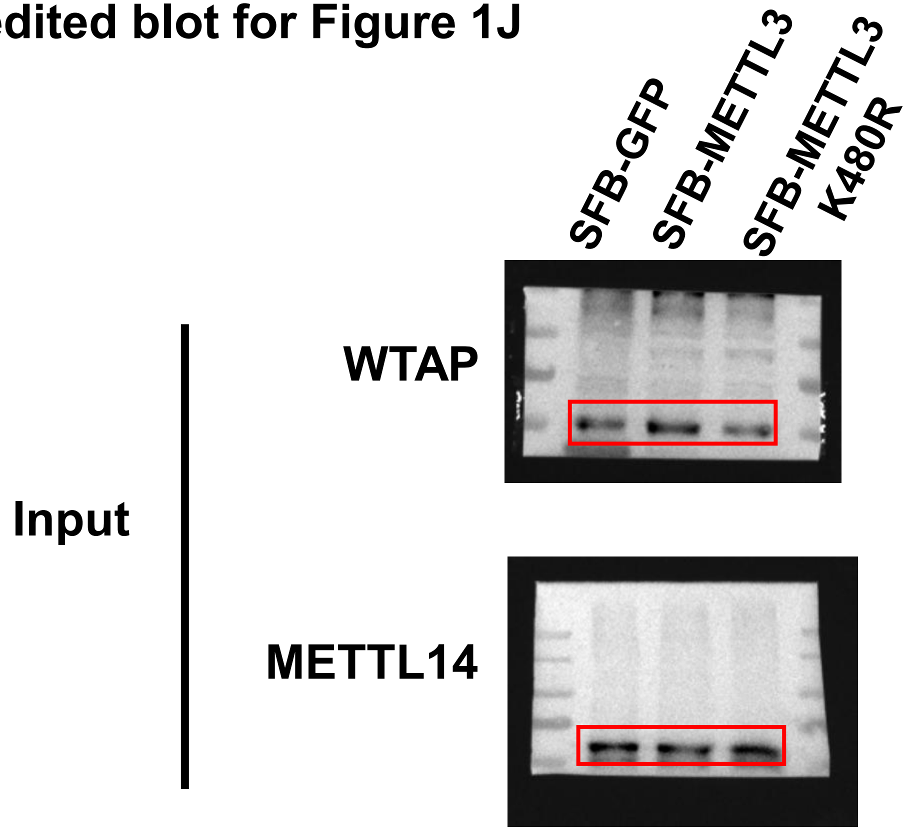

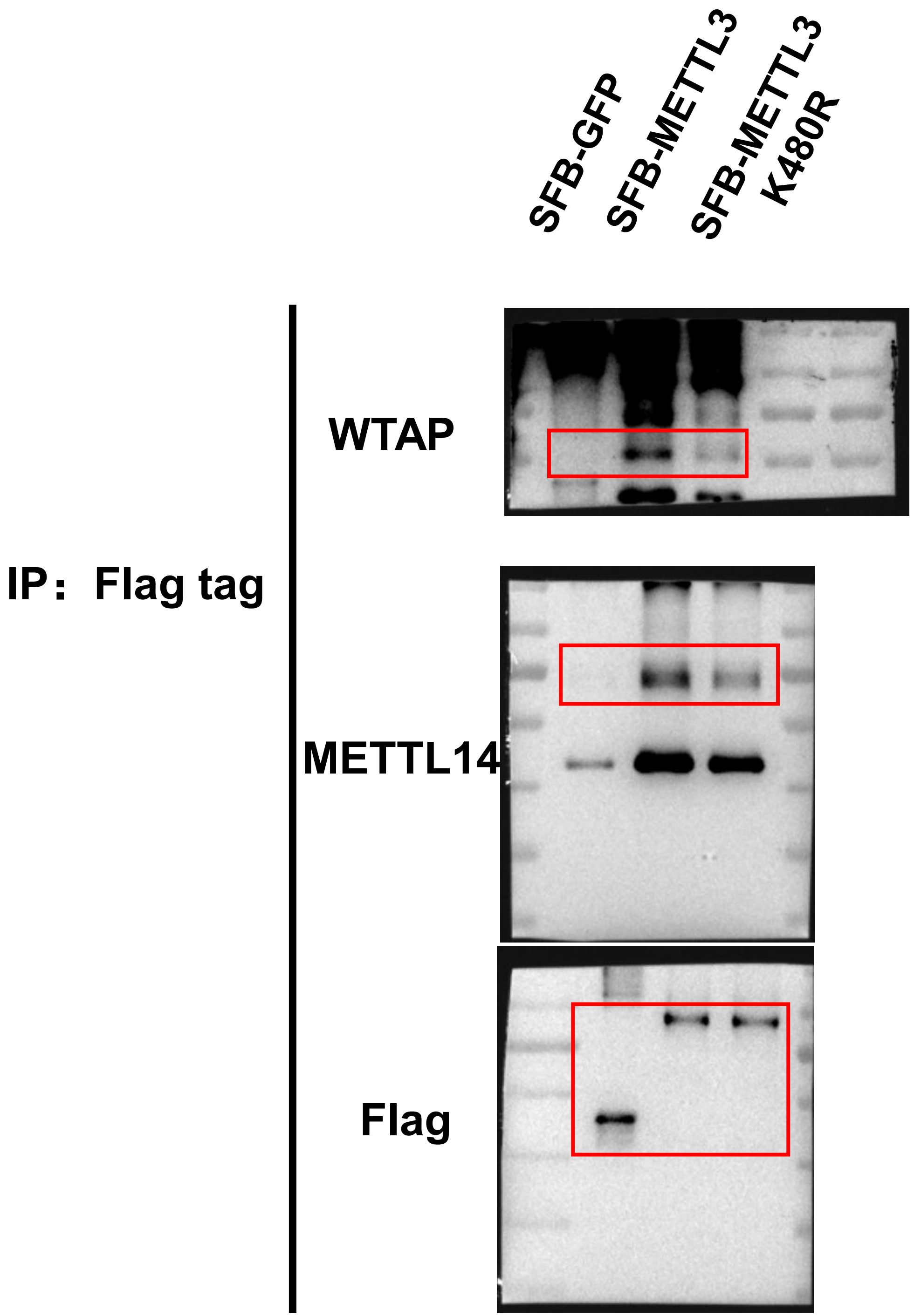

Full unedited blot for Figure 2A

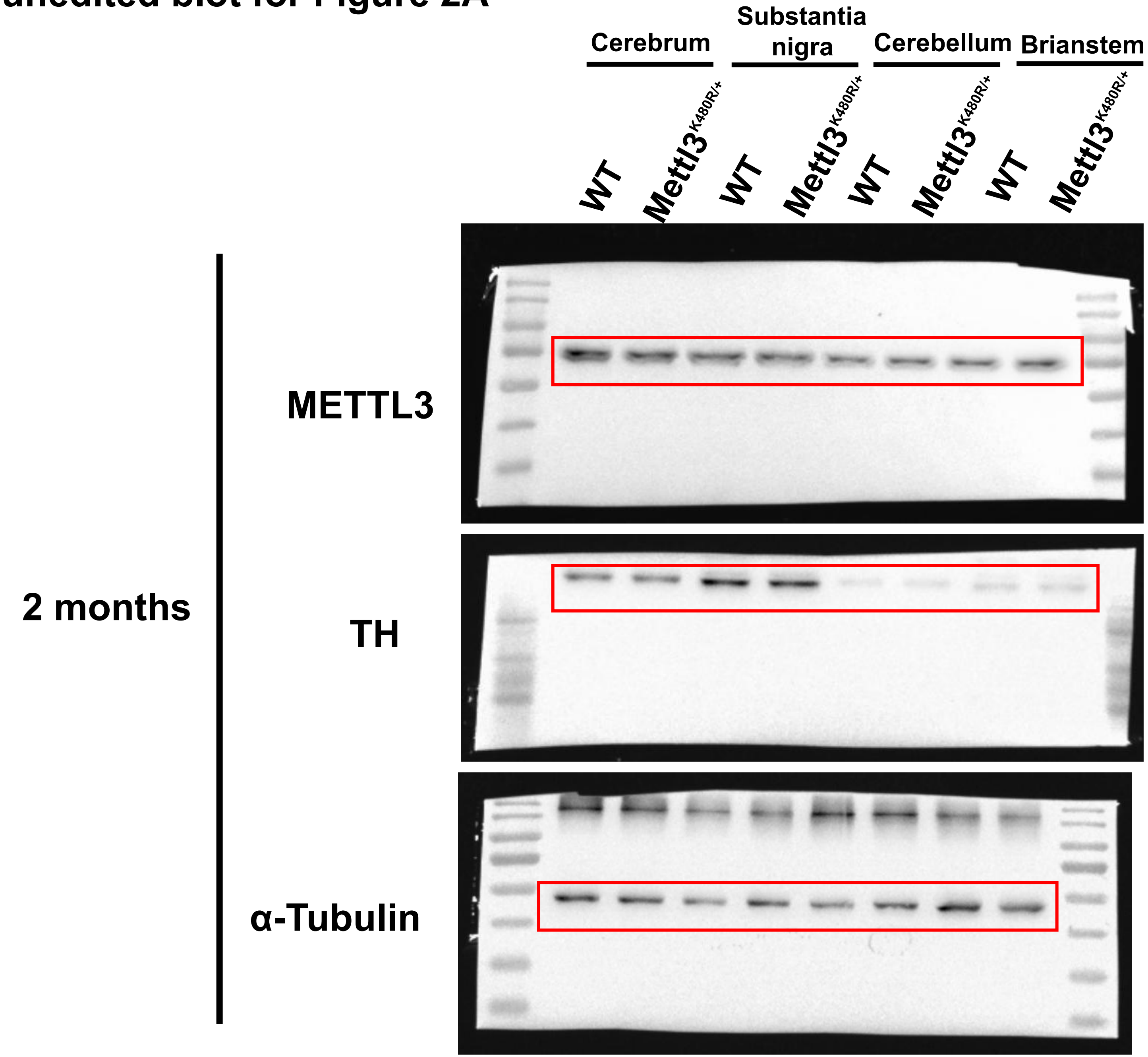

Full unedited blot for Figure 2C

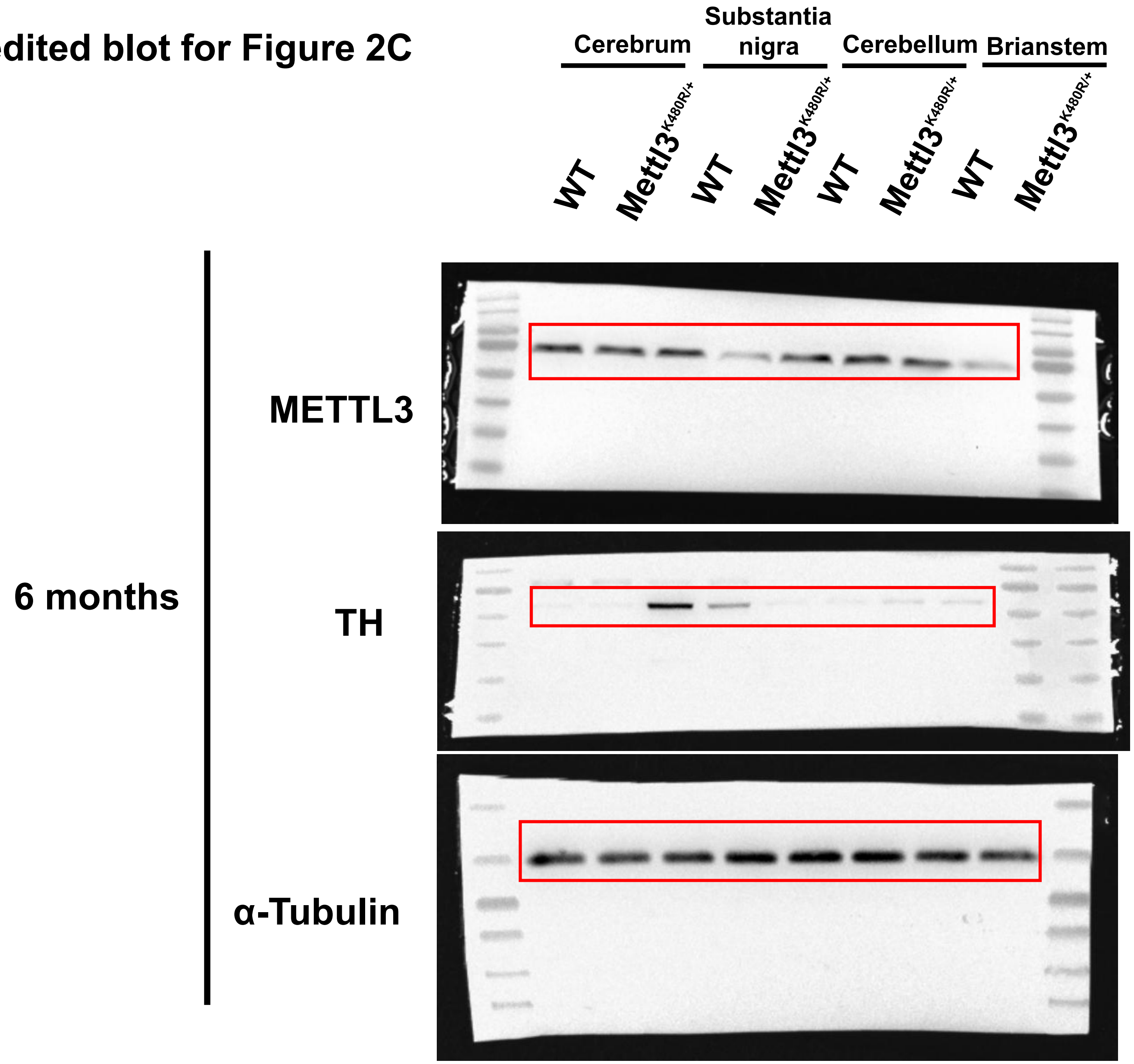

Full unedited blot for Figure 2K

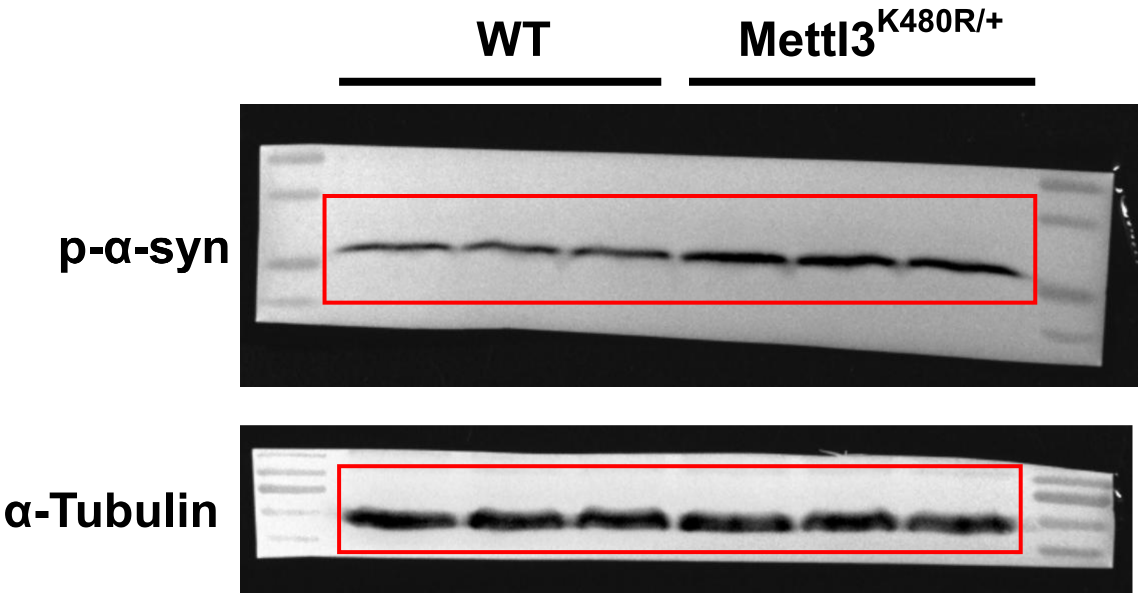

Full unedited blot for Figure 4A

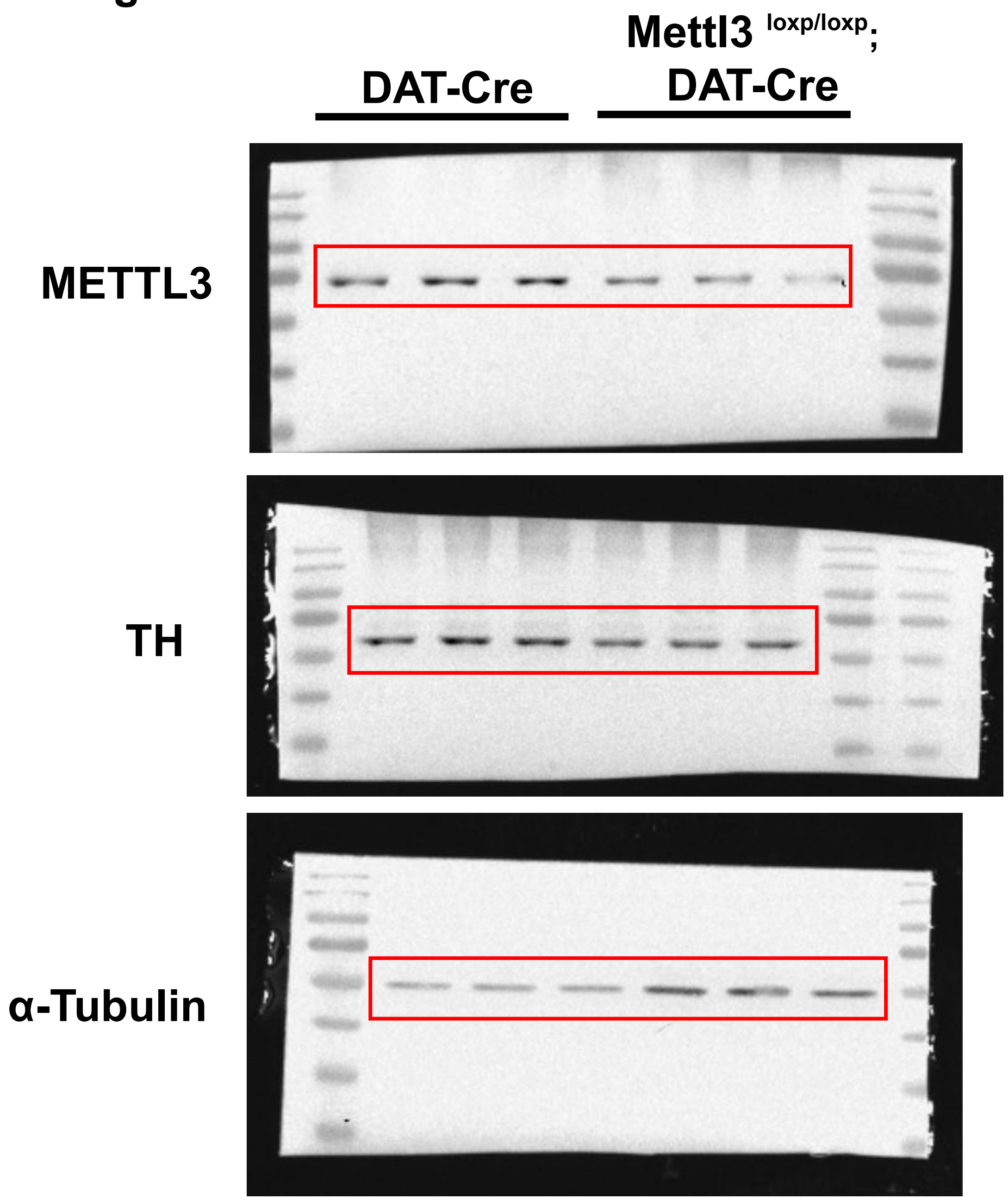

Full unedited blot for Figure 4G

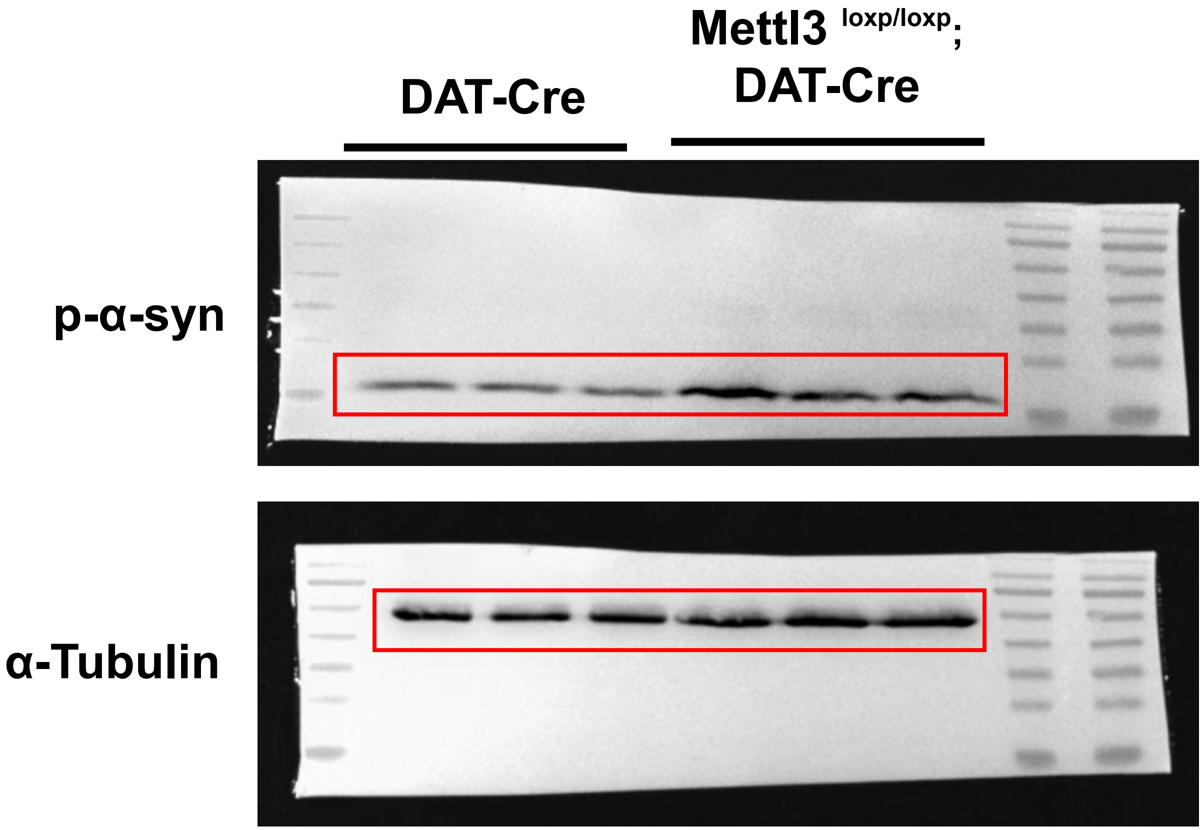

Full unedited blot for Figure 4.I-J

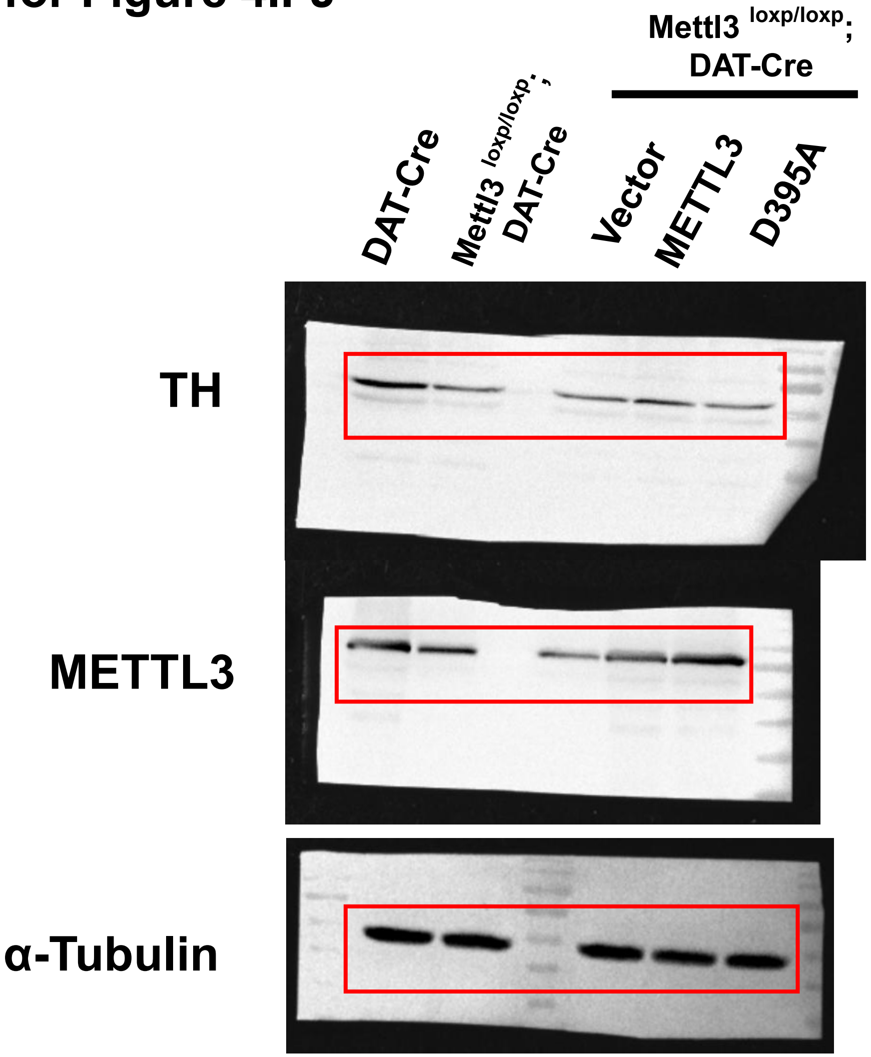

Full unedited blot for Figure 5H

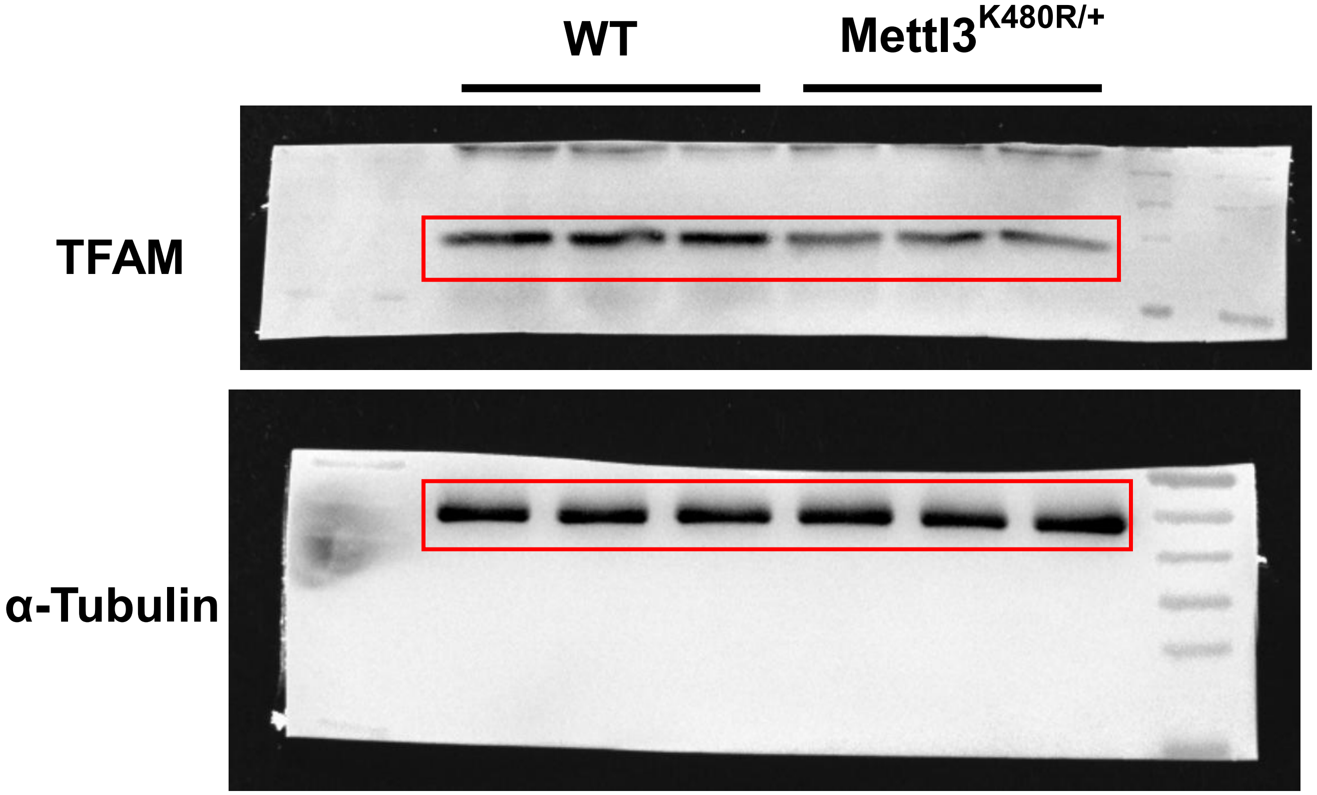

Full unedited blot for Figure 5J

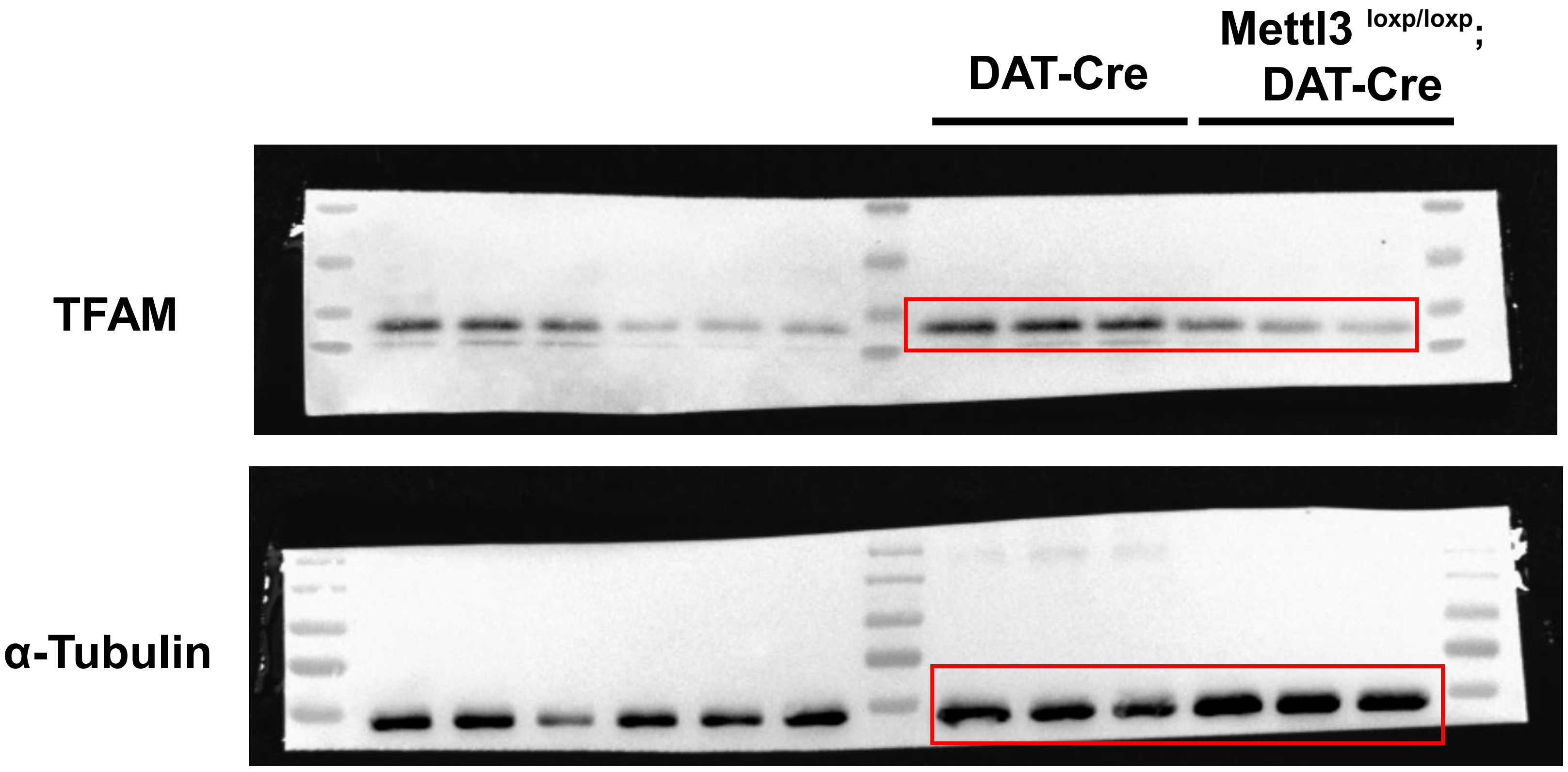

Full unedited blot for Figure 5L-M

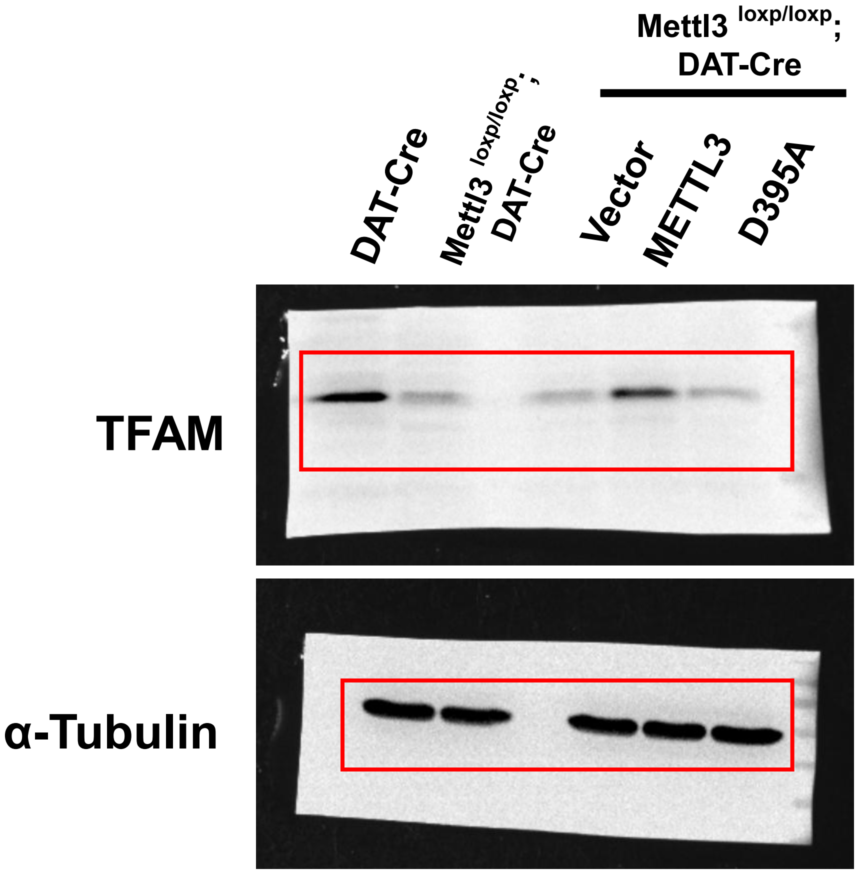

Full unedited blot for Figure 5S

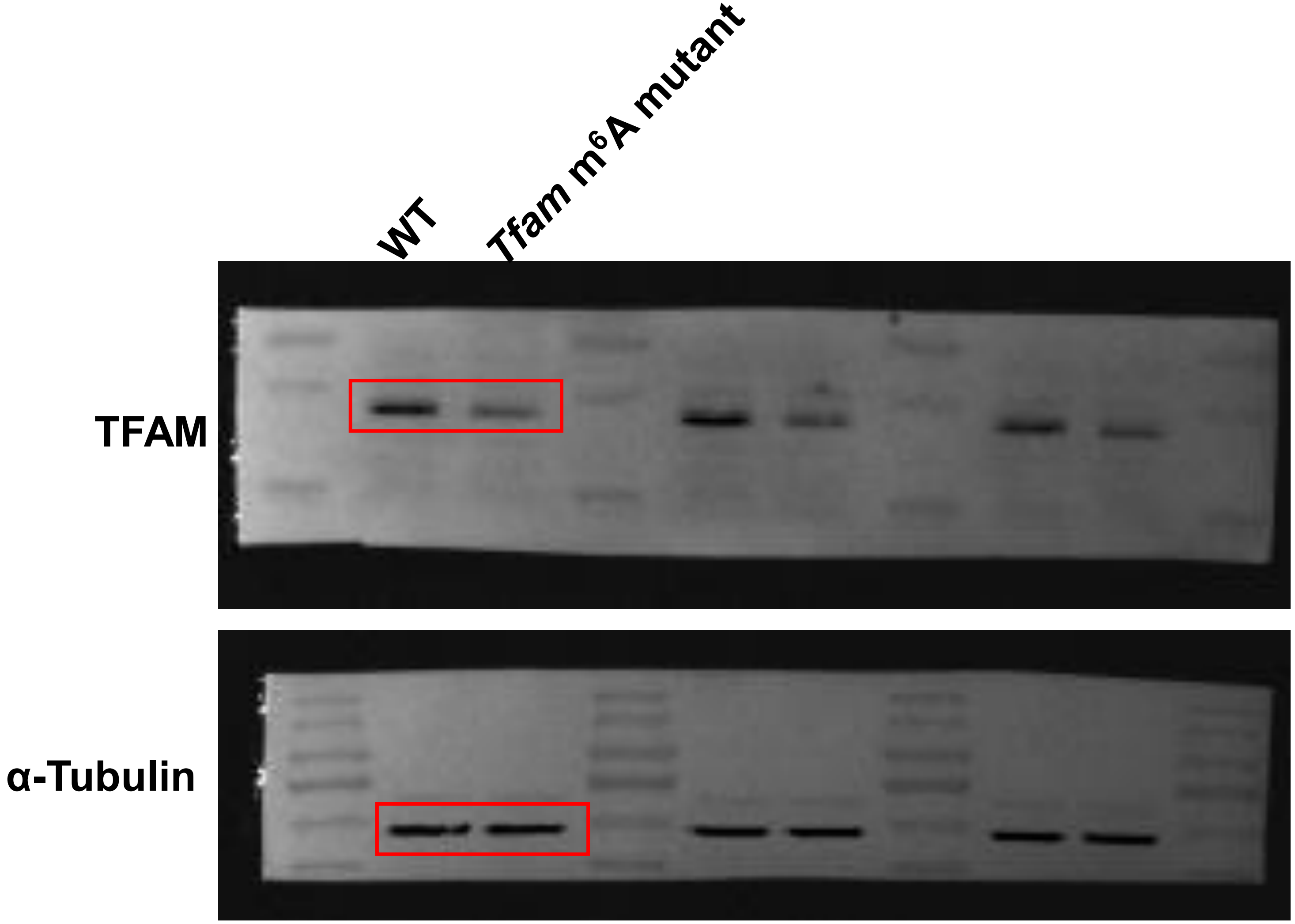

Full unedited blot for Figure 6A

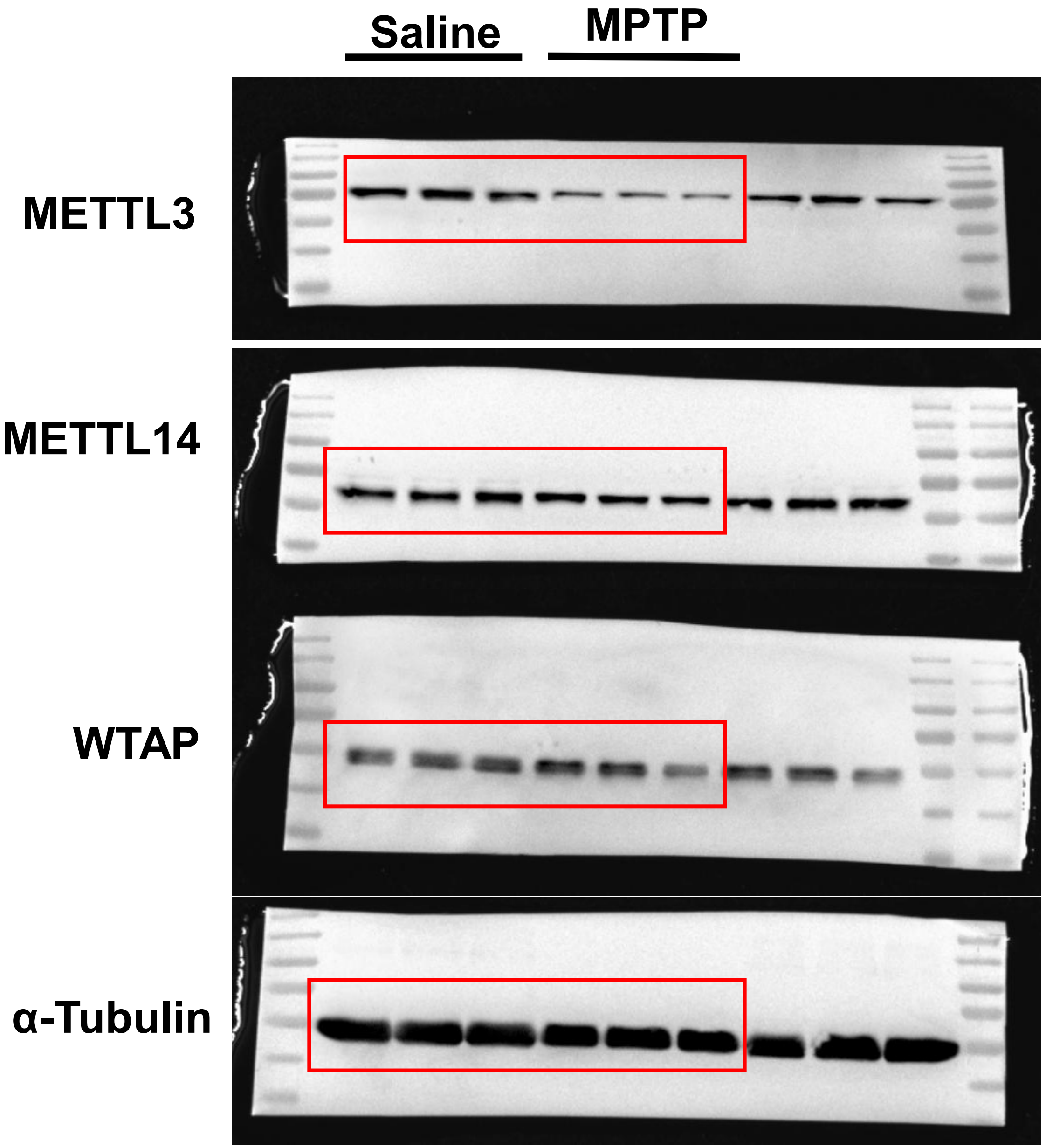

Full unedited blot for Figure 6C

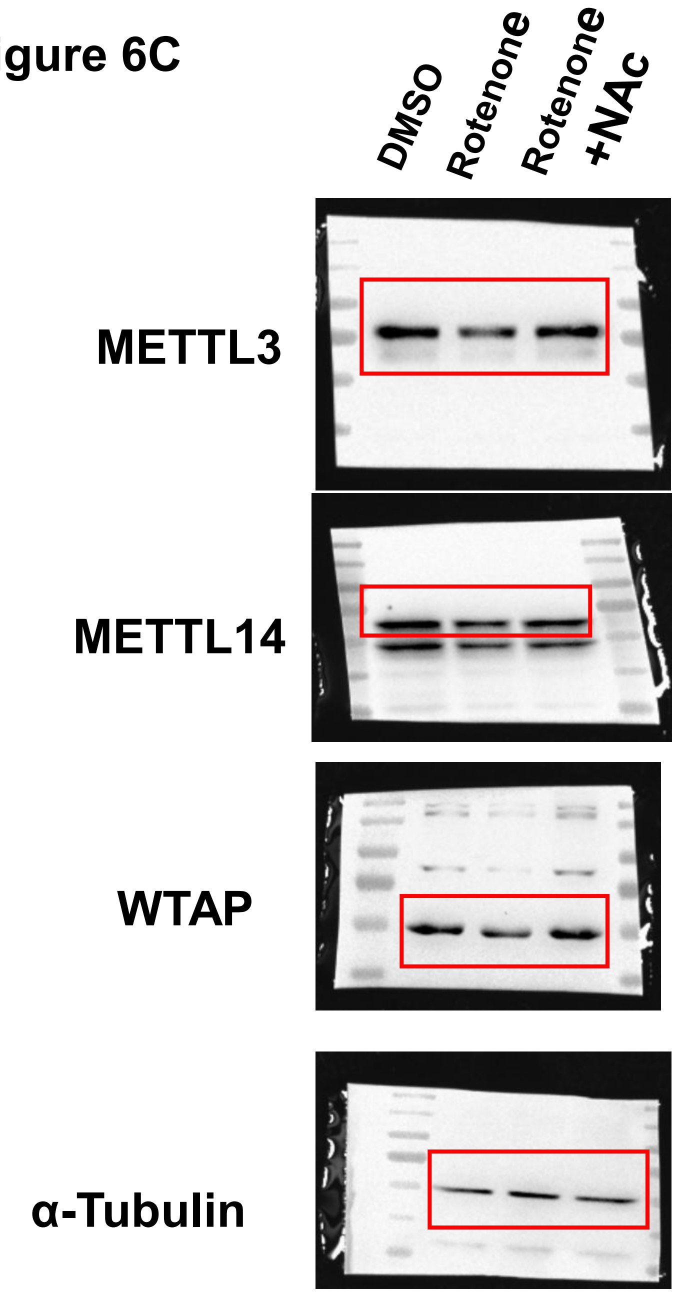

Full unedited blot for Figure 6F

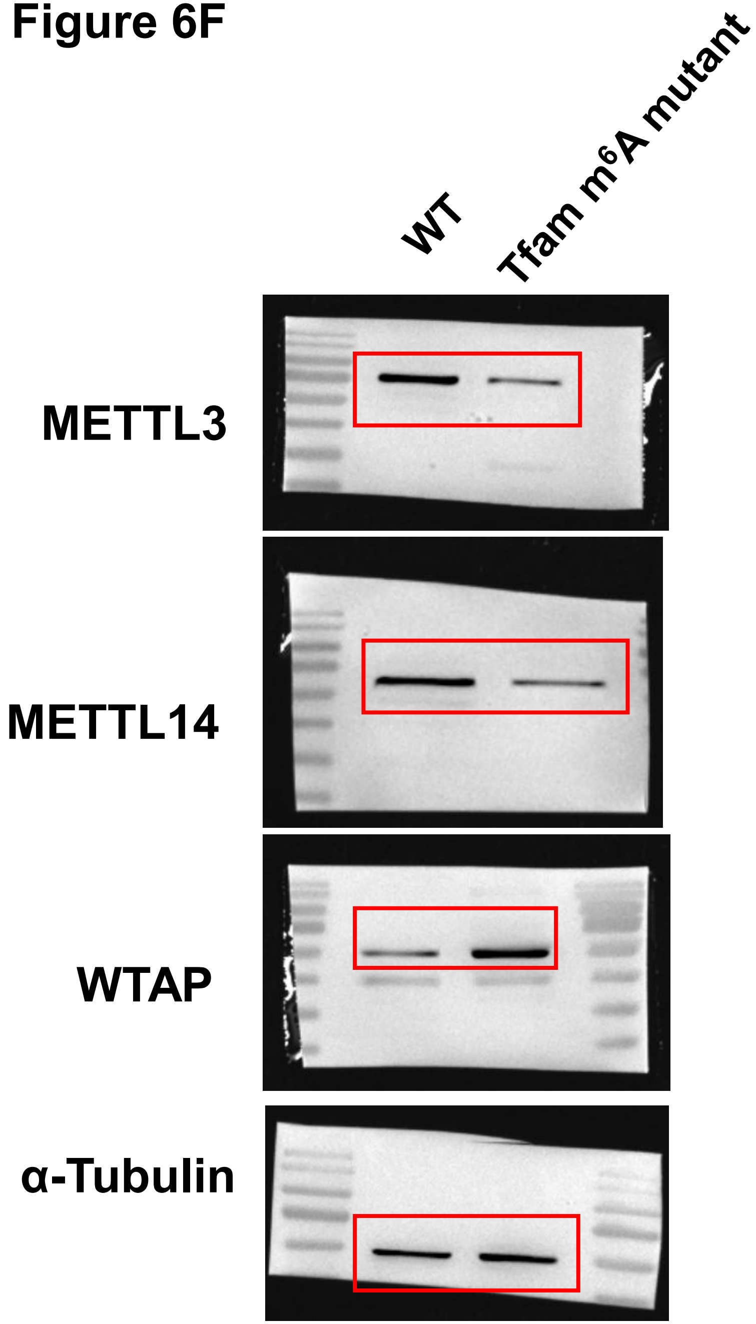

Full unedited blot for Figure 6I

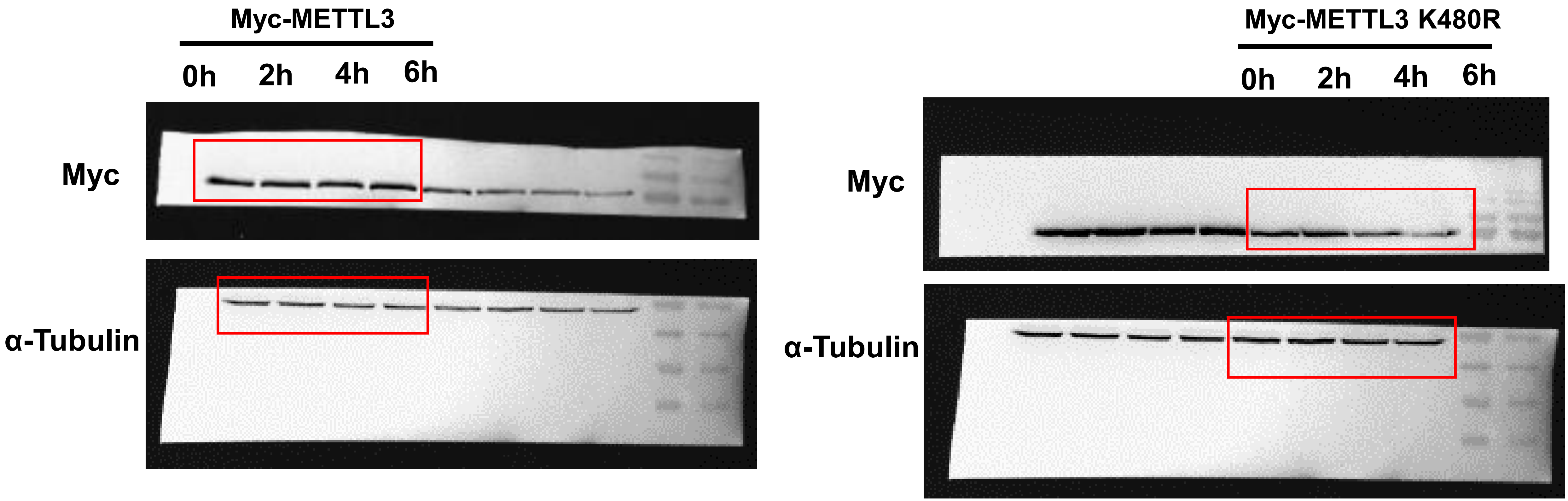

Full unedited blot for Figure 7A

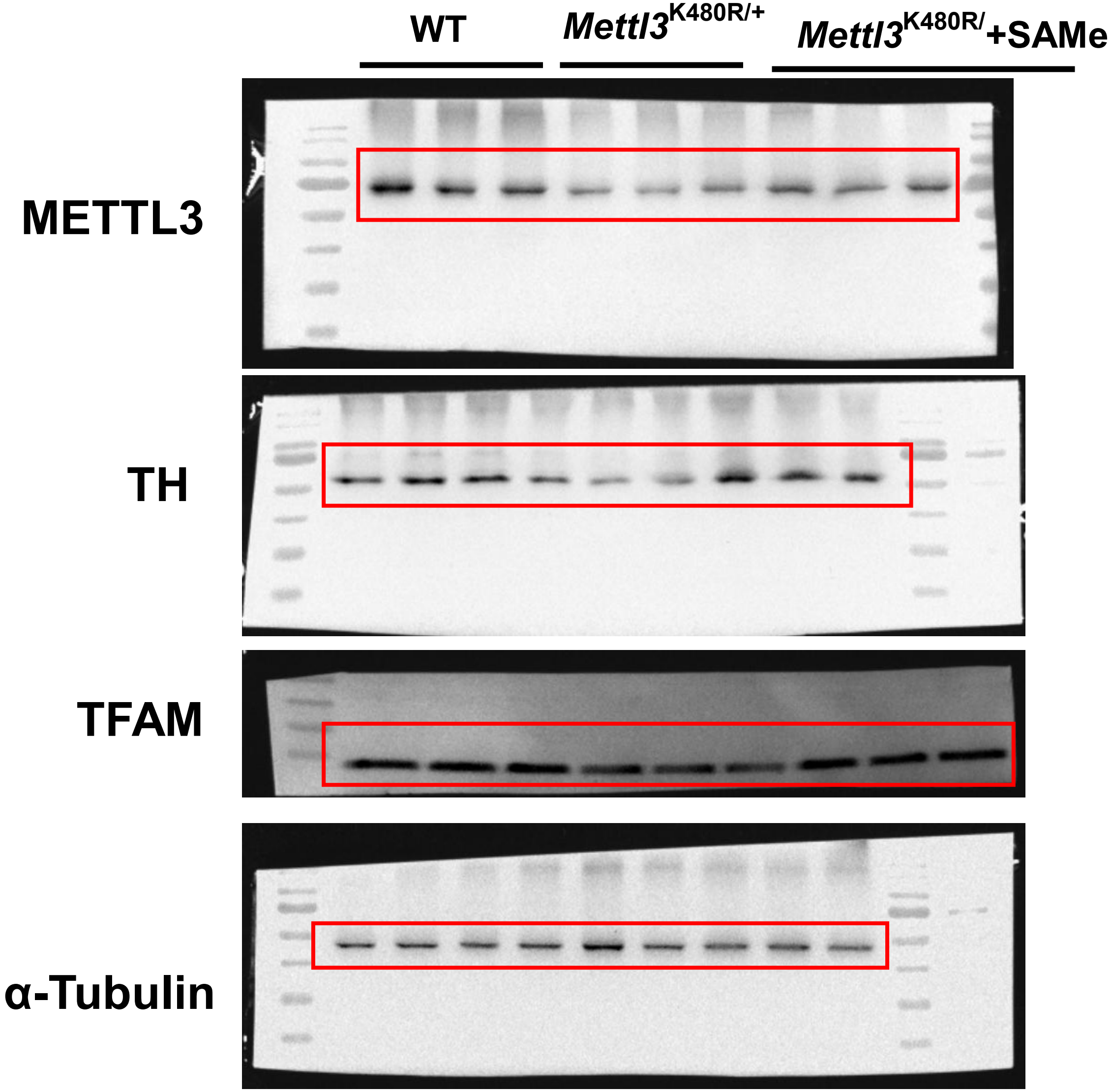

Full unedited blot for Figure 7L

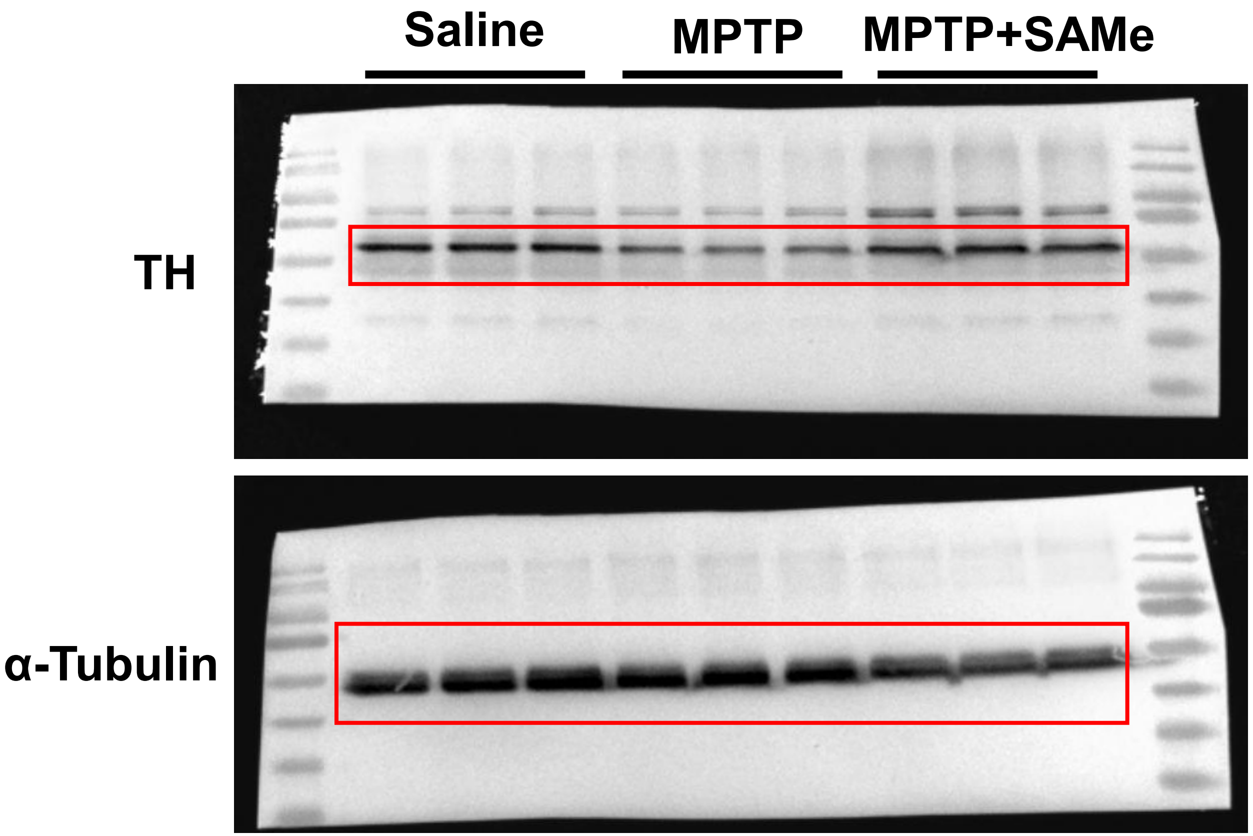

Full unedited blot for Supplemental Figure 1I

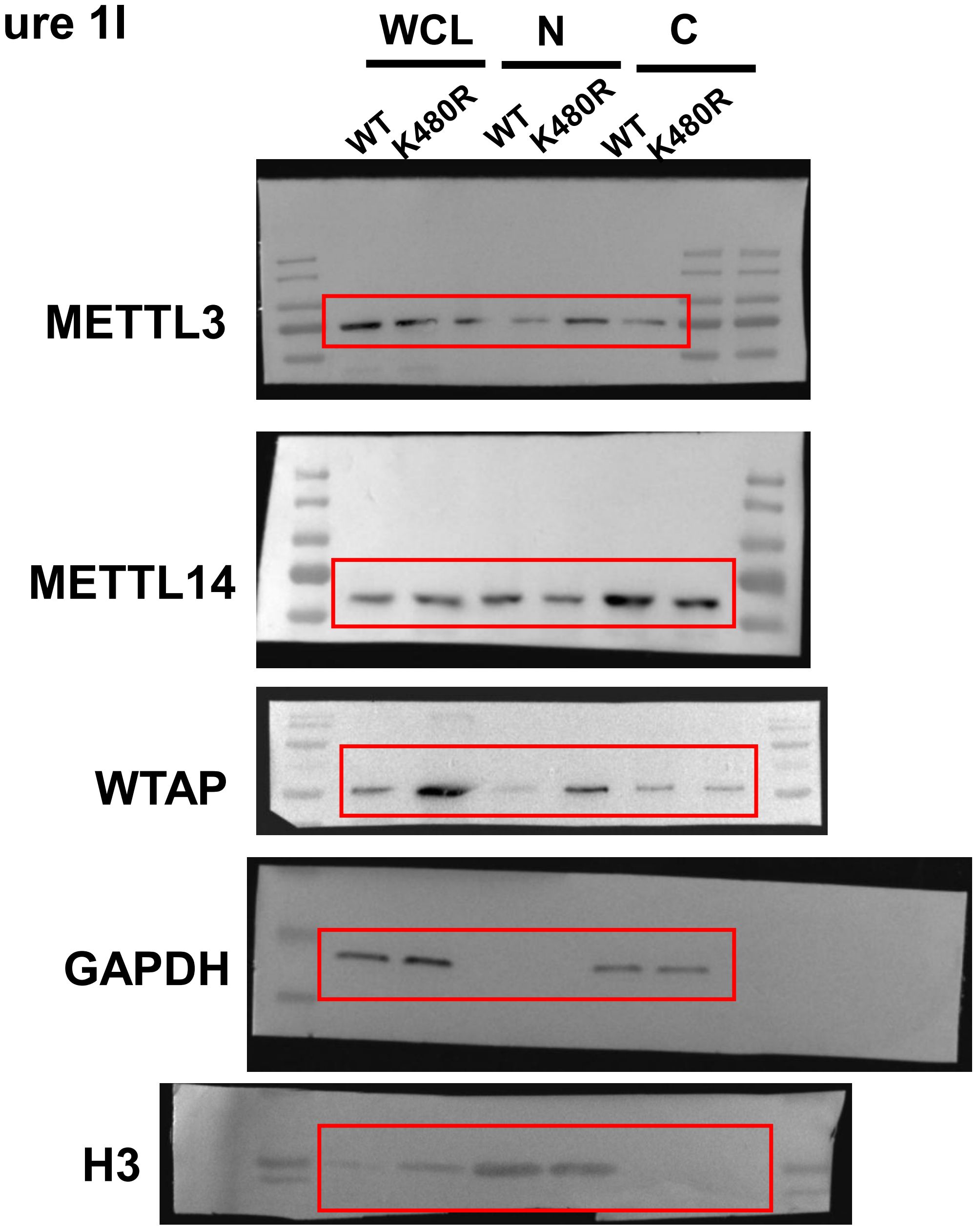

Full unedited gel for Supplemental Figure 3A

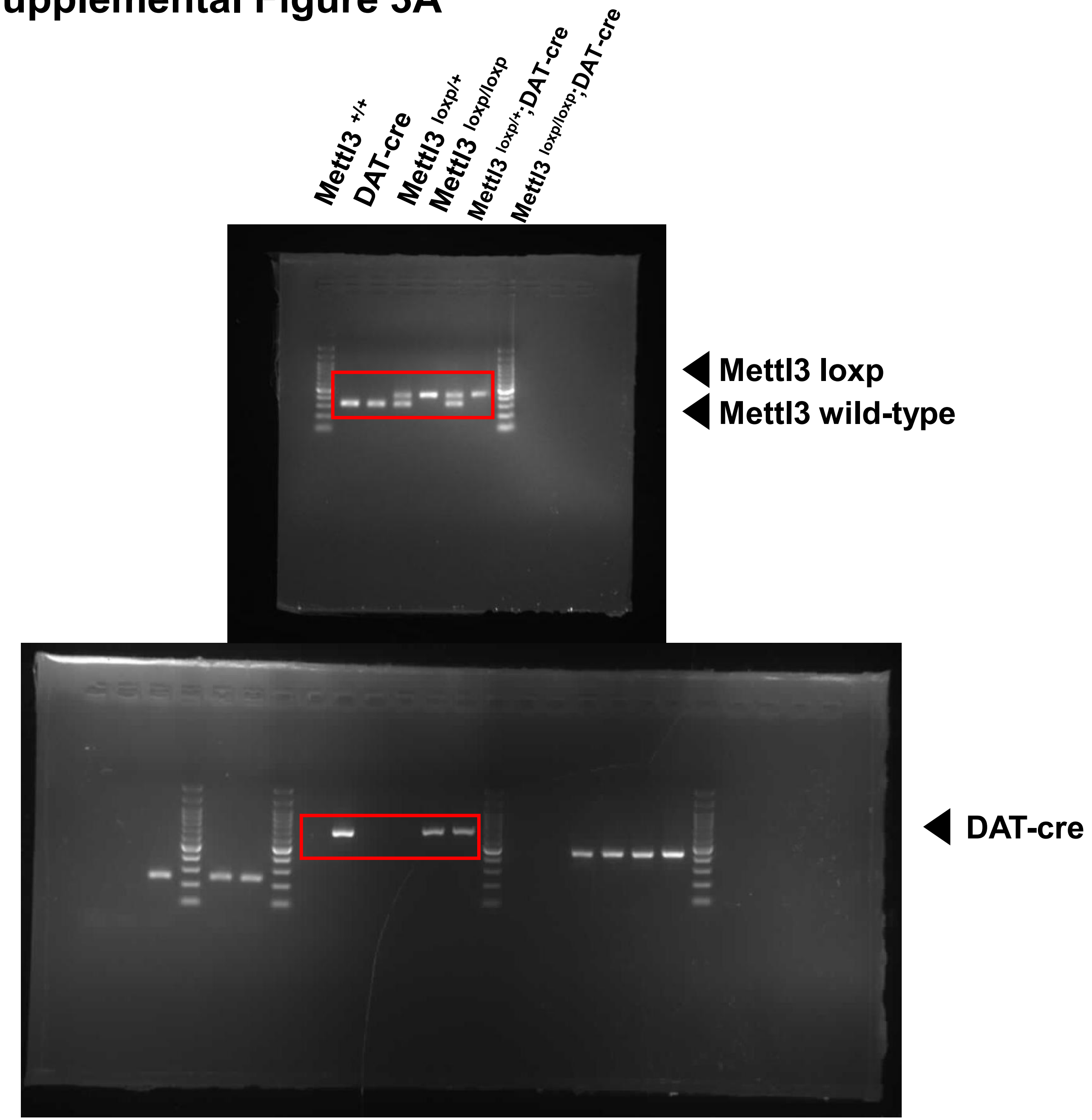

Full unedited blot for Supplemental Figure 3C

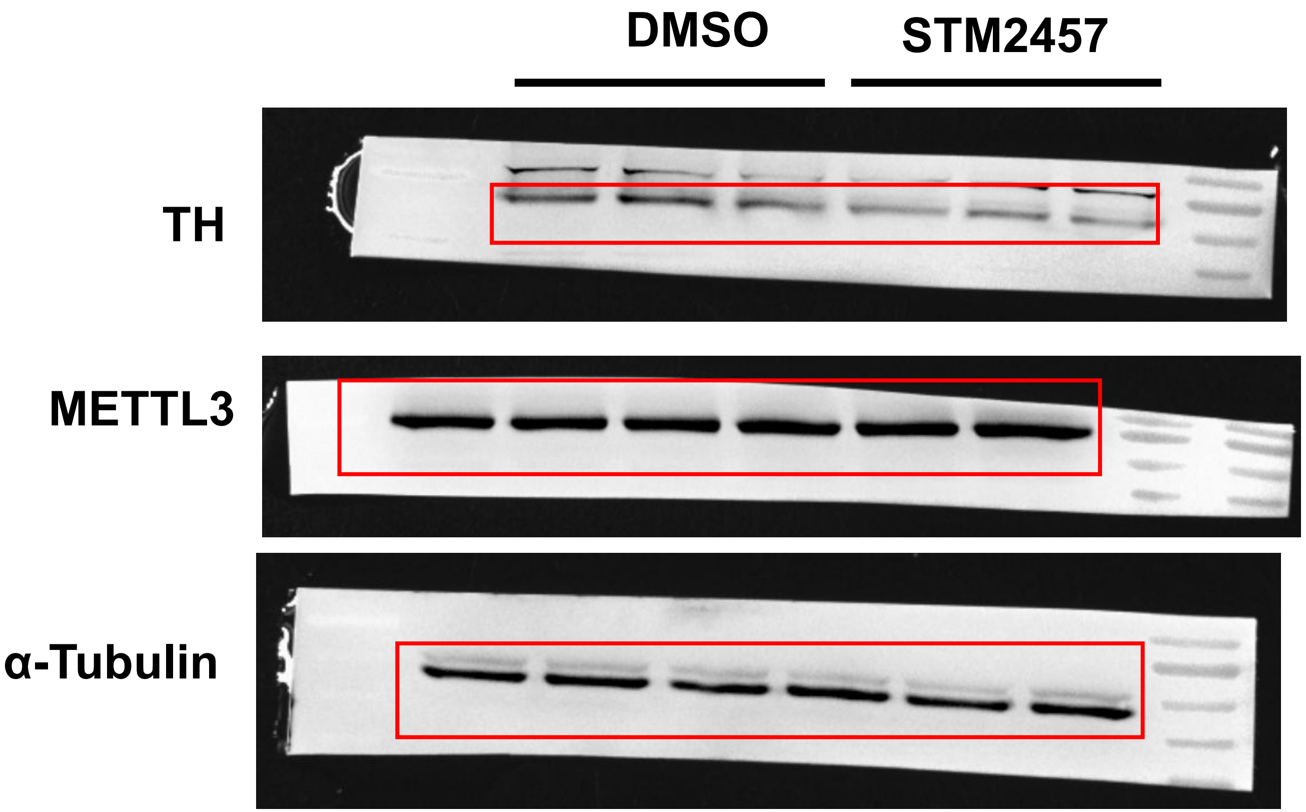

Full unedited blot for Supplemental Figure 3E

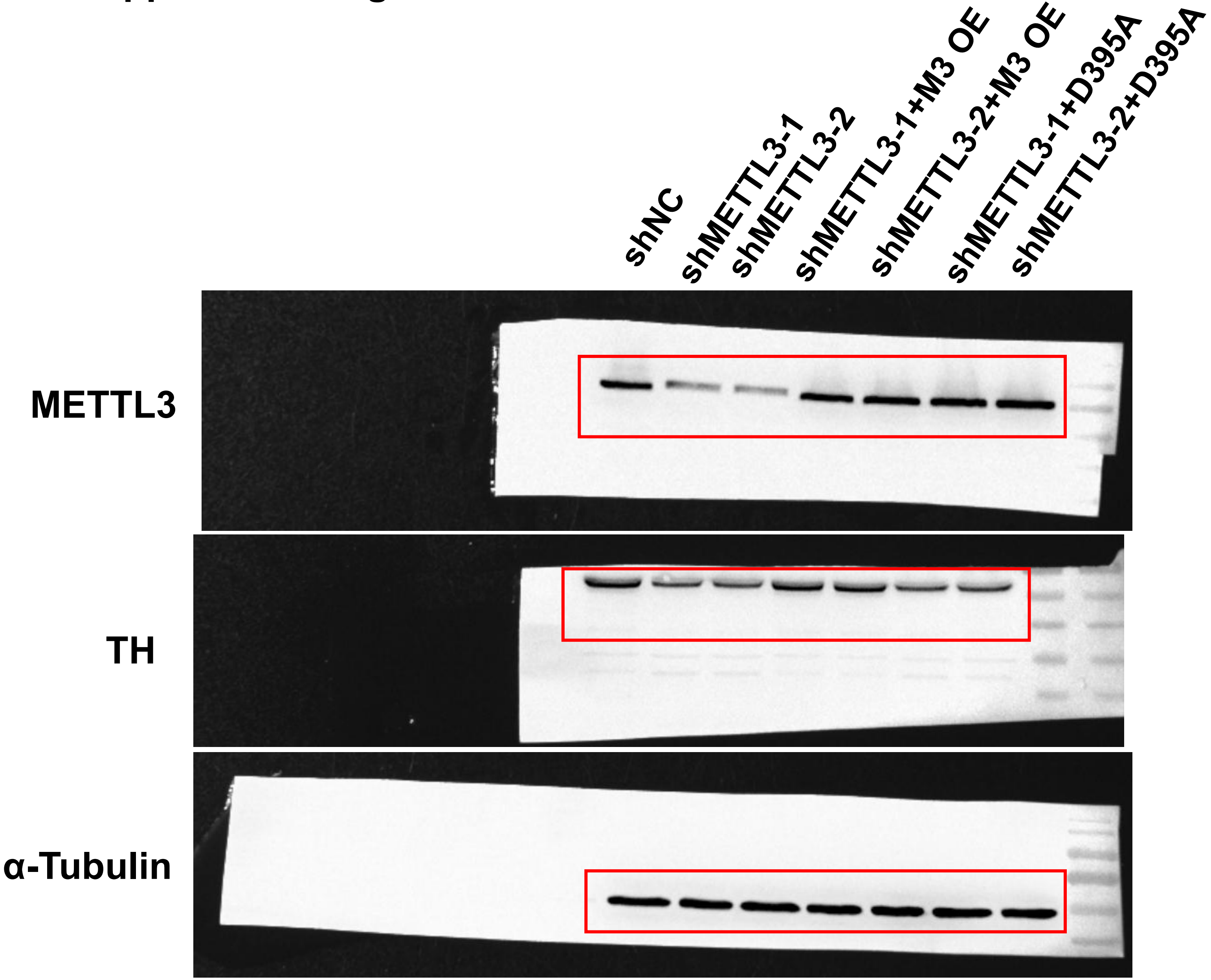

Full unedited blot for Supplemental Figure 4K

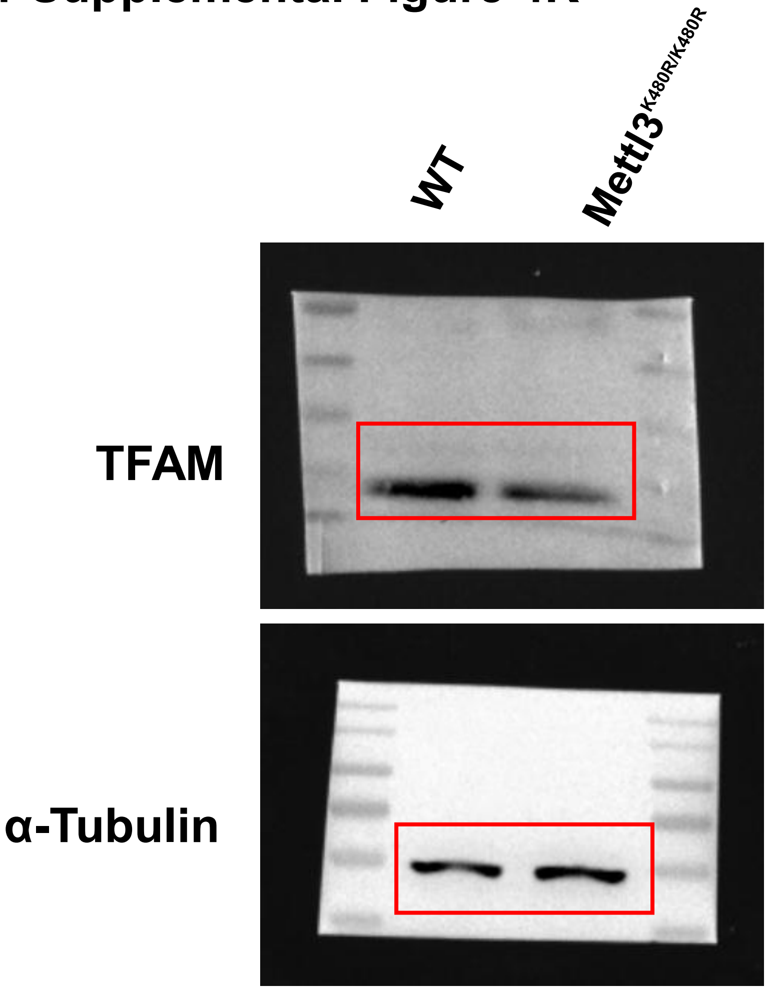

Full unedited blot for Supplemental Figure 5A

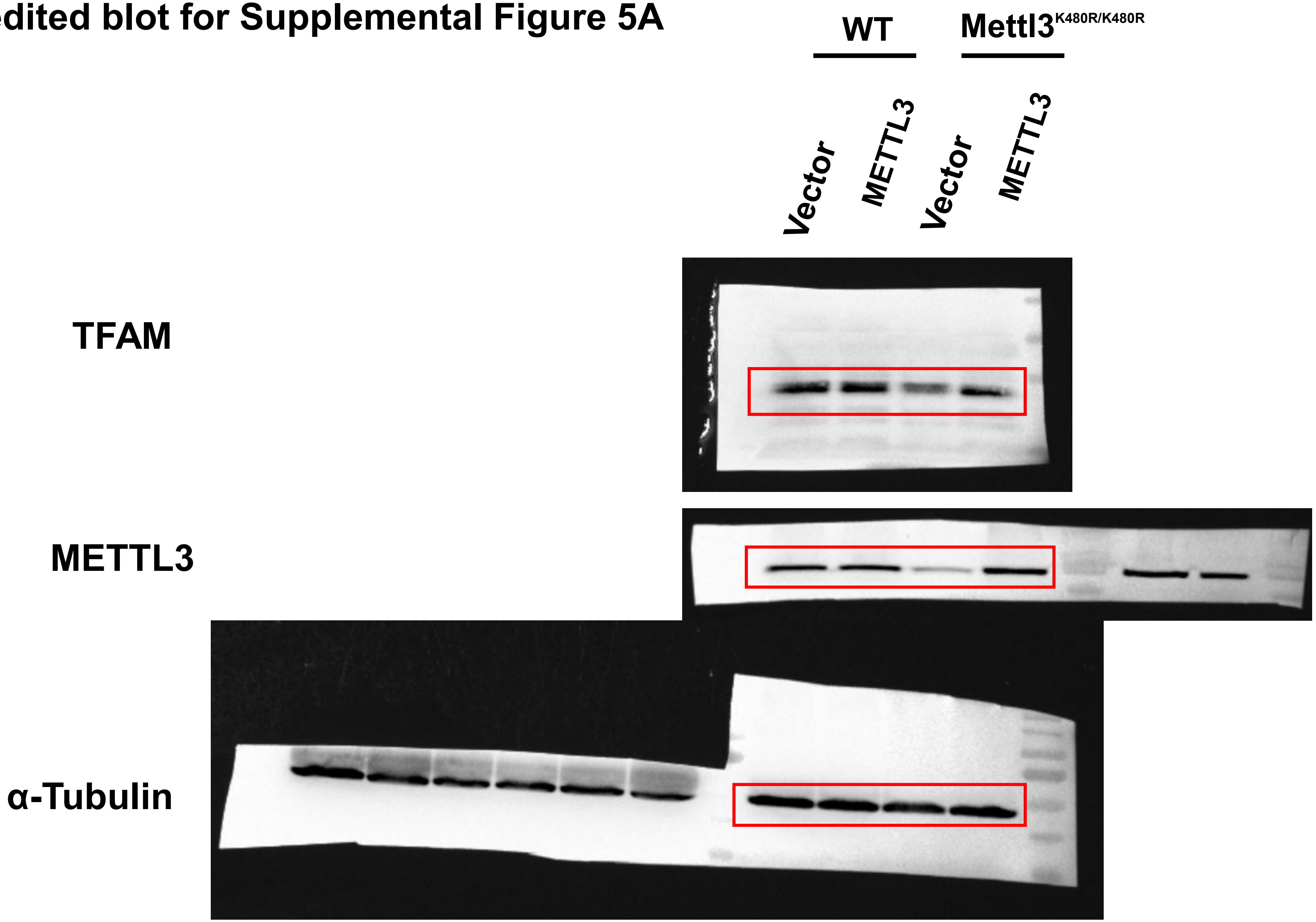

Full unedited blot for Supplemental Figure 5D

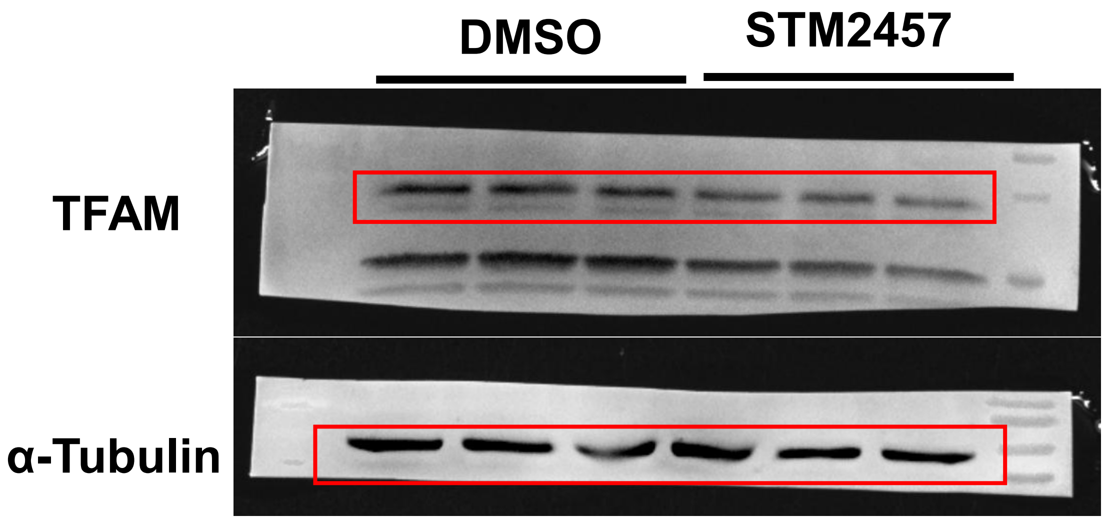

Full unedited blot for Supplemental Figure 5H

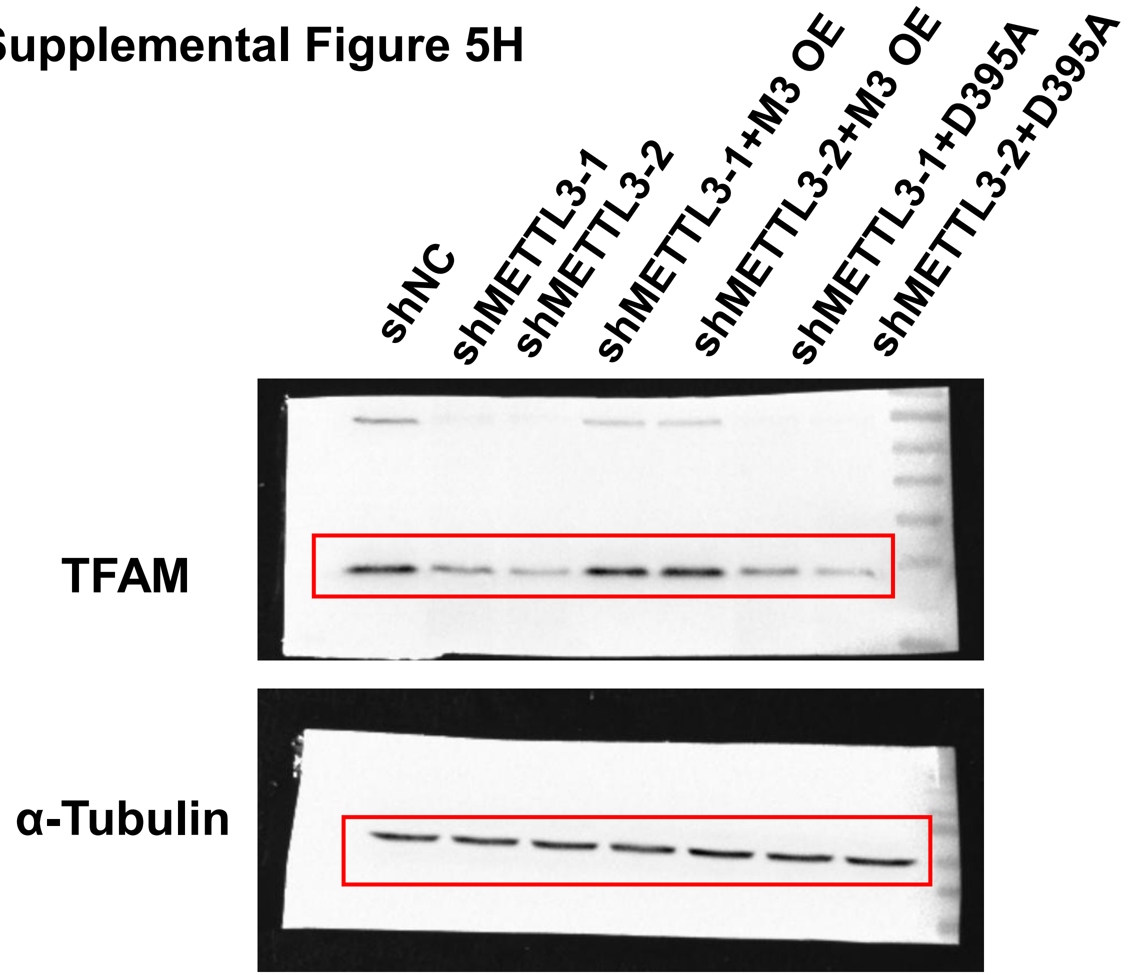

Full unedited blot for Supplemental Figure 5L

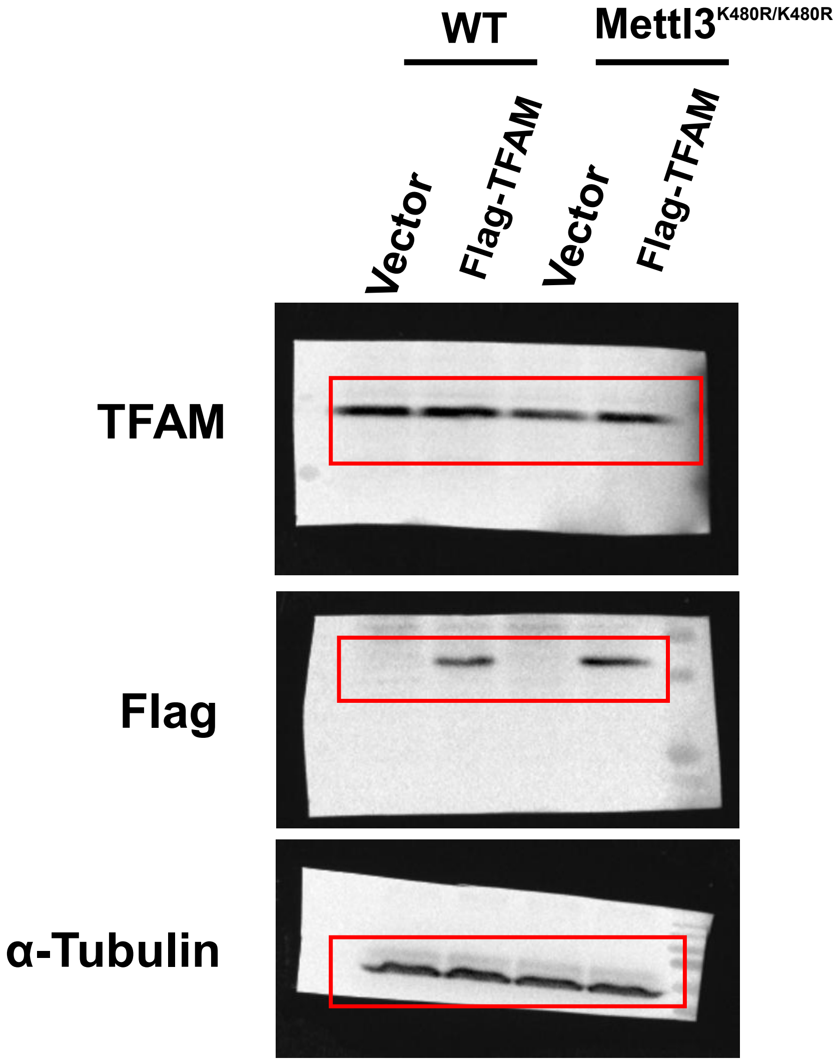

Full unedited blot for Supplemental Figure 6H

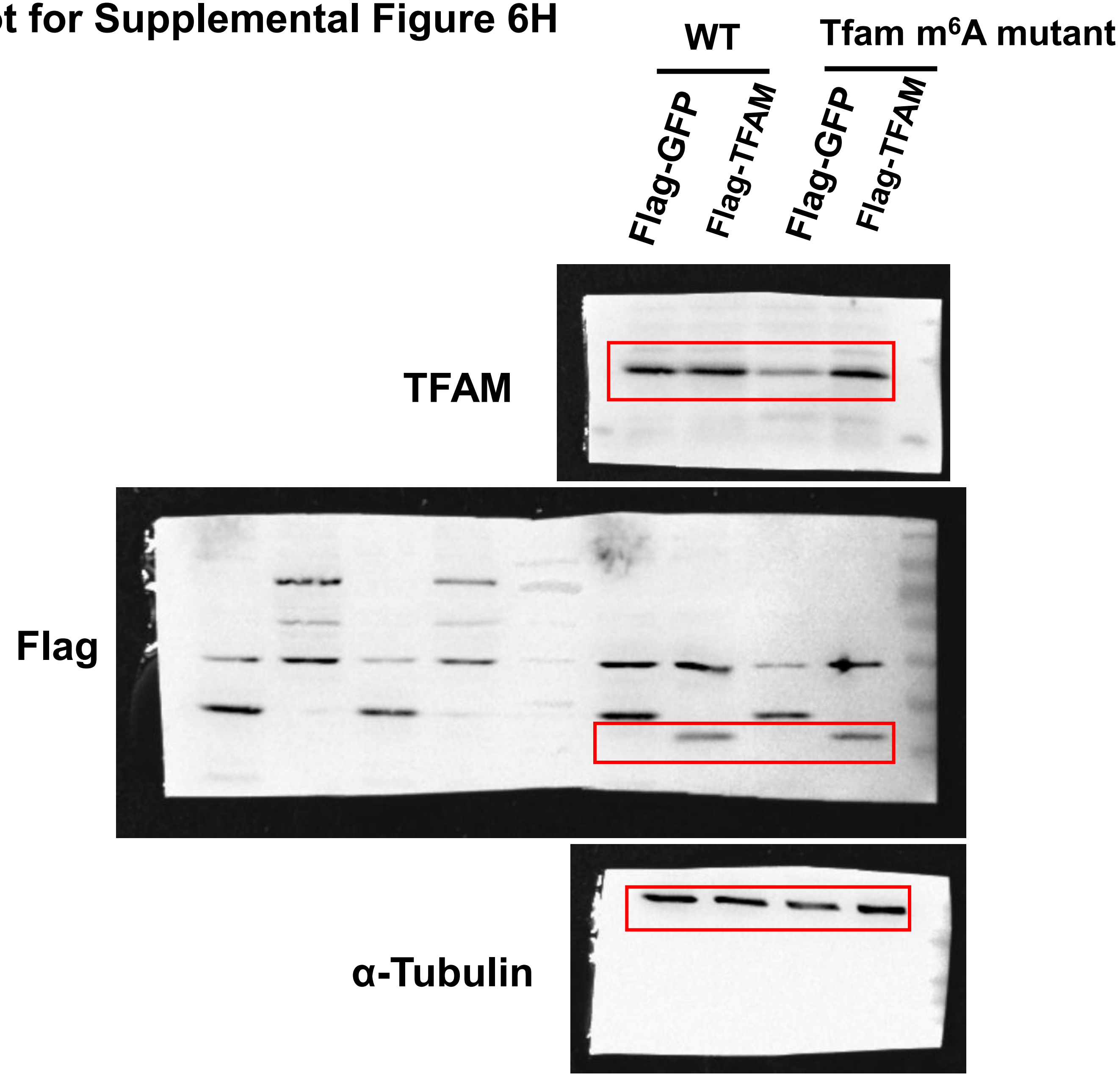

Full unedited blot for Supplemental Figure 6K

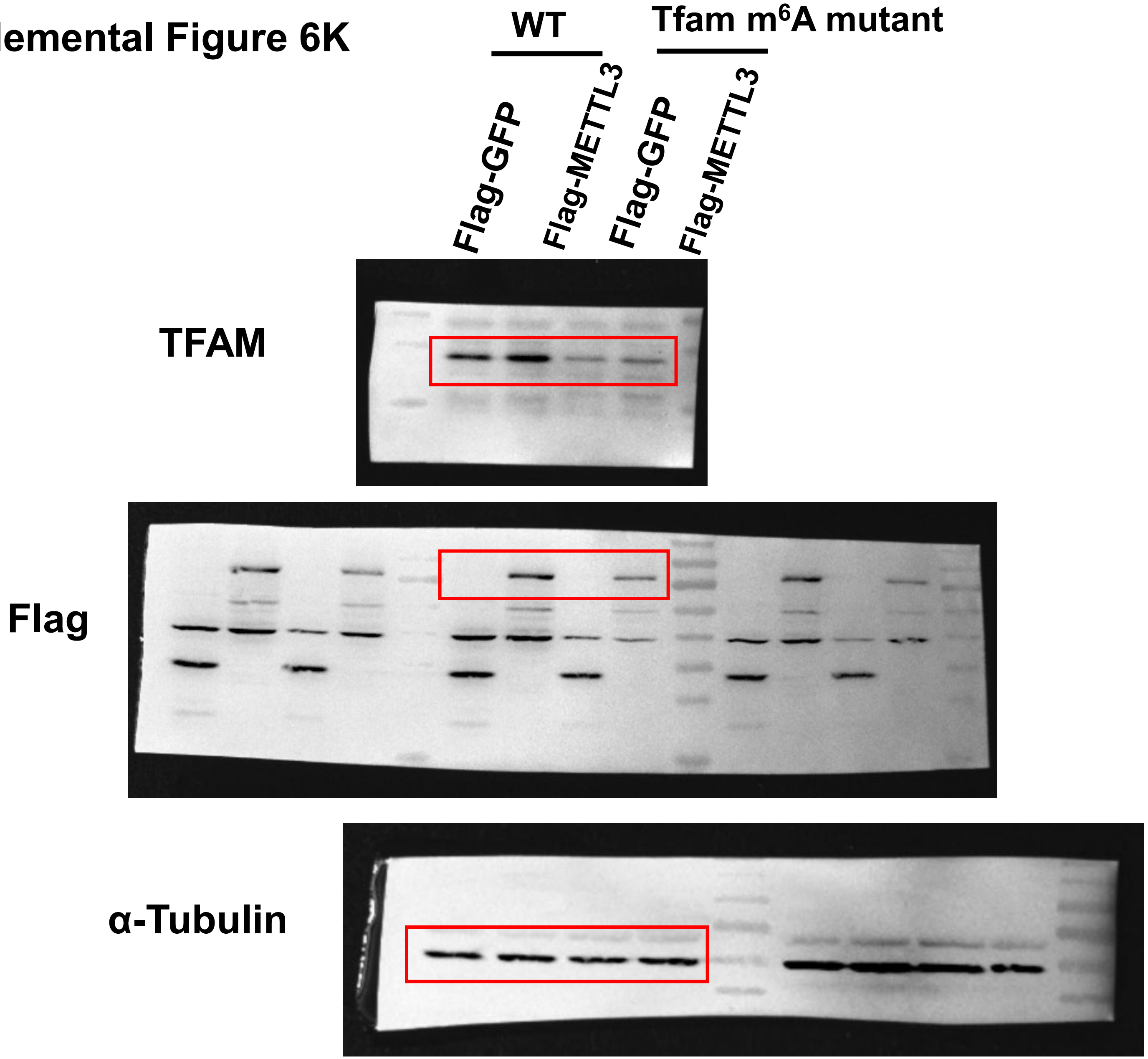

Full unedited blot for Supplemental Figure 6R

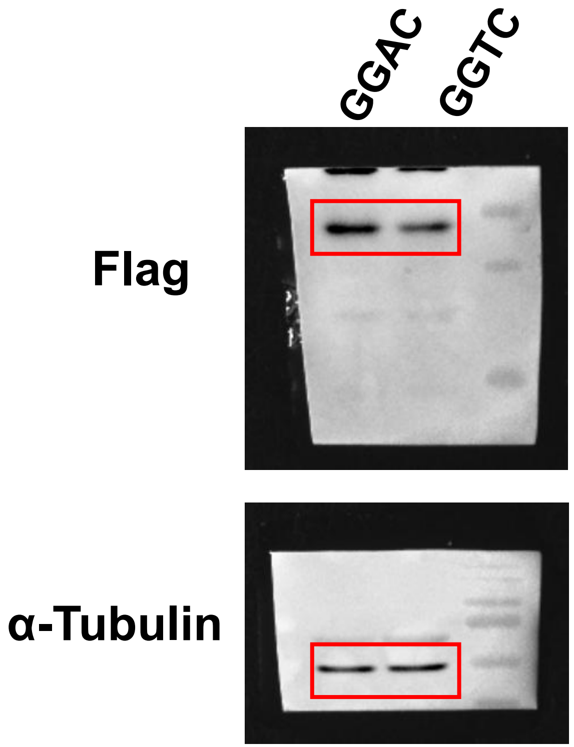

Full unedited blot for Supplemental Figure 6V

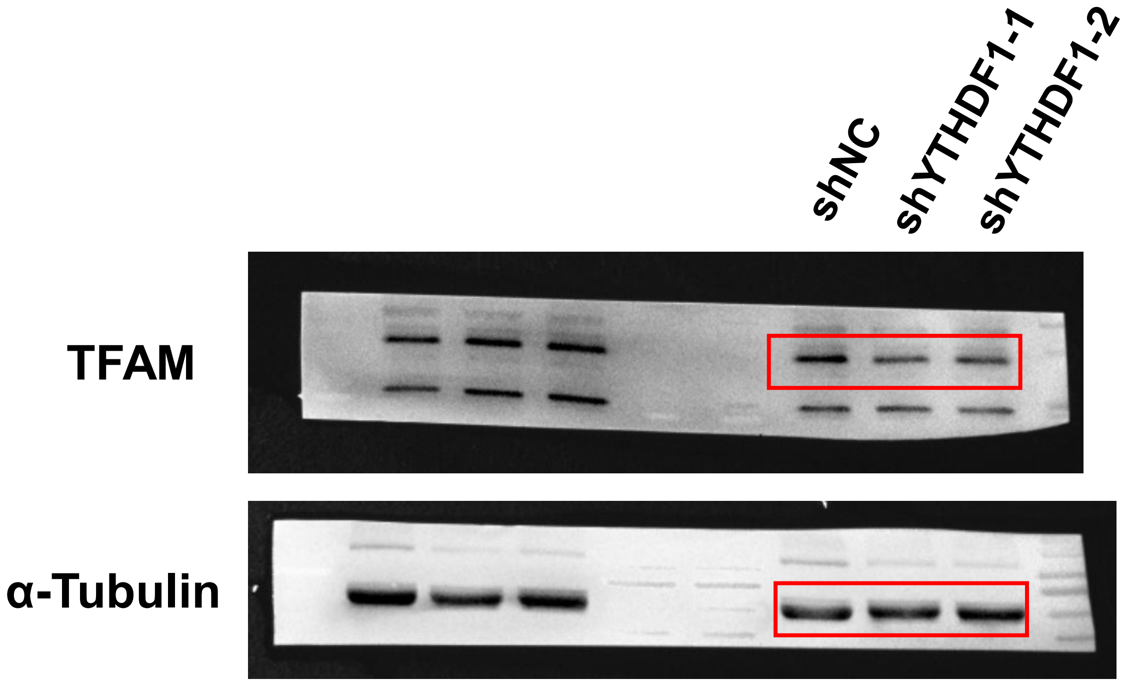

Full unedited blot for Supplemental Figure 7A

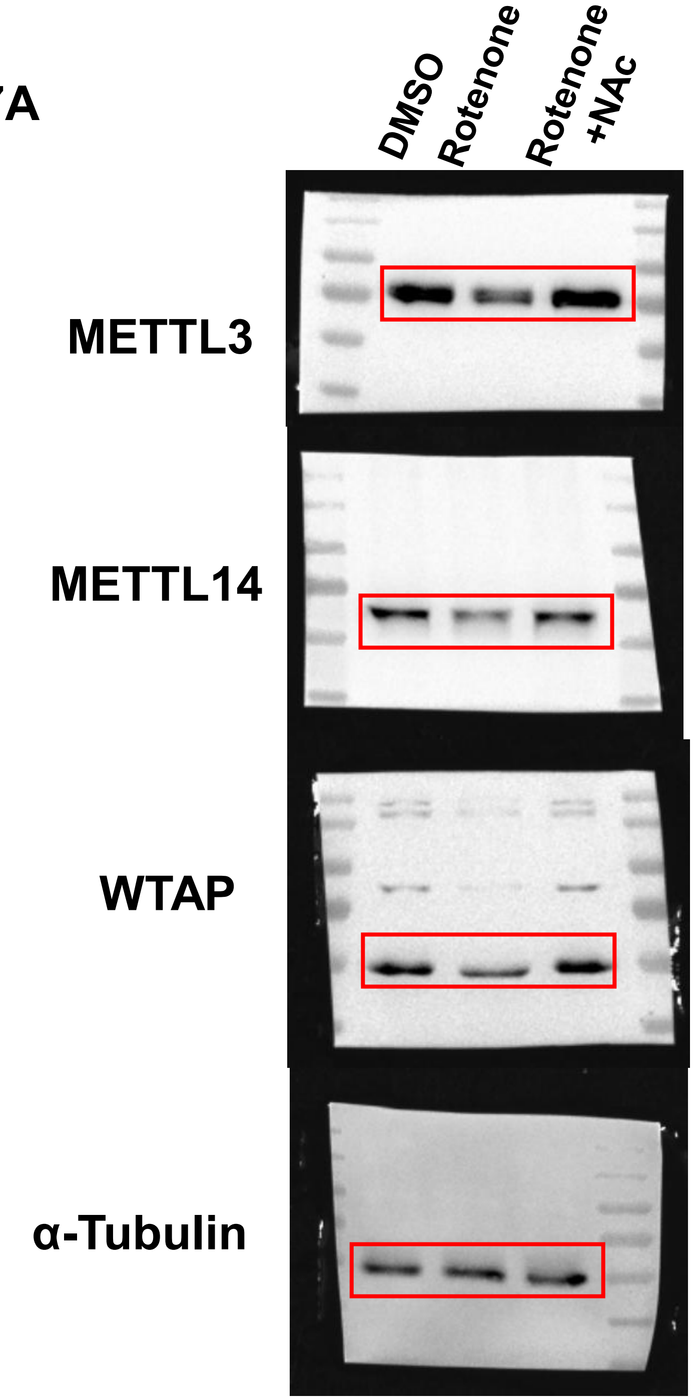

Full unedited blot for Supplemental Figure 8B

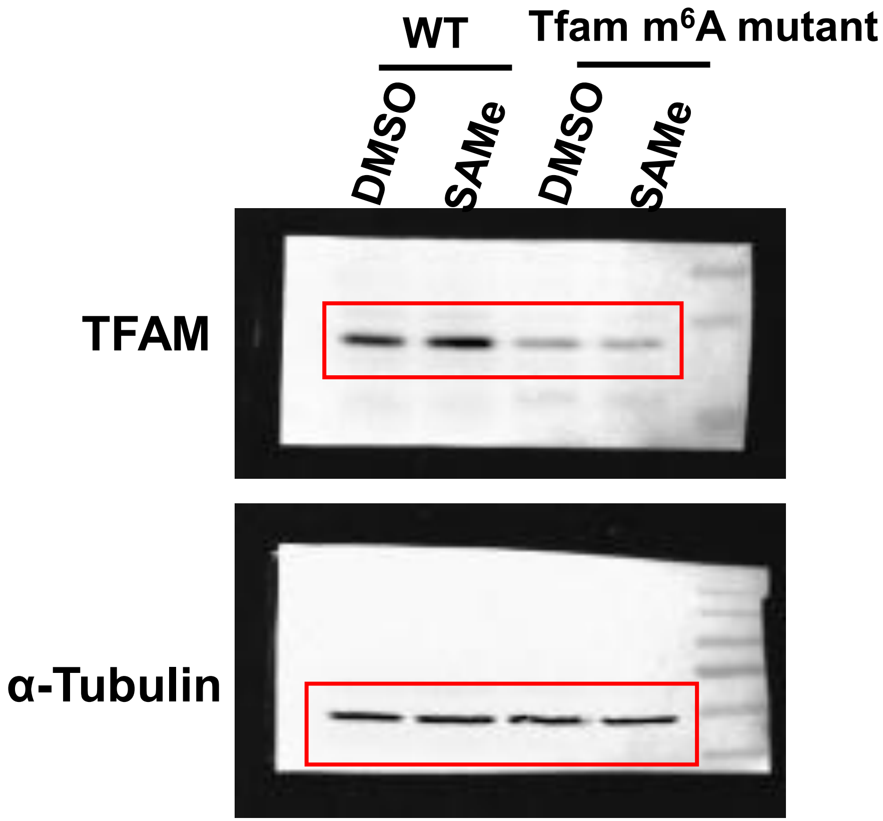

Supplement: Unedited blot and gel images [file jci-136-197183-s313.pdf]
